# A metal–peptide capsule by multiple ring threading

**DOI:** 10.1038/s41467-019-13594-4

**Published:** 2019-12-12

**Authors:** Tomohisa Sawada, Yuuki Inomata, Koya Shimokawa, Makoto Fujita

**Affiliations:** 1 Department of Applied Chemistry, School of Engineering, The University of Tokyo, 7-3-1 Hongo, Bunkyo-ku, Tokyo, 113-8656 Japan; 2 Department of Mathematics, Saitama University, 255 Shimo-Okubo, Sakuraku, Saitama, 338-8570 Japan; 3 Division of Advanced Molecular Science, Institute for Molecular Science, National Institutes of Natural Sciences, 5-1 Higashiyama, Myodaiji-cho, Okazaki, Aichi, 444-8787 Japan

**Keywords:** Interlocked molecules, Molecular capsules, Self-assembly, Molecular self-assembly

## Abstract

Cavity creation is a key to the origin of biological functions. Small cavities such as enzyme pockets are created simply through liner peptide folding. Nature can create much larger cavities by threading and entangling large peptide rings, as learned from gigantic virus capsids, where not only chemical structures but the topology of threaded rings must be controlled. Although interlocked molecules are a topic of current interest, they have for decades been explored merely as elements of molecular machines, or as a synthetic challenge. No research has specifically targeted them for, and succesfully achieved, cavity creation. Here we report the emergence of a huge capsular framework via multiple threading of metal–peptide rings. Six equivalent *C*_4_-propeller-shaped rings, each consisting of four oligopeptides and Ag^+^, are threaded by each other a total of twelve times (crossing number: 24) to assemble into a well-defined 4 nm-sized sphere, which acts as a huge molecular capsule.

## Abstract (web summary)

Constructing molecular cages from entangled molecules is a complex task requiring precise topological control. Here, the authors thread together six metal-peptide rings into a giant cubic molecular capsule with a defined cavity and 24 crossover points.

## Introduction

**Fig. 1:**
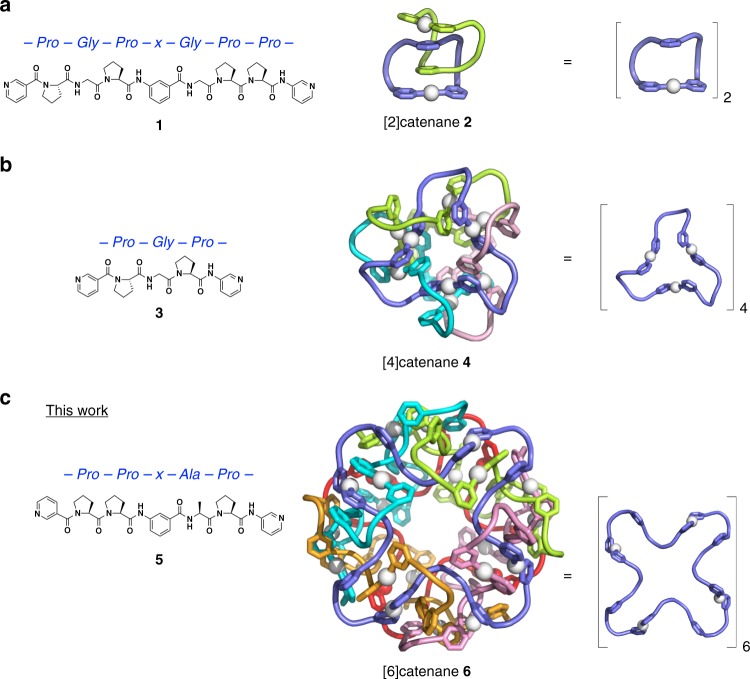
Pyridine-tethered oligopeptide ligands and their self-assembled structures on coordination to Ag^+^. **a** Peptide ligand **1** with a PGPxGPP sequence (x: imino-(1,3-phenylene)carbonyl spacer) gives [2]catenane **2**, which is composed of two Ag_1_(**1**)_1_ macrocycles (crossing number: 2). **b** Peptide ligand **3** with a PGP sequence forms [4]catenane **4**, which contains four Ag_3_(**3**)_3_ macrocycles fully interlocked in tetrahedral manner (crossing number: 12). **c** Peptide ligand **5** with a PPxAP sequence affords [6]catenane **6**, which is composed of six Ag_4_(**5**)_4_ macrocycles multiply interlocked in a cubic manner (crossing number: 24). The peptide regions are drawn as cartoon loops, and the pyridyl and *m*-phenylene parts in stick representation. Individual macrocycles linked by coordination to Ag^+^ (white spheres) are colour-coded.

The spherical shell structure of the HK97 virus capsid^1^ is made up of a large number of peptide entanglements and provides a lesson in the simplicity and elegance of design. A thin capsular shell provides the superstructure with mechanical stability and encloses its huge inner cavity. In this virus capsid, the essence of the shell formation process can be described as concomitant intra-strand (folding) and inter-strand (entwining) self-assembly processes, and this has inspired us to demonstrate the same using chemistry^2–7^. Previously, the self-assembly of Ag^+^ and ditopic oligopeptide **1** has resulted in peptide [2]catenane **2** (Fig. 1a)^4^. The Pro-Gly-Pro-x-Gly-Pro-Pro sequence (Pro: l-proline, Gly: glycine, and x: imino-(1,3-phenylene)carbonyl spacer) of **1** adopted a loop conformation on coordination to Ag^+^ and assembled to form a simple link of two Ag_1_(**1**)_1_ macrocycles. In contrast, shorter peptide ligand **3** afforded peptide [4]catenane **4**, which is formed of four tetrahedrally linked macrocycles with 12 strand-crossing points^3^; this structure is described as the *T*_2_-tetrahedral link topology^7^ by the practical topological description method developed for DNA polyhedral catenanes^8^ in knot theory^9^ (Fig. 1b). The Ω-shaped loop of the Pro-Gly-Pro sequence is too short to connect the two terminal pyridines (py) of **3** through Ag^+^ coordination, and instead the *C*_3_-symmetrical Ag_3_(**3**)_3_ macrocycle is formed, which further assembles to give the tetrahedral link.

**Fig. 2:**
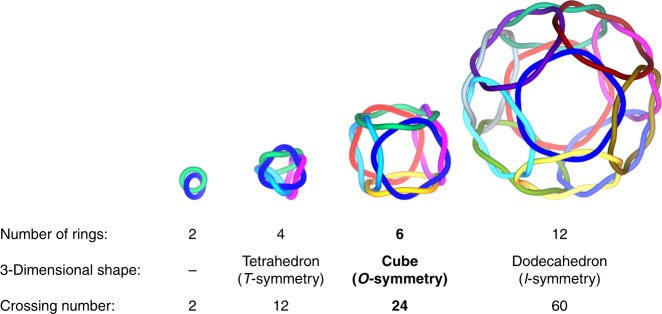
Series of polyhedral peptide [*n*]catenanes. Topological diagrams of a [2]catenane, tetrahedral [4]catenane, cubic [6]catenane, and dodecahedral [12]catenane.

This result prompted us to explore Pro-rich peptide fragments for the construction of a more advanced polyhedral link. Here, we report that Ag^+^ and ditopic Pro-Pro-x-Ala-Pro-sequenced (Ala: l-alanine) peptide **5** self-assemble to form peptide [6]catenane **6**, which is a cubic structure formed of six interlocking *C*_4_-symmetrical Ag_4_(**5**)_4_ macrocycles (Fig. 1c). This structure corresponds to the *T*_2_-hexahedral link, which belongs to a family of polyhedra formed from double lines with two twist (*T*_2_) operations on each edge^8^ (Fig. 2). This unique topology provides a huge isolated cavity (*ca* 3200 Å^3^); thus, **6** can be considered a miniature mimic of the HK97 virus capsid.

## Results

### Self-assembly of [6]catenane 6

**Fig. 3:**
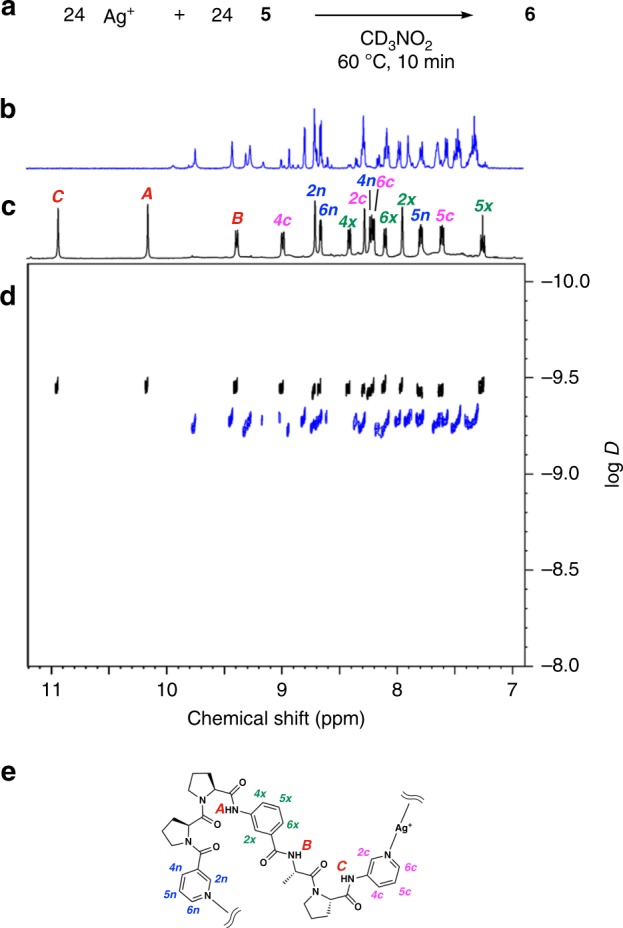
^1^H NMR and ^1^H DOSY NMR spectra (500 MHz, 300 K) of peptide [6]catenane 6 and its building block 5. **a** Complexation scheme. **b** ^1^H NMR spectrum of **5** in CD_3_NO_2_, and **c** that of **6** (Tf_2_N^−^ salt). **d** Overlaid ^1^H DOSY NMR spectra (aromatic region) of **5** (blue) and **6** (Tf_2_N^−^ salt, black) in CD_3_NO_2_. **e** Proton labelling used for the signal assignment in c.

Ditopic peptide ligand **5** was synthesised by solution-phase peptide synthesis (Supplementary Methods), and peptide [6]catenane **6** was obtained by simple mixing of **5** (14 μmol) and silver bis(trifluoromethanesulfonyl)imide (AgTf_2_N, 14 μmol) in CD_3_NO_2_ (0.7 mL) at 60 °C for 10 min (Fig. 3a). The ^1^H nuclear magnetic resonance (NMR) spectrum of the resulting mixture clearly showed that the multiple conformers of flexible **5** converged to a single conformation (Fig. 3b, c). Diffusion-ordered NMR spectroscopy (DOSY) revealed that these symmetrical proton signals were derived from a large monodisperse assembly at diffusion coefficient *D* = 3.4 × 10^–10^ m^2^•s^–1^ (log *D* = −9.45), and could be clearly distinguished from those of building block **5** at *D* = 5.3 × 10^–10^ m^2^•s^–1^ (log *D* = −9.26) (Fig. 3d, e and Supplementary Fig. 31). The formation of a monodisperse assembly was also supported by dynamic light scattering (DLS) analysis (Supplementary Fig. 42) and ion-mobility mass spectrometry (IM-MS)^10^ (Supplementary Figs. 43 and 44), and its framework showed good thermal stability at 50 °C; this was confirmed by variable temperature NMR spectroscopy (Supplementary Fig. 30). The quantitative self-assembly of **6** was very fast; it was completed within 2 min by simply stirring the components at room temperature. This suggests that the folding and entwining are not stepwise, but simultaneous processes.

**Fig. 4:**
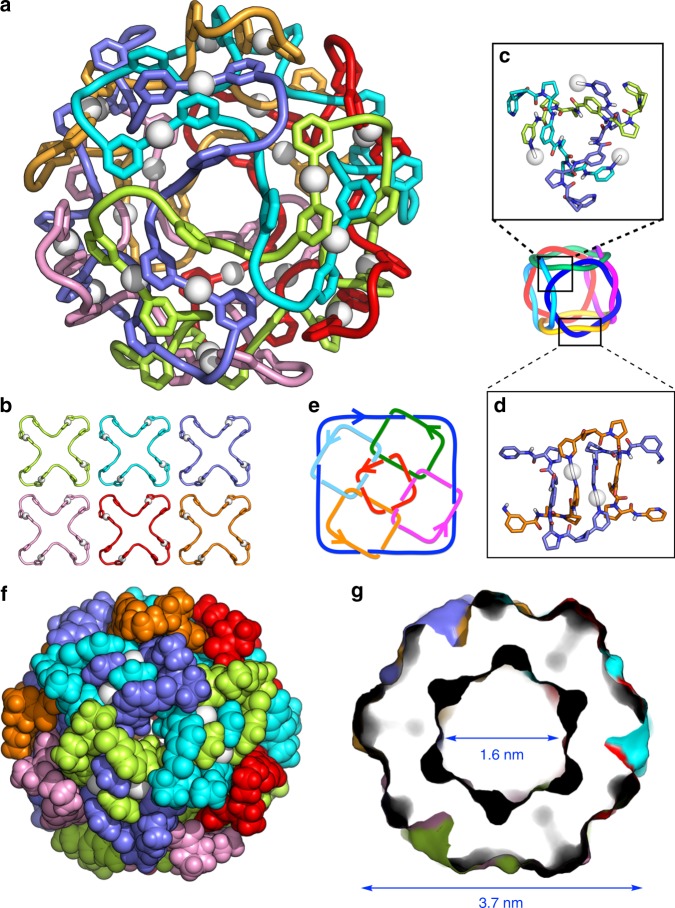
X-ray crystal structure of peptide [6]catenane 6. **a** Cartoon loop representation of **6** viewed along the *C*_3_ axis. **b** The six equivalent *C*_4_-propeller-shaped Ag_4_(**5**)_4_ macrocycle components of **6** are disassembled and shown individually. **c** A three-way junction structure in **6**, which corresponds to a vertex of the *T*_2_-hexahedral link topology. **d** A *T*_2_-twist structure in **6**, which corresponds to an edge of the topology. The white balls indicate Ag^+^. **e** Schematic diagram of the [6]catenane framework topology. The arrows on each ring indicate the N→x→C direction of the peptide ligand. **f** Space-filling representation of **6** showing the closed-shell exterior. **g** Cross-sectional view of the surface representation of **6**. In all figures, anions are omitted for clarity and topologically-linked macrocycles are individually colour-coded.

Distinct structural evidence for the formation of peptide [6]catenane **6** was obtained by a single crystal X-ray diffraction study. Slow vapour diffusion of diisopropyl ether into a CH_3_NO_2_ solution of **6** (40 mM) at room temperature over 10 days afforded single crystals. Structural analysis carried out in the cubic *I*432 space group clearly revealed the molecular structure of **6** in high resolution (Fig. 4a). Another crystallographic data analysed in the monoclinic *I*2 showed the structural flexibility of **6**: the molecular structure was identical except for the slightly flattened conformation from the spherical shape (Supplementary Fig. 36). Each peptide ligand adopts the same specific “S-curve” conformation to give a *C*_4_ propeller-shaped Ag_4_(**5**)_4_ macrocycle through linear coordination of Ag^+^ to the terminal py on **5** (Fig. 4b and Supplementary Fig. 41). Six of these macrocycles form a cubic assembly through ring threading at each peptide hairpin; this corresponds to the *T*_2_-hexahedral link. Although the linking of two directional (chiral) macrocycles can generate diastereomers in principle, the ^1^H NMR observation of the single product indicates the threading process takes place with complete diastereo-selectivity here as observed in the previous study on **2**^4^. Each vertex (three-way junction structure) of this topology is induced by six sets of inter-strand amide hydrogen bonds (Fig. 4c and Supplementary Fig. 40). Each edge (twisted double loops) is stabilised by four sets of amide hydrogen bonds in addition to two Ag–π interaction arrays (Fig. 4d and Supplementary Fig. 39). This is also observed in previous [2]catenane **2**^4^. The crossing number of this topology is 24 (2 × 12 edges), and this was clearly confirmed by the reduced alternating link diagram^9^ shown in Fig. 4e. This crossing number is considerably higher than that of the *T*_2_-tetrahedral link in previous [4]catenane **4** or that of its topological analogue (both have crossing numbers of 12)^3,7^. To the best of our knowledge, no discrete synthetic molecules have been synthesised with such a large crossing number to date ^11–14^.

### Modification of inner cavity

**Fig. 5:**
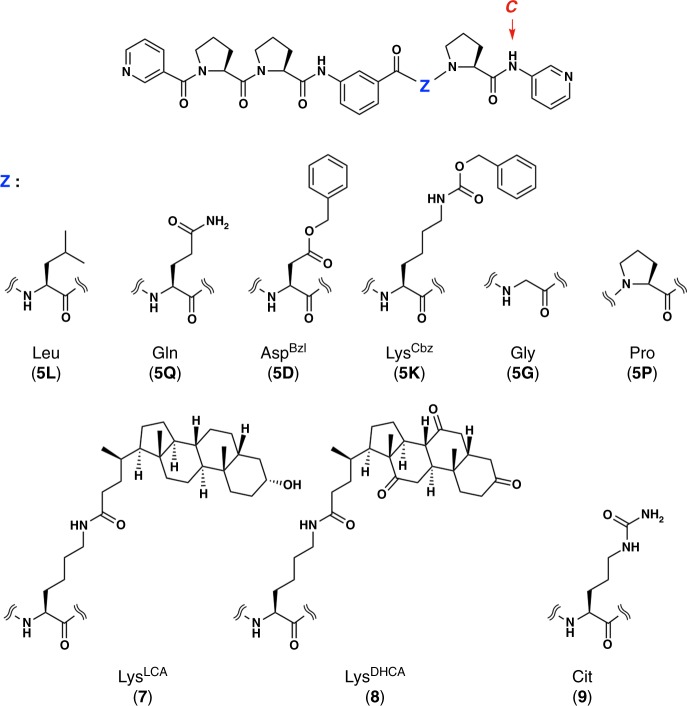
Peptide ligands with various residues at the Z position. Chemical structures of peptide ligands **5L**, **5Q**, **5D**, **5K**, **5G**, **5P**, **7**, **8** and **9**.

**Fig. 6:**
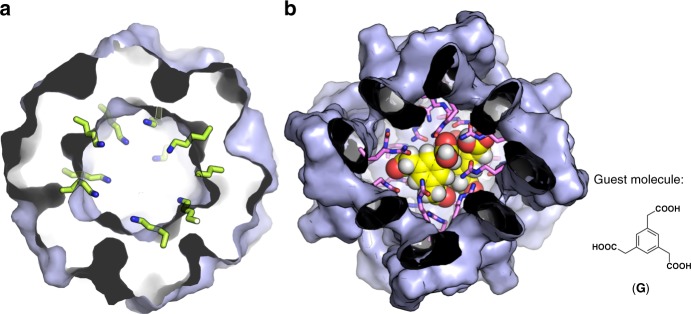
Inner modification and guest encapsulation within the [6]catenane framework. **a** Estimated model of the side chain positions at the Z residue using ligand **5K** as an example. Lys side chains are highlighted in stick representation and the remaining [6]catenane framework (surface representation) is a cross-sectional view. Note that all of the Cbz protecting groups and some of the Lys side chains have been omitted for clarity. **b** Geometry-optimised structure of encapsulation complex [Ag_24_(**9**)_24_]^24+^•(**G**)_2_. The [6]catenane framework, all the side chains of Cit residues, and **G** are shown in surface, stick, and space-filling representations, respectively. The chemical structure of **G** is also shown.

**Fig. 7:**
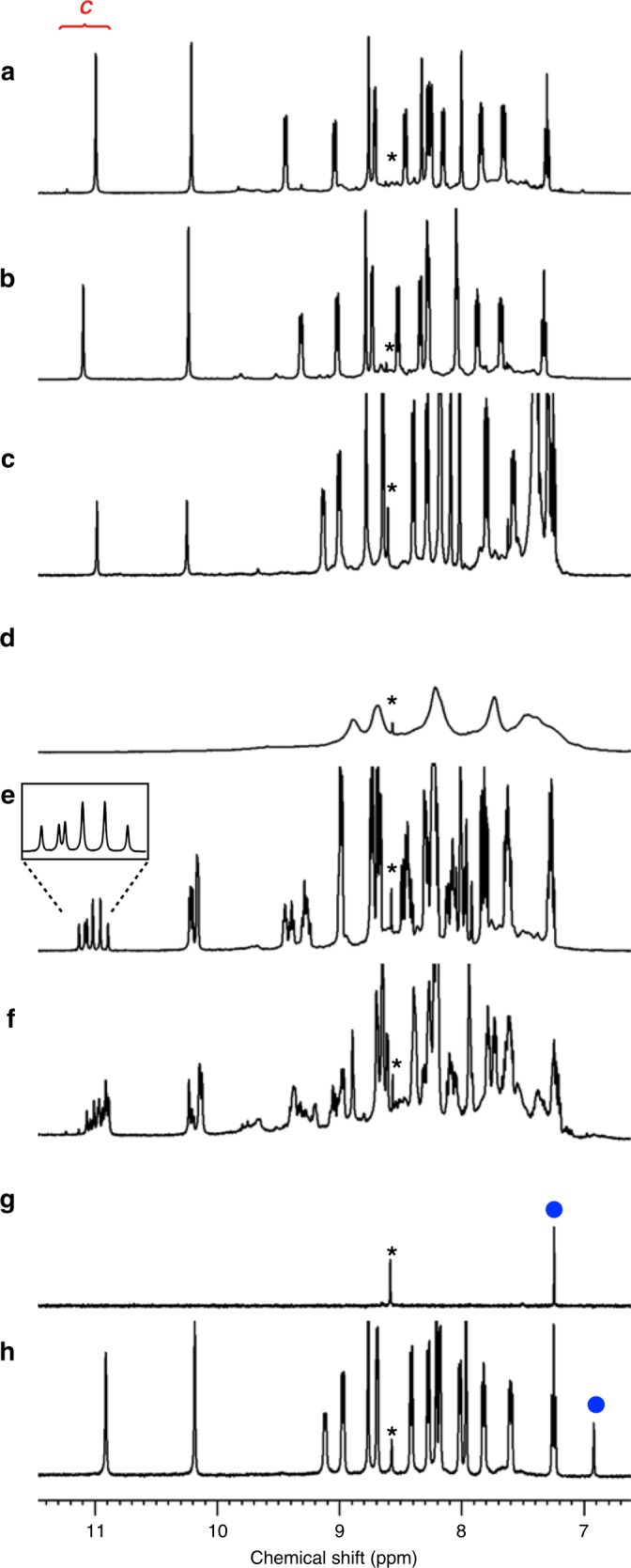
^1^H NMR spectra after complexation of peptide ligands with various residues at the Z position and Ag^+^. (aromatic region, CD_3_NO_2_, 500 MHz, 300 K) **a** [6]catenane **6**, and the mixture after complexation of **b** **5L** +Ag^+^, **c** **5K** +Ag^+^, **d** **5G**+Ag^+^, **e** **5+5L**+Ag^+^ (1:1:2), and **f** **5**+**7**+Ag^+^ (3:1:4). **g** **G** only, and **h** the encapsulation complex after complexation of **9**+Ag^+^ +**G** (1:1:0.04). Signals with blue circles indicate the aromatic proton signal of **G**. Signals with asterisks derive from the solvent.

Another special feature of **6** is its huge inner cavity. The exquisite entwining of peptide strands affords a capsular shell (Fig. 4f) with a highly isolated 1.6-nm-sized cavity (Fig. 4g). The framework contains only 0.4-nm-sized pinholes as the static form in the solid state. Engineering of the inner surface of **6** was achieved simply by replacing the Ala residue position (Z) on the ligand with other α-amino acids (Figs. 5 and 6a). We confirmed that the l-leucine (Leu) analogue, ligand **5L**, showed the same ^1^H NMR signal pattern as **6** after coordination to Ag^+^ (Fig. 7ab), which indicates the formation of the [6]catenane of the Ag_24_(**5L**)_24_ composite. Likewise, ^1^H NMR observation confirmed that analogous ligands, **5Q** (Z: l-glutamine (Gln)), **5D** (Z: l-aspartic acid β-benzyl ester (Asp^Bzl^)), and **5K** (Z: *N*^*ε*^-benzyloxycarbonyl l-lysine (Lys^Cbz^)) also successfully assembled into the [6]catenanes (Fig. 7c for **5K**, Supplementary Fig. 27b–e). In sharp contrast, replacement of the Ala residue with Gly (ligand **5G**) or Pro (ligand **5P**) residues did not result in convergent spectra. This is ascribed to the high conformational flexibility of Gly and the rigidity of Pro, which prevented the stable formation of the S-shaped conformation (Fig. 7d for **5G**, Supplementary Fig. 27h for **5P**). Reaction of an equimolar mixture of ligands **5** (1 eq.) and **5L** (1 eq.) with AgPF_6_ (2 eq.) gave a ^1^H NMR spectrum with six signals (integral ratio: 1:1:1:2:2:1) for the C-terminal amide proton at around 11 ppm (Fig. 7e), which indicates ligand swapping on the [6]catenane framework. We suggest that one plausible structure that would provide this spectrum is a *C*_3_-symmetric [6]catenane containing equimolar amounts of **5** and **5L** ligands (Supplementary Table 5).

### Guest encapsulation

Inner modification with Lys residues enabled large guest loading within the cavity. Although molecular encapsulation in small spaces has gradually been reported^15^, guest confinements within huge cavities (>1000 Å^3^) are challenging because of the less efficient contacts between the guest and the cavity. To demonstrate this, lithocholic acid (LCA)-tethered peptide ligand **7** was prepared (Fig. 5, Supplementary Methods), and a mixture of ligands **5** (3 eq.) and **7** (1 eq.) was reacted with Ag^+^ (4 eq.). After complexation, the C-terminal amide proton signals were again observed in the downfield (11.2–10.9 ppm) region of the ^1^H NMR spectrum owing to severe desymmetrisation of the structure (Fig. 7f, Supplementary Fig. 49). With the support of DOSY NMR spectroscopy and modelling studies, we estimated that the cavity volume of the [6]catenane framework was large enough to confine six LCA molecules (occupied volume by guests: 6 × 370 Å^3^ = 2220 Å^3^) (Supplementary Figs. 51a and 55). Likewise, incorporation of dehydrocholic acid (DHCA) within the cavity using ligand **8** was also successful (Supplementary Figs. 50 and 51b). These demonstrations show not only the strategy for guest loading within huge cavities but the potential applicability of various bioconjugation techniques^16^ for the inner modifications.

Furthermore, guest encapsulation in a non-covalent manner was also confirmed. To engineer guest-binding sites, the l-citrulline (Cit) analogue, ligand **9**, was prepared (Fig. 5, Supplementary Methods). We expected that reversible encapsulation^17,18^ occurred even for this mechanically interlocked capsule if the inner surface of the cavity is modified by densely integrated urea groups and induce multiple H-bonds formation with a guest molecule. After the complexation of ligand **9** (24 eq.) and Ag^+^ (24 eq.) under the existence of 1,3,5-benzenetriacetic acid **G** (1 eq.) as a guest, the ^1^H NMR spectrum showed the signal pattern of the successful [6]catenane formation (Ag_24_(**9**)_24_) in addition of the significant upfield shifts of the signals of **G** (Fig. 7g, h). In contrast, the unmodified framework **6** showed the weak interaction with **G** (Supplementary Figs. 52 and 53). The observation on downfield shifts of urea proton signals as increasing ratio of **G** indicates the H-bonds formation of the urea with the carboxyl groups on **G** (Supplementary Fig. 54). Significantly, the rapid guest exchange between the inside and outside of the capsular framework observed in DOSY NMR studies (Supplementary Fig. 53) suggests that the capsular framework is not keeping the closed shell conformation but relatively dynamic nature in the solution state: the structural flexibility based on the mechanical entanglements in addition of the labile nature of the py–Ag^+^ coordination bond enables guest **G** to pass through the framework. Figure 6b shows the modelling structure, which shows at least two molecules of **G** can be encapsulated even in the closed shell framework. Thus, the interlocked metal–peptide frameworks show unique structural flexibility and therefore function to encapsulate guest molecules.

## Discussion

Another aspect about the mechanical entanglements to be discussed is the efficiency of the cavity construction. In the case of compound **6**, large cavity of *ca* 3200 Å^3^ volume (calculated by 3V^19^) was constructed by 120 amino acid building blocks (5 residues ×24 ligands) in total (Supplementary Fig. 58). This is quite small number of amino acid residues considering that natural or de novo proteins of around 120 residues possess at most 600–1200 Å^3^ volume cavities (Supplementary Table 6 and Supplementary Fig. 56). In other words, construction of a cavity comparable to 3000 Å^3^ volume generally requires polypeptides of 300–600 amino acid residues (Supplementary Table 7 and Supplementary Fig. 57). These facts suggest the rationality of the molecular entanglements for shaping the cavity.

In summary, we have succeeded in the chemical bottom-up construction of a metal–peptide capsule with multiple ring threadings. Although a single peptide strand or py–Ag^+^ bond is fragile even in non-coordinating solvents, its highly entwined structure, formed through self-assembly, forms a large hollow scaffold. In terms of topology, it is noteworthy that the metal-linked peptide rings select a single topology from an enormous number of possibilities; to date, the exact number of topologies for crossing number 24 cannot even be calculated by knot theory^20,21^. Furthermore, the emergence of the 24-crossing [6]catenane here provides us an inspiration for the next topology of the *T*_2_-polyhedral links, that a *C*_5_ propeller-shaped design of a metal–peptide ring will give a *T*_2_-dodecahedral link, that is a 60-crossing [12]catenane (Fig. 2). Including different types of topologies by a symmetry-based prediction^22^, it will bring growing attentions for chemical constructions of advanced polyhedral links. In terms of function, elaborate engineering of the inner surface of the cavity is possible in this capsular framework, unlike in most previous examples of molecular capsules^17,18,23–31^, which will enable the future incorporation of various active centres and the creation of artificial enzymes. Thus, the results not only present the formation of a single chemical topology and its guest encapsulation behaviour, but are also expected to broadly inspire further work of great interest in the fields of both topology and encapsulation.

## Methods

### General information

Boc-Pro-OH, Boc-Ala-OH, Boc-Leu-OH•H_2_O, Boc-Gln-OH, Boc-Lys(Cbz)-OH, Boc-Gly-OH, Boc-Asp(OBzl)-OH, Boc-Cit-OH, 4N HCl solution (in 1,4-dioxane), 1-ethyl-3-(3-dimethylaminopropyl)carbodiimide hydrochloride (EDCI), 1-hydroxy-1*H*-benzotriazole monohydrate (HOBt), *O*-(7-aza-1*H*-benzotriazol-1-yl)-*N*,*N*,*N’*,*N’*-tetramethyluronium hexafluorophosphate (HATU), 1-hydroxy-7-azabenzotriazole (HOAt), and *N*,*N*-diisopropylethylamine (DIEA) were purchased from Watanabe Chemical Industries. 1,3,5-benzenetriacetic acid was purchased from Honeywell Fluka. Other reagents and solvents were purchased from TCI, FUJIFILM WAKO Pure Chemical Corporation, Sigma-Aldrich or Kanto Chemical. All the chemicals were of reagent grades and used without any further purification. All the NMR spectral data were recorded on a Bruker Avance 500 MHz spectrometer equipped with a CP-TCI cryoprobe, a Bruker Avance III HD 500 MHz spectrometer equipped with a PABBO probe or a JEOL 500 MHz spectrometer equipped with a ROYAL probe. Electrospray ionisation time-of-flight mass spectrometry (ESI-TOF-MS) data were recorded on a Bruker maXis. IM-MS data were recorded on a Bruker timsTOF Pro. The data analyses of mass spectra were processed on a Bruker DataAnalysis (Version 5.1) software. Recycling preparative size-exclusion chromatography (SEC) was carried out by using a LC-9260II NEXT (JAI) equipped with JAIGEL 1H and 2H columns (eluent: chloroform) or a JAIGEL W252 column (eluent: methanol or DMF/methanol) or a LABOACE (JAI) equipped with JAIGEL 2HR(×2) columns (eluent: chloroform). Analytical HPLC data were recorded on an EXTREMA (JASCO) equipped with an InertSustain NH2 column (GL Sciences). Melting points were determined on a Stanford Research Systems OptiMelt. Elemental analyses were performed at the Elemental Analysis Centre (School of Science, The University of Tokyo). DLS measurements were performed by using a Dynapro NanoStar (Wyatt Technology).

### Synthesis of ligands

Pentapeptide ligands **5**, **5L**, **5Q**, **5D**, **5K**, **5G**, **5P**, **7**, **8** and **9** were synthesised by solution-phase peptide synthesis. Detailed procedures and characterisation data of all compounds are shown in the Supplementary Methods and Supplementary Figs. 1–22.

### Formation of [6]catenane (6)

Two solutions of ligand **5** (9.5 mg, 14 µmol) in CD_3_NO_2_ (600 µL) and AgTf_2_N (6.0 mg, 14 µmol) in CD_3_NO_2_ (100 µL) were mixed in a test tube, and the mixture was stirred at 60 °C for 10 min. Quantitative formation of **6** was confirmed by ^1^H NMR (Supplementary Figs. 23–26, 28–31). The same procedure was also applied for the [6]catenanes formation from the other peptide ligands (Supplemantary Figs. 27 and 32).

Chemical shifts data of **6**•(Tf_2_N)_24_: ^1^H NMR (500 MHz, CD_3_NO_2_, 300 K), δ10.94 (s, 1H, Py(C)N*H*), 10.16 (s, 1H, xN*H*), 9.38 (d, 8 Hz, 1H, AlaN*H*), 8.99 (d, 9 Hz, 1H, Py(C)*H*_4′_), 8.71 (d, 1 Hz, 1H, Py(N)*H*_2′_), 8.66 (dd, 5 Hz, 1 Hz, 1H, Py(N)*H*_6′_), 8.41 (dd, 7.5 Hz, 1.5 Hz, 1H, x*H*_4′_), 8.28 (d, 2 Hz, 1H, Py(C)*H*_2′_), 8.23 (dd, 7.5 Hz, 1.5 Hz, 1H, Py(N)*H*_4′_), 8.20 (dd, 5 Hz, 1 Hz, 1H, Py(C)*H*_6′_), 8.10 (d, 7.5 Hz, 1H, x*H*_6′_), 7.95 (s, 1H, x*H*_2′_), 7.79 (dd, 7.5 Hz, 5 Hz, 1H, Py(N)*H*_5′_), 7.61 (dd, 8 Hz, 5 Hz, 1H, Py(C)*H*_5′_), 7.26 (t, 8 Hz, 1H, x*H*_5′_), 5.48 (m, 1H, Ala*H*_α_), 5.01 (t, 7 Hz, 1H, Pro(1)*H*_α_), 4.97 (m, 1H, Pro(2)*H*_α_), 4.82 (m, 1H, Pro(3)*H*_α_), 4.44, 4.01 (m, 2H, Pro*H*_δ_), 4.13, 3.93 (m, 2H, Pro*H*_δ_), 3.64–3.54 (m, 2H, Pro(1)*H*_δ_), 2.56, 2.4–1.9 (m, 12H, Pro*H*_β, γ_), 1.43 (d, 6.5 Hz, 3H, Ala*H*_β_); ^13^C NMR (125 MHz, CD_3_NO_2_, 300 K), δ172.4 (Ala*C*O), 172.2 (Pro(3)*C*O), 171.8 (Pro(2)*C*O), 171.3 (Pro(1)*C*O), 167.0 (x*C*O), 165.0 (Py(N)*C*O), 152.5 (Py(N)*C*_6′_), 149.2 (Py(N)*C*_2′_), 145.4 (Py(C)*C*_6′_), 143.0 (Py(C)*C*_2′_), 139.7 (x*C*_3′_), 138.4 (Py(N)*C*_4′_), 137.7 (Py(C)*C*_3′_), 134.7 (Py(N)*C*_3′_), 133.0 (x*C*_1′_), 128.8 (x*C*_5′_), 128.4 (Py(C)*C*_4′_), 126.0 (Py(N)*C*_5′_), 125.5 (Py(C)*C*_5′_). 122.4 (x*C*_4′_), 121.8 (x*C*_6′_), 118.6 (x*C*_2′_), 61.5–61.0 (Pro(2)*C*_α_, Pro(3)*C*_α_), 58.2 (Pro(1)*C*_α_), 50.2 (Pro(1)*C*_δ_), 48.2, 47.6 (Pro(2)*C*_δ_, Pro(3)*C*_δ_), 46.8 (Ala*C*_α_), 29.9, 29.5, 29.1 (Pro*C*_β_), 25.3, 24.8 (Pro*C*_γ_), 15.7 (Ala*H*_β_).

Chemical shifts data of [Ag_24_(**5L**)_24_]•(Tf_2_N)_24_: ^1^H NMR (500 MHz, CD_3_NO_2_, 300 K), δ11.04 (s, 1H, Py(C)N*H*), 10.18 (s, 1H, xN*H*), 9.26 (d, 8 Hz, 1H, LeuN*H*), 8.96 (d, 9 Hz, 1H, Py(C)*H*_4′_), 8.73 (s, 1H, Py(N)*H*_2′_), 8.68 (dd, 5 Hz, 1 Hz, 1H, Py(N)*H*_6′_), 8.47 (d, 8 Hz, 1H, x*H*_4′_), 8.28 (d, 8 Hz, 1H, Py(N)*H*_4′_), 8.23 (d, 2 Hz, 1H, Py(C)*H*_2′_), 8.22 (d, 5 Hz, 1H, Py(C)*H*_6′_), 7.99–7.98 (m, 2H, x*H*_6′_, x*H*_2′_), 7.81 (dd, 8 Hz, 5.5 Hz, 1H, Py(N)*H*_5′_), 7.62 (dd, 8.5 Hz, 5.5 Hz, 1H, Py(C)*H*_5′_), 7.29 (t, 8 Hz, 1H, x*H*_5′_), 5.50 (m, 1H, Leu*H*_α_), 5.15 (t, 7.5 Hz, 1H, Pro(1)*H*_α_), 4.95 (m, 1H, Pro(2)*H*_α_), 4.78 (m, 1H, Pro(3)*H*_α_), 4.3, 4.15, 3.95 (m, 4H, Pro*H*_δ_), 3.63–3.55 (m, 2H, Pro(1)*H*_δ_), 2.56, 2.4–1.9 (m, 12H, Pro*H*_β, γ_), 1.96, 1.75 (m, 2H, Leu*H*_β_), 1.43 (m, 1H, Leu*H*_γ_), 0.88 (d, 6.5 Hz, 3H, Leu*H*_δ_), 0.54 (d, 6.5 Hz, 3H, Leu*H*_δ′_).

Chemical shifts data of [Ag_24_(**5Q**)_24_]•(Tf_2_N)_24_: ^1^H NMR (500 MHz, CD_3_NO_2_, 300 K), δ10.96 (s, 1H, Py(C)N*H*), 10.10 (s, 1H, xN*H*), 9.23 (d, 8 Hz, 1H, GlnN_α_*H*), 9.00 (d, 8.5 Hz, 1H, Py(C)*H*_4′_), 8.84 (s, 1H, Py(N)*H*_2′_), 8.63 (d, 5 Hz, 1H, Py(N)*H*_6′_), 8.42 (d, 8 Hz, 1H, x*H*_4′_), 8.37 (s, 2 Hz, 1H, Py(C)*H*_2′_), 8.22–8.19 (m, 2H, Py(N)*H*_4′_, Py(C)*H*_6′_), 8.02 (d, 7.5 Hz, 1H, x*H*_6′_), 7.98 (s, 1H, x*H*_2′_), 7.74 (dd, 8.5 Hz, 5 Hz, 1H, Py(N)*H*_5′_), 7.59 (dd, 8 Hz, 5.5 Hz, 1H, Py(C)*H*_5′_), 7.22 (t, 8 Hz, 1H, x*H*_5′_), 6.54 (s, 1H, GlnN_δ_*H*), 5.66 (s, 1H, GlnN_δ_*H*′), 5.49 (m, 1H, GlnN*H*_α_), 5.05 (t, 7 Hz, 1H, Pro(1)*H*_α_), 4.92 (m, 1H, Pro(2)*H*_α_), 4.85 (m, 1H, Pro(3)*H*_α_), 4.4, 4.09, 4.02, 3.93 (4H, Pro*H*_δ_), 3.64–3.59 (m, 2H, Pro(1)*H*_δ_), 2.56, 2.4–1.9 (m, 16H, Pro*H*_β, γ_, Gln*H*_β, γ_).

Chemical shifts data of [Ag_24_(**5D**)_24_]•(PF_6_)_24_: ^1^H NMR (500 MHz, CD_3_NO_2_, 300 K), δ11.00 (s, 1H, Py(C)N*H*), 10.06 (s, 1H, xN*H*), 9.43 (d, 8 Hz, 1H, AspN*H*), 8.96 (d, 8.5 Hz, 1H, Py(C)*H*_4′_), 8.67 (s, 1H, Py(N)*H*_2′_), 8.65 (d, 5 Hz, 1H, Py(N)*H*_6′_), 8.45 (d, 1 Hz, 9 Hz, 1H, x*H*_4′_), 8.35 (s, 2 Hz, 1H, Py(C)*H*_2′_), 8.21–8.19 (m, 2H, Py(N)*H*_4′_, Py(C)*H*_6′_), 7.99 (d, 8 Hz, 1H, x*H*_6′_), 7.92 (s, 1H, x*H*_2′_), 7.77 (dd, 8 Hz, 5 Hz, 1H, Py(N)*H*_5′_), 7.57 (dd, 8.5 Hz, 5 Hz, 1H, Py(C)*H*_5′_), 7.33–7.30 (m, 5H, Ph), 7.26 (t, 8 Hz, 1H, x*H*_5′_), 5.80 (m, 1H, AspN*H*_α_), 5.12, 4.69 (abq, 12 Hz, 2H, C*H*_2_Ph), 5.03 (t, 7 Hz, 1H, Pro(1)*H*_α_), 4.96 (m, 1H, Pro(2)*H*_α_), 4.82 (m, 1H, Pro(3)*H*_α_), 4.4, 4.15, 4.09, 3.91 (4H, Pro*H*_δ_), 3.55–3.54 (m, 2H, Pro(1)*H*_δ_), 3.18, 2.94 (m, Asp*H*_β_), 2.58, 2.4–1.8 (m, 12H, Pro*H*_β, γ_).

Chemical shifts data of [Ag_24_(**5K**)_24_]•(Tf_2_N)_24_: ^1^H NMR (500 MHz, CD_3_NO_2_, 300 K), δ10.95 (s, 1H, Py(C)N*H*), 10.21 (s, 1H, xN*H*), 9.09 (d, 8 Hz, 1H, LysN_α_*H*), 8.96 (d, 9 Hz, 1H, Py(C)*H*_4′_), 8.74 (s, 1H, Py(N)*H*_2′_), 8.60 (dd, 1 Hz, 5 Hz, 1H, Py(N)*H*_6′_), 8.36 (d, 8.5 Hz, 1H, x*H*_4′_), 8.23 (d, 8 Hz, 1H, Py(N)*H*_4′_), 8.14–8.13 (m, 2H, Py(C)*H*_6′_, x*H*_6′_) 8.05 (s, 2 Hz, 1H, Py(C)*H*_2′_), 7.97 (s, 1H, x*H*_2′_), 7.75 (dd, 8 Hz, 5.5 Hz, 1H, Py(N)*H*_5′_), 7.53 (dd, 8.5 Hz, 5 Hz, 1H, Py(C)*H*_5′_), 7.4–7.24 (m, 5H, Ph), 7.21 (t, 8 Hz, 1H, x*H*_5′_), 5.80 (m, 1H, LysN_ε_*H*), 5.49 (m, 1H, LysN*H*_α_), 5.04–4.84 (m, 5H, Pro(1)*H*_α_, Pro(2)*H*_α_, Pro(3)*H*_α_, C*H*_2_Ph), 4.48, 4.13, 3.97–3.88 (4H, Pro*H*_δ_), 3.70–3.60 (m, 2H, Pro(1)*H*_δ_), 2.97, 2.87 (m, 2H, Lys*H*_ε_), 2.55, 2.4–1.8 (m, 14H, Pro*H*_β, γ_, Lys*H*_β_), 1.5–1.4 (m, 4H, Lys*H*_γ, δ_).

Chemical shifts data of [Ag_24_(**9**)_24_]•(Tf_2_N)_24_: ^1^H NMR (500 MHz, CD_3_NO_2_, 300 K), δ10.93 (s, 1H, Py(C)N*H*), 10.17 (s, 1H, xN*H*), 9.18 (d, 8 Hz, 1H, CitN_α_*H*), 8.98 (d, 8.5 Hz, 1H, Py(C)*H*_4′_), 8.77 (s, 1H, Py(N)*H*_2′_), 8.68 (d, 5.5 Hz, 1H, Py(N)*H*_6′_), 8.40 (d, 8 Hz, 1H, x*H*_4′_), 8.27 (d, 8 Hz, 1H, Py(N)*H*_4′_), 8.21 (s, 1H, Py(C)*H*_2′_), 8.19 (d, 5 Hz, 1H, Py(C)*H*_6′_), 8.03 (d, 7.5 Hz, 1H, x*H*_6′_), 7.95 (s, 1H, x*H*_2′_), 7.81 (dd, 8 Hz, 5.5 Hz, 1H, Py(N)*H*_5′_), 7.60 (dd, 8.5 Hz, 5.5 Hz, 1H, Py(C)*H*_5′_), 7.25 (t, 8 Hz, 1H, x*H*_5′_), 5.80 (m, 1H, CitN_δ_*H*), 5.42 (m, 1H, CitN*H*_α_), 5.07 (m, 1H, Pro(1)*H*_α_), 4.98 (m, 1H, Pro(2)*H*_α_), 4.83 (m, 1H, Pro(3)*H*_α_), 4.56 (s, 2H, CitN*H*_2_), 4.48, 4.09, 4.02–3.93 (4H, Pro*H*_δ_), 3.68–3.58 (m, 2H, Pro(1)*H*_δ_), 3.10, 2.85 (m, 2H, Cit*H*_δ_), 2.60, 2.4–2.0 (m, 12H, Pro*H*_β, γ_), 1.87, 1.75 (m, 2H, Cit*H*_β_), 1.57, 1.46 (m, 2H, Cit*H*_γ_).

### Crystallographic analysis

Crystallisation was carried out as follows: Two solutions of ligand **5** (15.0 mg, 22 µmol) in CD_3_NO_2_ (500 µL) and AgTf_2_N (8.5 mg, 22 µmol) in CD_3_NO_2_ (50 µL) were mixed in a test tube, and the mixture was stirred at 60 °C for 10 min. 150 µL of the obtained pale brown solution was taken in a microtube (*φ* = 6 mm). After diisopropyl ether vapour was slowly diffused into the microtube over 10 days at room temperature, colourless block crystals were obtained (Supplementary Fig. 33). Although two types of crystal morphologies were confirmed in the same batch, both structures refined in the *I*432 space group (crystal structure A) and the *I*2 space group (crystal structure B) were revealed as the same [6]catenane framework topology. Crystal structure A: Crystallographic diffraction data of **6** refined in the *I*432 space group were measured on a Bruker APEX-II/CCD diffractometer equipped with a focusing mirror (Mo K*α* radiation *λ* = 0.71073 Å) with a cryostat system equipped with a N_2_ generator (Japan Thermal Eng.). The crystals were removed from the solution, quickly attached to a loop of nylon fibre with the antifreeze reagent (parabar 10312, Hampton research), and mounted on a goniometer. The data collection was performed at 93 K. The structures were solved by direct methods (SHELXS-2013)^32^. Crystal structure B: Crystallographic diffraction data of **6** refined in *I*2 space group were measured at the BL38B1 beamline at SPring-8 (Rayonix MX225H&E CCD detector). The crystals were transferred into a glass capillary and the mother liquor was roughly sucked out, which was mounted on a goniometer. The data collection was performed at room temperature (293 K) (radiation *λ* = 1.00 Å). The data reduction was performed with CrysAlis^Pro^ (2015, RIGAKU Oxford Diffraction). The structure was solved by dual space methods (SHELXT-2014)^33^. Both structures were refined by full-matrix least-squares calculations (SHELXL-2014)^34^ on *F*^2^. Hydrogen atoms were fixed at calculated positions and refined using a riding model. The thermal temperature factors except for Ag atoms were isotropically refined. Restraints were applied on the basis of the chemical geometries. Note that the Flack parameter of crystal structure B resulted in invalid values due to the data collection method at the synchrotron (phi-scan, oscillation range: 180°, oscillation angle: 1°). Graphics were generated with PyMOL 2.0. Detailed procedures and data on crystallographic diffraction study are shown in Supplementary Note 1, Supplementary Tables 1–2, and Supplementary Figs. 34–41.

### Dynamic light scattering study

Nitromethane solution of [6]catenane **6**•(PF_6_)_24_, [Ag_24_(**5L**)_24_](OTf)_24_, [Ag_24_(**5Q**)_24_](OTf)_24_, [Ag_24_(**5D**)_24_](PF_6_)_24_, or [Ag_24_(**5K**)_24_](OTf)_24_ (0.42 mM) was five times diluted with nitromethane and passed through a nylon membrane disc filter (pore size: 0.2 µm). Obtained data were shown in Supplementary Fig. 42. In Supplementary Fig. 42a, c–f, hydrodynamic radius *r* was consistent with the radius of the [6]catenane framework of the crystal structure by considering the adjacent counter anions and solvents. In Supplementary Fig. 42b, the solution after complexation with **5G** and AgOTf showed much smaller *r* value than that of the [6]catenane, which is consistent with ^1^H NMR observation (Supplementary Fig. 27g). Some aggregation species were reflected in the distribution of *r* = 40–80 nm.

### Ion mobility mass spectrometry measurements

Nitromethane solution of [6]catenane [Ag_24_(**5L**)_24_](PF_6_)_24_ (0.42 mM) was passed through a nylon membrane disc filter (pore size: 0.2 µm). ESI-TOF-MS measurements assisted by trapped ion-mobility separation (TIMS) were then performed. Obtained data were shown in Supplementary Figs. 43–44 and Supplementary Tables 3–4. Measurement condition: dry gas 8 L•min^–1^, dry temperature 250 °C, Funnel 1 RF 300 Vpp, Funnel 2 RF 400 Vpp, isCID energy 0 eV, multipole RF 1200 Vpp, deflection delta –50 V, quadruple ion energy 7 eV, low mass *m*/*z* = 500, collision energy 15 eV, collision RF 4000 Vpp, transfer time 200 µs, pre pulse storage 10 µs, flow rate 140 µL•h^–1^.

### Calculation of cavity volume

Cavity volumes of natural or de novo designed proteins reported in the Protein Data Bank (PDB) were studied. First, X-ray crystal or solution structures of proteins were searched with the keyword “cavity” on the website https://www.rcsb.org/ (756 hits as of May. 7th, 2019). The search results were then narrowed down by filling the search blanks with chain length [100–200] (479 structures, 117 citations). From the search results, over 30 PDB files were randomly extracted (Supplementary Table 6), and each cavity volume was calculated by using 3 V webserver^19^ with different probe radii depending on the cavity burial (outer probe radii of 6.0–8.0 Å and inner probe radii of 1.0–1.5 Å) and the grid size of 0.5 Å. As for larger proteins, the search results were narrowed down by the input of the chain length [300–600] (229 structures, 81 citations) and 16 PDB files were extracted (Supplementary Table 7). Graphical representations of calculated protein cavities were listed in Supplementary Figs. 56–57.

## Supplementary information

Supplementary Information

Peer Review File

## Data Availability

The authors declare that the data supporting the findings of this study are available within the Supplementary Information files and from the corresponding authors upon reasonable request. The X-ray crystallographic coordinates for structures reported in this study have been deposited at the Cambridge Crystallographic Data Centre (CCDC) under deposition numbers 1881307 and 1881308. These data can be obtained free of charge from the CCDC via http://www.ccdc.cam.ac.uk
