# Supplementary material for: A metal–peptide capsule by multiple ring threading
Source: Nat Commun. 2019 Dec 12;10:5687. doi: 10.1038/s41467-019-13594-4 (PMC6908709; doi:10.1038/s41467-019-13594-4)
Supplement: Supplementary file 1 — Supplementary Information [file 41467_2019_13594_MOESM1_ESM.pdf]

Supplementary Information

A metal–peptide capsule by multiple ring threading

Sawada et al.

Supplementary Methods

Abbreviations

Boc: *tert*-butoxycarbonyl-
Cbz: benzyloxycarbonyl-
OBzl: *O*-benzyl-
DMF: *N*,*N*-dimethylformamide
Pro: L-proline
Ala: L-alanine
Leu: L-leucine
Gln: L-glutamine
Lys: L-lysine
Gly: glycine
Asp: L-aspartate
Cit: L-citrulline
-x-: imino-(1,3-phenylene)carbonyl-

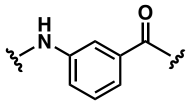

Pentapeptide ligands **5**, **5L**, **5Q**, **5K**, **5G**, and **5P** were synthesised according to the following procedure.

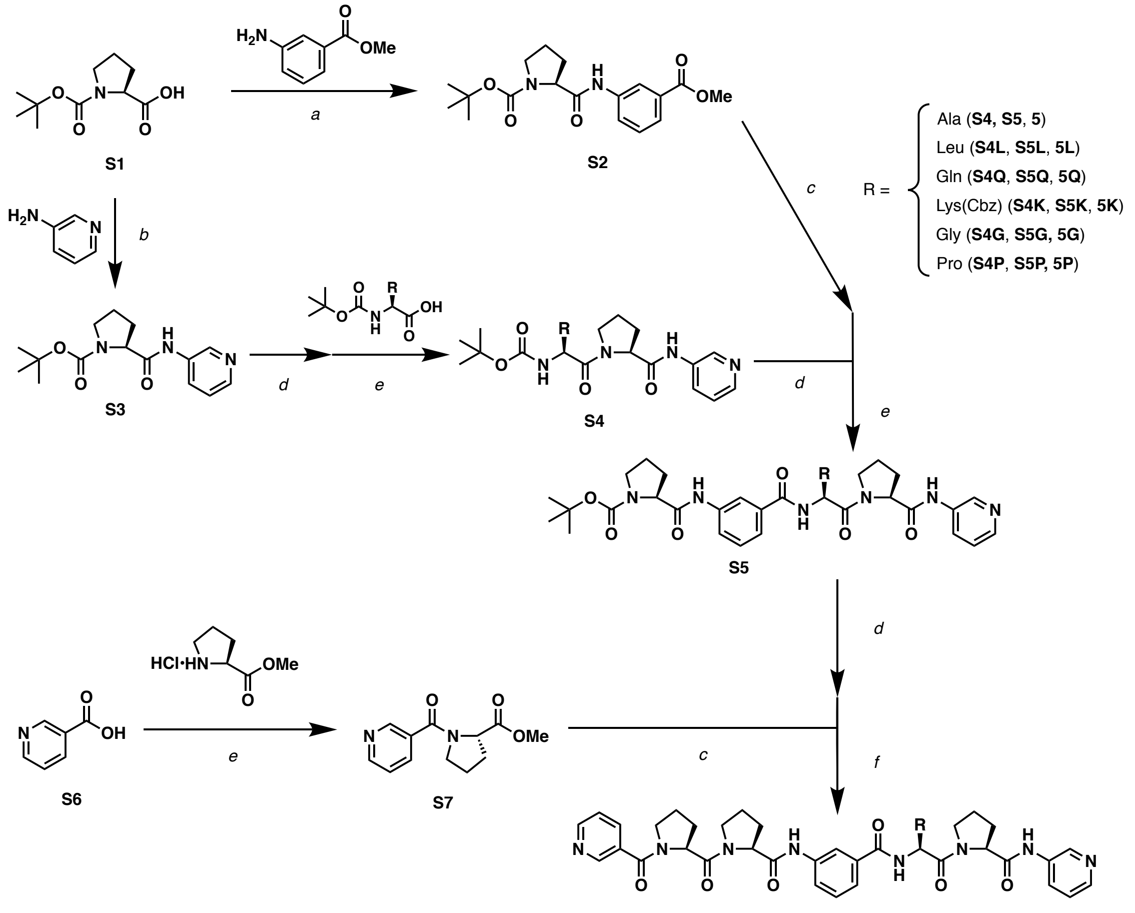

5

Reagents and conditions: (a) NMM, IBCF, in THF, 0 ºC, 20 min, then RT, o/n, (b) DIEA, ECF, in THF, 0 ºC, 20 min, then RT, o/n, (c) LiOH, in MeOH/H_2O, 2 h, (d) HCl, in MeOH/1,4-dioxane, 30 min, (e) HOBt/EDCI, DIEA, in $CHCl_3$, o/n. (f) HOBt/EDCI, DIEA, in DMF, o/n.

Methyl 3-(*N*-Boc-L-prolylimino)-benzoate[1] **(S2): S2** was synthesised by condensation of Boc-Pro-OH and methyl 3-aminobenzoate by using *N*-methylmorpholine (NMM) and isobutyl chloroformate (IBCF). The crude product was purified by SiO_2 column chromatography.

***N*-(3-Pyridyl)-*N'*-Boc-L-prolinamide**[2] **(S3): S3** was synthesised by condensation of Boc-Pro-OH and 3-aminopyridine by using DIEA and ethyl chloroformate (ECF). The crude product was recrystallised from EtOAc.

***N*-(3-Pyridyl)-*N'*-(Boc-L-alanyl)-L-prolinamide (S4):** The removal of the Boc group of **S3** was carried out according to procedure *d*. To the $CHCl_3$ solution of Boc-free **S3** (2.7 g, 10 mmol), Boc-Ala-OH (2.0 g, 10 mmol), HOBt (1.9 g, 12 mmol), EDCI (2.4 g, 12 mmol), and DIEA (6.3 mL, 36 mmol) were sequentially added. The mixture was stirred at room temperature for overnight. After washed with sat. $NaHCO_3$ aq. (×3), the organic layer was dried over $MgSO_4$ and concentrated by evaporation. The crude product was purified by SiO_2 column chromatography ($CHCl_3$/MeOH). The white solid of **S4** 2.4 g was obtained (Y 64%).

M.p. 77–81 ºC; HR-MS: calcd. for $[M+H]^+$: 363.2027, found: 363.2035 (error 2.2 ppm); ^{1}H NMR (500 MHz, $CDCl_3$, 300 K, TMS, one conformer coexists), δ9.93 (brs, 1H, PyN*H*), 8.53 (d, 2 Hz, 1H, Py$H_{2'}$), 8.18 (d, 5 Hz, 1H, Py$H_{6'}$), 7.93 (d, 8 Hz, 1H, Py$H_{4'}$), 7.07 (dd, 5 Hz, 8 Hz, 1H, Py$H_{5'}$), 5.58 (d, 8 Hz, 1H, AlaN*H*), 4.77 (m, 1H, ProH_α), 4.54 (m, 1H, AlaH_α), 3.78, 3,67 (m, 2H, ProH_δ), 2.5-2.0 (m, 4H, ProH_γ, Pro$H_{\beta'}$), 1.44 (brs, 9H, *t*-Bu), 1.40 (t, 7 Hz, 3H, AlaH_β); ^{13}C NMR (125 MHz, $CDCl_3$, 300 K, TMS, one conformer coexists), δ173.2 (Ala*C*O), 170.3 (Pro*C*O), 155.3 (Boc*C*O), 144.4 (Py$C_{6'}$), 140.9 (Py$C_{2'}$), 135.4 (Py$C_{3'}$), 126.5 (Py$C_{4'}$), 123.4 (Py$C_{5'}$), 79.8 (BocC_q), 60.7 (ProC_α), 48.0 (AlaC_α), 47.5 (ProC_δ), 28.5 (BocCH_3), 28.2 (ProC_β), 25.1 (ProC_γ), 18.1 (AlaC_β).

***N*-(3-Pyridyl)-*N*^{1,1}-Boc-L-prolylimino-(1,3-phenylene)carbonyl-L-alanyl-L-prolinamide (S5):**
S2 (6.8 g, 20 mmol) was dissolved in MeOH/H_2O (30/15 mL) and LiOH•H_2O (4.1 g, 98 mmol) was added. After stirring at room temperature for 2 h, the mixture was neutralized with 5 N HCl aq. and then MeOH was removed by evaporation. To the solution, 5% citric acid aq. (10 mL) was added and extracted with $CHCl_3$. The organic layer was dried over $MgSO_4$ and the solvents were evaporated. Quantitative hydrolysis of the COOMe group was confirmed by ^{1}H NMR measurement. The removal of the Boc group of **S4** was carried out according to procedure *d*.

The hydrolyzed **S2** (1.1 g, 3.3 mmol) was dissolved in $CHCl_3$ (20 mL). To the solution, Boc-free **S4** (1.1 g, 3.3 mmol), HOBt (0.60 g, 3.9 mmol), EDCI (0.75 g, 3.9 mmol), and DIEA (2.0 mL, 11 mmol) were sequentially added. After overnight reaction, the mixture was washed with sat. $NaHCO_3$ aq. (×2). The organic layer was dried over $MgSO_4$ and concentrated by evaporation. The crude product was purified by SiO_2 column chromatography ($CHCl_3$/MeOH). The white solid of **S5** 1.7 g was obtained (Y 87%).

M.p. 128–133 ºC; HR-MS: calcd. for $[M+H]^+$: 579.2926, found: 579.2927 (error 0.2 ppm); ^{1}H NMR (500 MHz, DMSO-d_6, 300 K, one conformer coexists), δ10.21 (s, 1H, PyN*H*), 10.16 (s, 1H, xN*H*), 8.75 (brs, 1H, Py$H_{2'}$), 8.56 (m, 1H, AlaN*H*), 8.26 (d, 4 Hz, 1H, Py$H_{6'}$), 8.08–8.04 (m, 2H, x$H_{2'}$, Py$H_{4'}$), 7.83 (d, 8 Hz, 1H, x$H_{4'}$), 7.58 (m, 1H, x$H_{6'}$), 7.40 (m, 1H, x$H_{5'}$), 7.33 (m, 1H, Py$H_{5'}$), 4.77 (m, 1H, AlaH_α), 4.49 (m, 1H, Pro(2)H_α), 4.21 (m, 1H, Pro(1)H_α), 3.78, 3.67 (m, 2H, Pro(2)H_δ), 3.43, 3.34 (m, 2H, Pro(1)H_δ), 2.2 (m, 2H, Pro(1)H_β, Pro(2)H_β), 2.04 (m, 1H, Pro(2)H_γ), 2.0–1.8 (m, 5H, Pro(1)$H_{\beta',\gamma}$, Pro(2)$H_{\beta',\gamma'}$), 1.40, 1.27 (m, 9H, *t*-Bu), 1.36 (m 3H, AlaH_β); ^{13}C NMR (125 MHz, DMSO-d_6, 300 K, one conformer coexists), δ172.1 (Pro(1)*C*O), 171.5 (Pro(2)*C*O), 171.3 (Ala*C*O), 166.4 (x*C*O), 153.6 (Boc*C*O), 144.5 (Py$C_{6'}$), 141.2 (Py$C_{2'}$), 139.6, 135.1 (x$C_{1'}$, x$C_{3'}$), 136.2 (Py$C_{3'}$), 129.0 (x$C_{5'}$), 126.5 (Py$C_{4'}$), 124.0 (Py$C_{5'}$), 122.4 (x$C_{4'}$, x$C_{6'}$), 119.2 (x$C_{2'}$), 80.0 (BocC_q), 60.9 (Pro(1)C_α), 60.8 (Pro(2)C_α), 47.6 (AlaC_α), 47.3 (Pro(2)C_δ), 47.0 (Pro(1)C_δ), 31.5 (Pro(1)C_β), 29.7 (Pro(2)C_β), 28.4 (BocCH_3), 25.2 (Pro(2)C_γ), 23.8 (Pro(1)C_γ), 17.0 (AlaC_β).

N-Nicotinoyl-L-proline methyl ester[3] (S7): **S7** was synthesised by condensation of nicotinic acid and H-Pro-OMe hydrochloride by using EDCI/HOBt and DIEA (procedure *e*). The crude product was purified by SiO_2 column chromatography.

Pentapeptide ligand 5: The hydrolysis of the COOMe group of **S7** was carried out according to procedure *c*. The removal of the Boc group of **S5** was carried out according to procedure *d*. The hydrolyzed **S7** (0.58 g, 2.6 mmol), Boc-free **S5** (1.5 g, 2.6 mmol), HOBt (0.48 g, 3.2 mmol), EDCI (0.61 g, 3.2 mmol), and DIEA (1.8 mL, 11 mmol) were added in DMF (50 mL) and the mixture was stirred at room temperature. After 2 d, the solvents were removed by evaporation. The crude product was purified by SiO_2 column chromatography (EtOAc/MeOH), and then further purified by using recycle SEC (eluent: MeOH/H_2O). The white powder of **5** 0.78 g was obtained (Y 44%).

M.p. 147–152 ºC; E. A.: calcd. for $C_{36}H_{40}N_8O_6$•2.5(H_2O): C, 59.57; H, 6.25; N, 15.44%; found: C, 59.33; H, 6.12; N, 15.37%; HR-MS: calcd. for $[M+H]^+$: 681.3144, found: 681.3176 (error: 4.7 ppm); ^{1}H NMR (500 MHz, DMSO-d_6, 300 K, two conformers coexist), δ10.21 (s, 1H, Py(C)N*H*), 10.18 (s, 1H, xN*H*), 8.74 (d, 2 Hz, 1H, Py(C)$H_{2'}$), 8.69 (brs, 1H, Py(N)$H_{2'}$), 8.67 (dd, 1 Hz, 5 Hz, 1H, Py(N)$H_{6'}$), 8.56 (m, 1H, AlaN*H*), 8.26 (d, 5 Hz, 1H, Py(C)$H_{6'}$), 8.07–8.03 (m, 2H, Py(C)$H_{4'}$, x$H_{2'}$), 7.90 (d, 8 Hz, 1H, Py(N)$H_{4'}$), 7.72 (d, 8 Hz, 1H, x$H_{4'}$), 7.56 (m, 1H, x$H_{6'}$), 7,48 (dd, 5 Hz, 8 Hz, 1H, Py(N)$H_{5'}$), 7.38 (t, 8 Hz, 1H, x$H_{5'}$), 7.34 (dd, 5 Hz, 8 Hz, 1H, Py(C)$H_{5'}$), 4.80 (m, 1H, Pro(1)H_α), 4.75 (m, 1H, AlaH_α), 4.5 (m, 2H, Pro(2)H_α, Pro(3)H_α), 3.77, 3.65 (m, 4H, Pro(2)H_δ, Pro(3)H_δ), 3.51 (m, 2H, Pro(1)H_δ), 2.33 (m, 1H, Pro(1)H_β), 2.2, 2.1–1.8 (m, 11H, Pro(1)$H_{\beta',\gamma}$, Pro(2)$H_{\beta,\gamma}$, Pro(3)$H_{\beta,\gamma}$), 1.34 (m, 3H, AlaH_β); ^{13}C NMR (125 MHz, DMSO-d_6, 300 K, two conformers coexist), δ171.6 (Pro(3)*C*O), 171.3 (Ala*C*O), 171.1 (Pro(2)*C*O), 170.0 (Pro(1)*C*O), 166.5 (x*C*O), 166.1 (Py(N)*C*O), 151.2 (Py(N)$C_{6'}$), 148.2 (Py(N)$C_{2'}$), 144.6 (Py(C)$C_{6'}$), 141.2 (Py(C)$C_{2'}$), 139.6, 135.2 (x$C_{1'}$, x$C_{3'}$), 136.2 (Py(C)$C_{3'}$), 135.1 (Py(N)$C_{4'}$), 132.8 (Py(N)$C_{3'}$), 129.0 (x$C_{5'}$), 126.5 (Py(C)$C_{4'}$), 124.0 (Py(C)$C_{5'}$), 123.9 (Py(N)$C_{5'}$), 122.4, 122.2 (x$C_{6'}$, x$C_{4'}$), 119.0 (x$C_{2'}$), 60.7, 60.6 (Pro(2)C_α, Pro(3)C_α), 58.6 (Pro(1)C_α), 50.1 (Pro(1)C_δ), 47.6 (AlaC_α), 47.3, 47.2 (Pro(2)C_δ, Pro(3)C_δ), 29.7 (Pro(2)C_β, Pro(3)C_β), 28.6 (Pro(1)C_β), 25.3–25.1 (ProC_γ), 17.0 (AlaC_β).

N-(3-Pyridyl)-N'-(Boc-L-leucyl)-L-prolinamide (S4L): The removal of the Boc group of **S3** was carried out according to procedure *d*. To the $CHCl_3$ solution of Boc-free **S3** (0.49 g, 1.9 mmol), Boc-Leu-OH•H_2O (0.49 g, 1.9 mmol), HOBt (0.35 g, 2.3 mmol), EDCI (0.44 g, 2.3 mmol), and DIEA (1.2 mL, 6.7 mmol) were sequentially added. The mixture was stirred at room temperature for overnight. After washed with sat. $NaHCO_3$ aq. (×3), the organic layer was dried over $MgSO_4$ and concentrated by evaporation. The crude product was purified by SiO_2 column chromatography ($CHCl_3$/MeOH). The white solid of **S4L** 0.60 g was obtained (Y 78%).

M.p. 129–135 ºC; HR-MS: calcd. for $[M+Na]^+$: 427.2316, found: 427.2335 (error 4.7 ppm); ^{1}H NMR (500 MHz, DMSO-d_6, 300 K, a conformer coexists), δ10.21 (s, 1H, PyN*H*), 8.69 (d, 2 Hz, 1H, Py$H_{2'}$), 8.23 (dd, 2 Hz, 5 Hz, 1H, Py$H_{6'}$), 8.00 (dt, 2 Hz, 8 Hz, 1H, Py$H_{4'}$), 7.31 (dd, 5 Hz, 8 Hz, 1H, Py$H_{5'}$), 6.93 (d, 8 Hz, 1H, LeuN*H*), 4.44 (m, 1H, ProH_α), 4.22 (m, 1H, LeuH_α), 3.69, 3,53 (m, 2H, ProH_δ), 2.15, 1.88 (m, 2H, ProH_β), 2.02, 1.93 (m, 2H, ProH_γ), 1.65 (m, 1H, LeuH_γ), 1.44–1.34 (m, 11H, *t*-Bu, LeuH_β), 0.88 (dd, 5 Hz, 7 Hz, 6H, LeuH_δ); ^{13}C NMR (125 MHz, DMSO-d_6, 300 K), δ171.6, 171.5 (Leu*C*O, Pro*C*O), 155.99 (Boc*C*O), 144.6 (Py$C_{6'}$), 141.1 (Py$C_{2'}$), 136.2 (Py$C_{3'}$), 126.4 (Py$C_{4'}$), 124.0 (Py$C_{5'}$), 78.3 (BocC_q), 60.5 (ProC_α), 50.9 (LeuC_α), 47.1 (ProC_δ), 39.7 (LeuC_β), 29.7 (ProC_β), 28.7 (BocCH_3), 25.2 (ProC_γ), 24.6 (LeuC_γ), 23.7, 21.8 (LeuC_δ).

N-(3-Pyridyl)-$N^{1,1}$-Boc-L-prolylimino-(1,3-phenylene)carbonyl-L-leucyl-L-prolinamide (S5L): The hydrolysis of the COOMe group of **S2** was carried out according to procedure *c*. The removal of the Boc group of **S4L** was carried out according to procedure *d*. The hydrolyzed **S2** (0.40 g, 1.2 mmol) was dissolved in $CHCl_3$ (10 mL). To the solution, Boc-free **S4L** (0.46 g, 1.2 mmol), HOBt (0.22 g, 1.4 mmol), EDCI (0.28 g, 1.4 mmol), and DIEA (0.7 mL, 4.2 mmol) were sequentially added. After overnight reaction, the mixture was washed with sat. $NaHCO_3$ aq. (×2). The organic layer was dried over $MgSO_4$ and concentrated by evaporation. The crude product was purified by SiO_2 column chromatography ($CHCl_3$/MeOH). The white solid of **S5L** 0.64 g was obtained (Y 86%).

M.p. 142–146 ºC; HR-MS: calcd. for $[M+H]^+$: 621.3395, found: 621.3393 (error 0.5 ppm); ^1H NMR (500 MHz, DMSO-d_6, 300 K, one conformer coexists), δ10.25 (s, 1H, PyN*H*), 10.11 (s, 1H, xN*H*), 8.73 (brs, 1H, Py$H_{2'}$), 8.53 (m, 1H, LeuN*H*), 8.26 (d, 4 Hz, 1H, Py$H_{6'}$), 8.04-8.01 (m, 2H, x$H_{2'}$, Py$H_{4'}$), 7.82 (d, 8 Hz, 1H, x$H_{4'}$), 7.58 (m, 1H, x$H_{6'}$), 7.40 (m, 1H, x$H_{5'}$), 7.34 (m, 1H, Py$H_{5'}$), 4.79 (m, 1H, LeuH_α), 4.49 (m, 1H, Pro(2)H_α), 4.21 (m, 1H, Pro(1)H_α), 3.84, 3.65 (m, 2H, Pro(2)H_δ), 3.43, 3.34 (m, 2H, Pro(1)H_δ), 2.2 (m, 2H, Pro(1)H_β, Pro(2)H_β), 2.05 (m, 1H, Pro(2)H_γ), 2.0–1.8 (m, 5H, Pro(1)$H_{\beta',\gamma}$, Pro(2)$H_{\beta',\gamma'}$, LeuH_γ), 1.73, 1.52(m ,2H, LeuH_β), 1.40, 1.27 (m, 9H, *t*-Bu), 0.95 (m, 6H, LeuH_δ); ^{13}C NMR (125 MHz, DMSO-d_6, 300 K, one conformer coexists), δ172.2 (Pro(1)*C*O), 171.5 (Pro(2)*C*O), 171.1 (Leu*C*O), 166.9 (x*C*O), 153.6 (Boc*C*O), 144.6 (Py$C_{6'}$), 141.1 (Py$C_{2'}$), 139.6, 135.3 (x$C_{1'}$, x$C_{3'}$), 136.2 (Py$C_{3'}$), 129.0 (x$C_{5'}$), 126.4 (Py$C_{4'}$), 124.1 (Py$C_{5'}$), 122.4 (x$C_{4'}$, x$C_{6'}$), 119.2 (x$C_{2'}$), 79.0 (BocC_q), 60.9 (Pro(1)C_α), 60.7 (Pro(2)C_α), 50.1 (LeuC_α), 47.3 (Pro(2)C_δ), 47.0 (Pro(1)C_δ), 39.7 (LeuC_β), 31.4 (Pro(1)C_β), 29.7 (Pro(2)C_β), 28.4 (BocCH_3), 25.2 (Pro(2)C_γ), 24.8 (LeuC_γ), 23.8 (Pro(1)C_γ), 23.6, 21.8 (LeuC_δ).

Pentapeptide ligand 5L: The hydrolysis of the COOMe group of **S7** was carried out according to procedure *c*. The removal of the Boc group of **S5L** was carried out according to procedure *d*. The hydrolyzed **S7** (0.18 g, 0.8 mmol), Boc-free **S5L** (0.48 g, 0.8 mmol), HOBt (0.15 g, 1.0 mmol), EDCI (0.19 g, 1.0 mmol), and DIEA (0.6 mL, 3.3 mmol) were added in DMF (10 mL) and the mixture was stirred at room temperature. After 3 d, the solvents were removed by evaporation. The crude product was purified by SiO_2 column chromatography (EtOAc/MeOH), and then further purified by using recycle SEC (eluent: $CHCl_3$). The white powder of **5L** 0.25 g was obtained (Y 42%).

M.p. 172 ºC dec; HR-MS: calcd. for $[M+H]^+$: 723.3613, found: 723.3621 (error: 1.1 ppm); ^1H NMR (500 MHz, DMSO-d_6, 300 K, two conformers coexist), δ10.25 (s, 1H, Py(C)N*H*), 10.18 (s, 1H, xN*H*), 8.73 (d, 2 Hz, 1H, Py(C)$H_{2'}$), 8.69 (d, 1 Hz, 1H, Py(N)$H_{2'}$), 8.66 (dd, 2 Hz, 5 Hz, 1H, Py(N)$H_{6'}$), 8.53 (m, 1H, LeuN*H*), 8.26 (d, 5 Hz, 1H, Py(C)$H_{6'}$), 8.05–8.00 (m, 2H, Py(C)$H_{4'}$, x$H_{2'}$), 7.88 (dt, 8 Hz, 2 Hz, 1H, Py(N)$H_{4'}$), 7.73 (dd, 1 Hz, 8 Hz, 1H, x$H_{4'}$), 7.56 (t, 8 Hz, 1H, x$H_{6'}$), 7.44 (dd, 5 Hz, 8 Hz, 1H, Py(N)$H_{5'}$), 7.4–7.3 (m, 2H, x$H_{5'}$, Py(C)$H_{5'}$), 4.80–4.79 (m, 2H, Pro(1)H_α, LeuH_α), 4.49 (m, 2H, Pro(2)H_α, Pro(3)H_α), 3.82, 3.66–3.53 (m, 4H, Pro(2)H_δ, Pro(3)H_δ), 3.48 (m, 2H, Pro(1)H_δ), 2.32 (m, 1H, Pro(1)H_β), 2.2, 2.1–1.5 (m, 14H, Pro(1)$H_{\beta',\gamma}$, Pro(2)$H_{\beta,\gamma}$, Pro(3)$H_{\beta,\gamma}$, Leu$H_{\beta,\gamma}$), 0.94 (s, 6H, LeuH_δ); ^{13}C NMR (125 MHz, DMSO-d_6, 300 K, two conformers coexist), δ171.5 (Pro(3)*C*O), 171.2, 171.1 (Pro(2)*C*O, Leu*C*O), 170.0 (Pro(1)*C*O), 166.9 (x*C*O), 166.1 (Py(N)*C*O), 151.2 (Py(N)$C_{6'}$), 148.2 (Py(N)$C_{2'}$), 144.6 (Py(C)$C_{6'}$), 141.1 (Py(C)$C_{2'}$), 139.6, 135.3 (x$C_{1'}$, x$C_{3'}$), 136.2 (Py(C)$C_{3'}$), 135.1 (Py(N)$C_{4'}$), 132.8 (Py(N)$C_{3'}$), 129.0 (x$C_{5'}$), 126.4 (Py(C)$C_{4'}$), 124.1 (Py(C)$C_{5'}$), 123.9 (Py(N)$C_{5'}$), 122.4, 122.2 (x$C_{6'}$, x$C_{4'}$), 119.0 (x$C_{2'}$), 60.7, 60.6 (Pro(2)C_α, Pro(3)C_α), 58.6 (Pro(1)C_α), 50.1 (Pro(1)C_δ, LeuC_α), 47.3, 47.2 (Pro(2)C_δ, Pro(3)C_δ), 39.5 (LeuC_β), 29.7 (Pro(2)C_β, Pro(3)C_β), 28.7 (Pro(1)C_β), 25.3–25.2, 24.8 (ProC_γ, LeuC_γ), 23.7, 21.8 (LeuC_δ).

***N*-(3-Pyridyl)-*N'*-Boc-L-glutamylprolinamide (S4Q):** The removal of the Boc group of **S3** was carried out according to procedure *d*. To the $CHCl_3$ (~10 mL) solution of Boc-free **S3** (0.82 g, 3.1 mmol), Boc-Gln-OH (0.79 g, 3.1 mmol), HOBt (0.57 g, 3.7 mmol), EDCI (0.70 g, 3.7 mmol), and DIEA (1.9 mL, 11 mmol) were sequentially added. The mixture was stirred at room temperature for 4 d. After washed with sat. $NaHCO_3$ aq. (×2), the organic layer was dried over $MgSO_4$ and concentrated by evaporation. The crude product was reprecipitated by EtOAc/MeOH. The white solid of **S4Q** 0.53 g was obtained (Y 40%).

M.p. 115–120 ºC; HR-MS: calcd. for $[M+H]^+$: 420.2241, found: 420.2250 (error 2.1 ppm); ^1H NMR (500 MHz, DMSO-d_6, 300 K, one conformer coexists), δ10.25 (s, 1H, PyN*H*), 8.72 (d, 2 Hz, 1H, Py$H_{2'}$), 8.26 (d, 4 Hz, 1H, Py$H_{6'}$), 8.02 (d, 8 Hz, 1H, Py$H_{4'}$), 7.33 (m, 1H, Py$H_{5'}$), 7.27, 6.78 (s, 2H, GlnNH_2), 6.98 (d, 8 Hz, 1H, Gln$N_\alpha H$), 4.46 (m, 1H, ProH_α), 4.20 (m, 1H, GlnH_α), 3.68 (m, 2H, ProH_δ), 2.2–1.9, 1.63 (m, 8H, Pro$H_{\beta,\gamma}$, Gln$H_{\beta,\gamma}$), 1.37 (s, 9H, *t*-Bu); ^{13}C NMR (125 MHz, DMSO-d_6, 300 K, one conformer coexists), δ174.3 (Gln*C*ONH_2), 171.6 (Pro*C*O), 171.1 (Gln*C*O), 155.9 (Boc*C*O), 144.6 (Py$C_{6'}$), 141.1 (Py$C_{2'}$), 136.2 (Py$C_{3'}$), 126.4 (Py$C_{4'}$), 124.1 (Py$C_{5'}$), 78.4 (BocC_q), 60.5 (ProC_α), 52.0 (GlnC_α), 47.2 (ProC_δ), 31.3 (GlnC_γ), 29.7 (ProC_β), 28.7 (BocCH_3), 26.9 (GlnC_β), 25.2 (ProC_γ).

***N*-(3-Pyridyl)-$N^{1,1}$-Boc-L-prolylimino-(1,3-phenylene)carbonyl-L-glutamyl-L-prolinamide (S5Q):** The hydrolysis of the COOMe group of **S2** was carried out according to procedure *c*. The removal of the Boc group of **S4Q** was carried out according to procedure *d*. The hydrolyzed **S2** (0.40 g, 1.2 mmol) was dissolved in $CHCl_3$ (10 mL). To the solution, Boc-free **S4Q** (0.47 g, 1.2 mmol), HOBt (0.22 g, 1.4 mmol), EDCI (0.28 g, 1.4 mmol), and DIEA (0.8 mL, 4.2 mmol) were sequentially added. After stirred for 4 d, the mixture was washed with sat. $NaHCO_3$ aq. once. The organic layer was dried over $MgSO_4$ and concentrated by evaporation. The crude product was purified by reprecipitation from EtOAc. The white solid of **S5Q** 0.48 g was obtained (Y 63%).

M.p. 132–137 ºC; HR-MS: calcd. for $[M+H]^+$: 636.3140, found: 636.3162 (error 3.5 ppm); ^{1}H NMR (500 MHz, DMSO-d_6, 300 K, one conformer coexists), δ10.28 (s, 1H, PyN*H*), 10.13 (s, 1H, xN*H*), 8.73 (d, 2 Hz, 1H, Py$H_{2'}$), 8.62 (d, 7 Hz, 1H, GlnN$_\alpha$*H*), 8.26 (d, 4 Hz, 1H, Py$H_{6'}$), 8.05, 8.03 (m, 2H, x$H_{2'}$, Py$H_{4'}$), 7.81 (d, 8 Hz, 1H, x$H_{4'}$), 7.59 (m, 1H, x$H_{6'}$), 7.39 (m, 1H, x$H_{5'}$), 7.35–7.33 (m, 2H, Py$H_{5'}$, GlnNH_2), 6.84 (s, GlnN$H_{2'}$), 4.69 (m, 1H, GlnH_α), 4.48 (m, 1H, Pro(2)H_α), 4.21 (m, 1H, Pro(1)H_α), 3.78 (m, 2H, Pro(2)H_δ), 3.4–3.3 (m, 2H, Pro(1)H_δ), 2.3–1.8 (m, 12H, Pro$H_{\beta,\gamma}$, Gln$H_{\beta,\gamma}$), 1.40, 1.27 (m, 9H, *t*-Bu); ^{13}C NMR (125 MHz, DMSO-d_6, 300 K, one conformer coexists), δ174.5 (Gln*C*ONH_2), 172.2 (Pro(1)*C*O), 171.6 (Pro(2)*C*O), 170.7 (Gln*C*O), 166.8 (x*C*O), 153.6 (Boc*C*O), 144.6 (Py$C_{6'}$), 141.1 (Py$C_{2'}$), 139.6, 135.1 (x$C_{1'}$, x$C_{3'}$), 136.2 (Py$C_{3'}$), 129.0 (x$C_{5'}$), 126.5 (Py$C_{4'}$), 124.1 (Py$C_{5'}$), 122.4 (x$C_{4'}$, x$C_{6'}$), 119.3 (x$C_{2'}$), 79.0 (BocC_q), 60.8 (Pro(1)C_α), 60.6 (Pro(2)C_α), 51.5 (GlnC_α), 47.3 (Pro(2)C_δ), 47.0 (Pro(1)C_δ), 31.5 (GlnC_γ), 29.8 (Pro(2)C_β, Pro(3)C_β), 28.4 (BocCH_3), 26.7 (GlnC_β), 25.2 (Pro(2)C_γ), 23.8 (Pro(1)C_γ).

Pentapeptide ligand 5Q: The hydrolysis of the COOMe group of **S7** was carried out according to procedure *c*. The removal of the Boc group of **S5Q** was carried out according to procedure *d*. The hydrolyzed **S7** (0.18 g, 0.8 mmol), Boc-free **S5Q** (0.46 g, 0.8 mmol), HOBt (0.15 g, 1.0 mmol), EDCI (0.19 g, 1.0 mmol), and DIEA (0.7 mL, 3.7 mmol) were added in DMF (10 mL) and the mixture was stirred at 40 ºC. After overnight reaction, the solvents were removed by evaporation. The crude product was purified by SiO_2 column chromatography (EtOAc/MeOH), and then further purified by using recycle SEC (eluent: $CHCl_3$). The white powder of **5Q** 0.20 g was obtained (Y 33%).

M.p. 166–171 ºC; HR-MS: calcd. for $[M+H]^+$: 738.3358, found: 738.3349 (error: 1.2 ppm); ^{1}H NMR (500 MHz, DMSO-d_6, 300 K, two conformers coexist), δ10.27 (s, 1H, Py(C)N*H*), 10.19 (s, 1H, xN*H*), 8.74 (d, 2 Hz, 1H, Py(C)$H_{2'}$), 8.68 (brs, 1H, Py(N)$H_{2'}$), 8.66 (d, 5 Hz, 1H, Py(N)$H_{6'}$), 8.62 (m, 1H, GlnN$_\alpha$*H*), 8.26 (d, 5 Hz, 1H, Py(C)$H_{6'}$), 8.07–8.02 (m, 2H, Py(C)$H_{4'}$, x$H_{2'}$), 7.90 (d, 8 Hz, 1H, Py(N)$H_{4'}$), 7.72 (d, 8 Hz, 1H, x$H_{4'}$), 7.57 (t, 8 Hz, 1H, x$H_{6'}$), 7.48 (dd, 5 Hz, 8 Hz, 1H, Py(N)$H_{5'}$), 7.39 (t, 8 Hz, 1H, x$H_{5'}$), 7.35 (m, 1H, Py(C)$H_{5'}$), 7.33, 6.84 (s, 2H, GlnNH_2), 4.80 (m, 1H, Pro(1)H_α), 4.70 (m, 1H, GlnH_α), 4.50–4.48 (m, 2H, Pro(2)H_α, Pro(3)H_α), 3.79, 3.66–3.53 (m, 4H, Pro(2)H_δ, Pro(3)H_δ), 3.49 (m, 2H, Pro(1)H_δ), 2.33 (m, 1H, Pro(1)H_β), 2.2, 2.1–1.7 (m, 15H, Pro(1)$H_{\beta',\gamma}$, Pro(2)$H_{\beta,\gamma}$, Pro(3)$H_{\beta,\gamma}$, Gln$H_{\beta,\gamma}$); ^{13}C NMR (125 MHz, DMSO-d_6, 300 K, two conformers coexist), δ174.5 (GlnC_δO), 171.6 (Pro(3)*C*O), 171.1, 170.7 (Pro(2)*C*O, Gln*C*O), 170.0 (Pro(1)*C*O), 166.8 (X*C*O), 166.1 (Py(N)*C*O), 151.2 (Py(N)$C_{6'}$), 148.2 (Py(N)$C_{2'}$), 144.6 (Py(C)$C_{6'}$), 141.1 (Py(C)$C_{2'}$), 139.6, 135.2 (x$C_{1'}$, x$C_{3'}$), 136.2 (Py(C)$C_{3'}$), 135.2 (Py(N)$C_{4'}$), 132.8 (Py(N)$C_{3'}$), 129.0 (x$C_{5'}$), 126.5 (Py(C)$C_{4'}$), 124.1 (Py(C)$C_{5'}$), 123.9 (Py(N)$C_{5'}$), 122.4, 122.3 (x$C_{6'}$, x$C_{4'}$), 119.0 (x$C_{2'}$), 60.6 (Pro(2)C_α, Pro(3)C_α), 58.6 (Pro(1)C_α), 51.5 (GlnH_α), 50.1 (Pro(1)C_δ), 47.3, 47.2 (Pro(2)C_δ, Pro(3)C_δ), 31.5 (GlnC_γ), 29.8, 29.7 (Pro(2)C_β, Pro(3)C_β), 28.6 (Pro(1)C_β), 26.6 (GlnC_β), 25.3–25.2, 24.8 (ProC_γ).

***N*-(3-Pyridyl)-*N'*-(α-Boc-ε-Cbz-L-lysyl)-L-prolinamide (S4K):** The removal of the Boc group of **S3** was carried out according to procedure *d*. To the $CHCl_3$ solution of Boc-free **S3** (0.82 g, 3.1 mmol), Boc-Lys(Cbz)-OH (1.17 g, 3.1 mmol), HOBt (0.57 g, 3.7 mmol), EDCI (0.71 g, 3.7 mmol), and DIEA (1.9 mL, 11 mmol) were sequentially added. The mixture was stirred at room temperature for overnight. After washed with sat. $NaHCO_3$ aq. (×3), the organic layer was dried over $MgSO_4$ and concentrated by evaporation. The crude product was purified by SiO_2 column chromatography ($CHCl_3$/MeOH). The white solid of **S4K** 0.87 g was obtained (Y 50%).

M.p. 71–93 ºC; HR-MS: calcd. for $[M+H]^+$: 554.2973, found: 554.2990 (error 3.1 ppm); ^{1}H NMR (500 MHz, DMSO-d_6, 300 K, one conformer coexists), δ10.20 (s, 1H, PyN*H*), 8.71 (d, 2 Hz, 1H, Py$H_{2'}$), 8.23

(dd, 2 Hz, 5 Hz, 1H, Py$H_{6'}$), 8.01 (ddd, 2 Hz, 5 Hz, 8 Hz, 1H, Py$H_{4'}$), 7.35–7.30 (m, 5H, Ph), 7.28 (m, 1H, Py$H_{5'}$), 7.21 (m, 1H, Lys$N_{\varepsilon}H$), 6.90 (d, 8 Hz, 1H, Lys$N_{\alpha}H$), 5.00 (s, 1H, OCH_2Ph), 4.44 (m, 1H, ProH_{α}), 4.15 (m, 1H, LysH_{α}), 3.69, 3.53 (m, 2H, ProH_{δ}), 2.98 (brs, 1H, LysH_{ε}), 2.15, 1.88 (m, 2H, ProH_{β}), 1.99, 1.88 (m, 2H, ProH_{γ}), 1.57, 1.48 (m, 2H, LysH_{β}), 1.4 (m, 4H, Lys$H_{\gamma,\delta}$), 1.35 (s, 9H, *t*-Bu); ^{13}C NMR (125 MHz, DMSO-d_6, 300 K, one conformer coexists), δ171.6 (Pro*C*O), 171.3 (Lys*C*O), 156.6 (Cbz*C*O), 156.0 (Boc*C*O), 144.6 (Py$C_{6'}$), 141.1 (Py$C_{2'}$), 137.8 (PhC_i), 136.2 (Py$C_{3'}$), 128.8 (PhC_m), 128.2 (Ph$C_{o,p}$), 126.4 (Py$C_{4'}$), 124.1 (Py$C_{5'}$), 78.4 (BocC_q), 65.6 (OCH$_2$Ph), 60.6 (ProC_{α}), 52.6 (LysC_{α}), 47.2 (ProC_{δ}), 39.9 (LeuC_{ε}), 30.7 (LysC_{β}), 29.7, 29.6 (ProC_{β}, LysC_{δ}), 28.7 (BocCH_3), 25.2 (ProC_{γ}), 23.0 (LysC_{γ}).

***N*-(3-Pyridyl)-*N*[1,1]-Boc-L-prolylimino-(1,3-phenylene)carbonyl-ε-Cbz-L-lysyl-L-prolinamide (S5K):** The hydrolysis of the COOMe group of **S2** was carried out according to procedure *c*. The removal of the Boc group of **S4K** was carried out according to procedure *d*. The hydrolyzed **S2** (0.50 g, 1.5 mmol) was dissolved in $CHCl_3$ (10 mL). To the solution, Boc-free **S4K** (0.81 g, 1.5 mmol), HOBt (0.27 g, 1.8 mmol), EDCI (0.34 g, 1.8 mmol), and DIEA (0.9 mL, 5.2 mmol) were sequentially added. After overnight reaction, the mixture was washed with sat. $NaHCO_3$ aq. (×2). The organic layer was dried over $MgSO_4$ and concentrated by evaporation. The crude product was purified by SiO_2 column chromatography ($CHCl_3$/MeOH). The white solid of **S5K** 0.65 g was obtained (Y 59%).

M.p. 120–125 °C; HR-MS: calcd. for $[M+H]^+$: 770.3872, found: 770.3861 (error 1.4 ppm); ^{1}H NMR (500 MHz, DMSO-d_6, 300 K, one conformer coexists), δ10.22 (s, 1H, PyN*H*), 10.10 (s, 1H, xN*H*), 8.72 (d, 2.5 Hz, 1H, Py$H_{2'}$), 8.47 (m, 1H, Lys$N_{\alpha}H$), 8.24 (d, 3.5 Hz, 1H, Py$H_{6'}$), 8.03–8.00 (m, 2H, x$H_{2'}$, Py$H_{4'}$), 7.80 (d, 8 Hz, 1H, x$H_{4'}$), 7.56 (m, 1H, x$H_{6'}$), 7.37 (m, 1H, x$H_{5'}$), 7.3–7.2 (m, 7H, Py$H_{5'}$, *Ph*CH$_2$, Lys$N_{\varepsilon}H$), 4.98 (s, 2H, OCH_2Ph), 4.66 (m, 1H, LysH_{α}), 4.46 (m, 1H, Pro(2)H_{α}), 4.19 (m, 1H, Pro(1)H_{α}), 3.81, 3.64 (m, 2H, Pro(2)H_{δ}), 3.41, 3.32 (m, 2H, Pro(1)H_{δ}), 2.99 (m, 1H, LysH_{ε}), 2.2 (m, 2H, Pro(1)H_{β}, Pro(2)H_{β}), 2.04 (m, 1H, Pro(2)H_{γ}), 2.0–1.7 (m, 7H, Pro(1)$H_{\beta',\gamma}$, Pro(2)$H_{\beta',\gamma'}$, LysH_{β}), 1.45–1.40 (m 4H, Lys$H_{\gamma,\delta}$), 1.38, 1.25 (m, 9H, *t*-Bu); ^{13}C NMR (125 MHz, DMSO-d_6, 300 K, one conformer coexists), δ172.2 (Pro(1)*C*O), 171.5 (Pro(2)*C*O), 170.9 (Lys*C*O), 166.8 (x*C*O), 156.6 (*C*OCH$_2$Ph), 153.6 (Boc*C*O), 144.6 (Py$C_{6'}$), 141.1 (Py$C_{2'}$), 139.6, 135.2 (x$C_{1'}$, x$C_{3'}$), 137.8 (PhC_i), 136.2 (Py$C_{3'}$), 129.0 (x$C_{5'}$), 128.8, 128.2 (Ph$C_{o,m,p}$), 126.4 (Py$C_{4'}$), 124.1 (Py$C_{5'}$), 122.5 (x$C_{4'}$, x$C_{6'}$), 119.3 (x$C_{2'}$), 79.0 (BocC_q), 65.6 (OCH$_2$Ph), 60.9 (Pro(1)C_{α}), 60.7 (Pro(2)C_{α}), 51.9 (LysC_{α}), 47.4 (Pro(2)C_{δ}), 47.0 (Pro(1)C_{δ}), 40.0 (LysC_{ε}), 31.5 (Pro(1)C_{β}), 30.7 (LysC_{β}), 29.8–29.7 (Pro(2)C_{β}, LysC_{δ}), 28.4 (BocCH_3), 25.2 (Pro(2)C_{γ}), 23.8 (Pro(1)C_{γ}), 21.21 (LysC_{γ}).

Pentapeptide ligand 5K: The hydrolysis of the COOMe group of **S7** was carried out according to procedure *c*. The removal of the Boc group of **S5K** was carried out according to procedure *d*. The hydrolyzed **S7** (0.19 g, 0.9 mmol), Boc-free **S5K** (0.63 g, 0.8 mmol), HOBt (0.16 g, 1.0 mmol), EDCI (0.20 g, 1.0 mmol), and DIEA (0.6 mL, 3.5 mmol) were added in DMF (10 mL) and the mixture was stirred at room temperature. After 3 d, the solvents were removed by evaporation. The crude product was purified by SiO_2 column chromatography (EtOAc/MeOH), and then further purified by using recycle SEC (eluent: MeOH/H_2O). The white powder of **5K** 0.21 g was obtained (Y 27%).

M.p. 129–134 °C; HR-MS: calcd. for $[M+H]^+$: 872.4090, found: 872.4085 (error: 0.6 ppm); ^{1}H NMR (500 MHz, DMSO-d_6, 300 K, two conformers coexist), δ10.26 (s, 1H, Py(C)N*H*), 10.20 (s, 1H, xN*H*), 8.74 (d, 2 Hz, 1H, Py(C)$H_{2'}$), 8.68 (d, 1 Hz, 1H, Py(N)$H_{2'}$), 8.66 (dd, 2 Hz, 5 Hz, 1H, Py(N)$H_{6'}$), 8.50 (m, 1H, Lys$N_{\alpha}H$), 8.26 (dd, 1 Hz, 5 Hz, 1H, Py(C)$H_{6'}$), 8.06–8.02 (m, 2H, Py(C)$H_{4'}$, x$H_{2'}$), 7.90 (dt, 8 Hz, 2 Hz, 1H, Py(N)$H_{4'}$), 7.73 (d, 8 Hz, 1H, x$H_{4'}$), 7.56 (t, 8 Hz, 1H, x$H_{6'}$), 7.48 (dd, 5 Hz, 8 Hz, 1H, Py(N)$H_{5'}$), 7.4–7.3 (m, 7H, x$H_{5'}$, Py(C)$H_{5'}$, Ph), 7.25 (m, 1H, Lys$N_{\varepsilon}H$), 5.00 (s, 1H, OCH_2Ph), 4.80 (m, 1H, Pro(1)H_{α}), 4.68 (m, 1H, LysH_{α}), 4.50–4.47 (m, 2H, Pro(2)H_{α}, Pro(3)H_{α}), 3.82, 3.67–3.53 (m, 4H, Pro(2)H_{δ}, Pro(3)H_{δ}), 3.48 (m, 2H, Pro(1)H_{δ}), 3.01 (m, 1H, LysH_{ε}), 2.33 (m, 1H, Pro(1)H_{β}), 2.2, 2.1–1.7 (m, 13H, Pro(1)$H_{\beta',\gamma}$, Pro(2)$H_{\beta,\gamma}$, Pro(3)$H_{\beta,\gamma}$, LysH_{β}), 1.43 (Lys$H_{\gamma,\delta}$); ^{13}C NMR (125 MHz, DMSO-d_6, 300 K, two conformers coexist), δ171.6 (Pro(3)*C*O), 171.1, 170.8 (Pro(2)*C*O, Lys*C*O), 170.0 (Pro(1)*C*O), 166.9 (x*C*O), 166.1 (Py(N)*C*O), 156.6 (Cbz*C*O), 151.2 (Py(N)$C_{6'}$), 148.2 (Py(N)$C_{2'}$), 144.6 (Py(C)$C_{6'}$), 141.1 (Py(C)$C_{2'}$), 139.6, 135.2 (x$C_{1'}$, x$C_{3'}$), 137.8 (PhC_i), 136.2 (Py(C)$C_{3'}$), 135.1 (Py(N)$C_{4'}$), 132.8 (Py(N)$C_{3'}$), 129.0 (x$C_{5'}$), 128.8, 128.2 (Ph$C_{o,m,p}$), 126.4 (Py(C)$C_{4'}$), 124.1 (Py(C)$C_{5'}$), 123.9 (Py(N)$C_{5'}$), 122.4, 122.2 (x$C_{6'}$, x$C_{4'}$), 119.0 (x$C_{2'}$), 65.6 (OCH$_2$Ph), 60.7, 60.6 (Pro(2)C_{α}, Pro(3)C_{α}), 58.6 (Pro(1)C_{α}), 51.9 (LysC_{α}), 50.1

(Pro(1)C_δ), 47.3, 47.2 (Pro(2)C_δ, Pro(3)C_δ), 39.9 (LysC_ε), 30.8 (Pro(2)C_β), 30.6 (LysC_β), 29.7 (LysC_δ, Pro(3)C_β), 28.6 (Pro(1)C_β), 25.3–25.2, 24.8 (ProC_γ), 23.2 (LysC_γ).

***N*-(3-Pyridyl)-*N'*-Boc-glycyl-L-prolinamide[3] (S4G):** **S4G** was synthesised by condensation of Boc-Gly-OH and Boc-free **S3** by using EDCI/HOBt and DIEA (procedure *e*). The crude product was purified by SiO_2 column chromatography.

***N*-(3-Pyridyl)-*N*^{1,1}-Boc-L-prolylimino-(1,3-phenylene)carbonyl-glycyl-L-prolinamide (S5G):** The hydrolysis of the COOMe group of **S2** was carried out according to procedure *c*. The removal of the Boc group of **S4G** was carried out according to procedure *d*. The hydrolyzed **S2** (0.90 g, 2.7 mmol) was dissolved in $CHCl_3$ (10 mL). To the solution, Boc-free **S4G** (0.87 g, 2.7 mmol), HOBt (0.49 g, 3.2 mmol), EDCI (0.62 g, 3.2 mmol), and DIEA (1.6 mL, 9.4 mmol) were sequentially added. After overnight reaction, the mixture was washed with sat. $NaHCO_3$ aq. (×2). The organic layer was dried over $MgSO_4$ and concentrated by evaporation. The crude product was purified by SiO_2 column chromatography ($CHCl_3$/MeOH). The white solid of **S5G** 0.98 g was obtained (Y 65%).

M.p. 133–144 °C; HR-MS: calcd. for $[M+H]^+$: 565.2769, found: 565.2769 (error 0.0 ppm); ^{1}H NMR (500 MHz, DMSO-d_6, 300 K, one conformer coexists), δ10.19 (s, 1H, PyN*H*), 10.13 (s, 1H, xN*H*), 8.77 (brs, 1H, Py$H_{2'}$), 8.57 (m, 1H, GlyN*H*), 8.26 (d, 4 Hz, 1H, Py$H_{6'}$), 8.08–8.02 (m, 2H, x$H_{2'}$, Py$H_{4'}$), 7.84 (d, 8 Hz, 1H, x$H_{4'}$), 7.56 (m, 1H, x$H_{6'}$), 7.41 (m, 1H, x$H_{5'}$), 7.33 (m, 1H, Py$H_{5'}$), 4.48 (m, 1H, Pro(2)H_α), 4.23, 4.04 (m, 2H, GlyH_α), 4.21 (m, 1H, Pro(1)H_α), 3.69, 3.64 (m, 2H, Pro(2)H_δ), 3.43, 3.35 (m, 2H, Pro(1)H_δ), 2.19 (m, 2H, Pro(1)H_β, Pro(2)H_β), 2.0–1.7 (m, 6H, Pro(1)$H_{\beta',\gamma}$, Pro(2)$H_{\beta',\gamma}$), 1.39, 1.26 (m, 9H, *t*-Bu); ^{13}C NMR (125 MHz, DMSO-d_6, 300 K, one conformer coexists), δ172.1 (Pro(1)*C*O), 171.6 (Pro(2)*C*O), 167.7 (Gly*C*O), 166.8 (x*C*O), 153.6 (Boc*C*O), 144.7 (Py$C_{6'}$), 141.3 (Py$C_{2'}$), 139.7, 135.2 (x$C_{1'}$, x$C_{3'}$), 136.1 (Py$C_{3'}$), 129.0 (x$C_{5'}$), 126.6 (Py$C_{4'}$), 124.1 (Py$C_{5'}$), 122.4, 122.1 (x$C_{4'}$, x$C_{6'}$), 119.1 (x$C_{2'}$), 79.0 (BocC_q), 60.9 (Pro(1)C_α), 60.8 (Pro(2)C_α), 47.0 (Pro(2)C_δ), 46.6 (Pro(1)C_δ), 42.2 (GlyC_α), 31.4 (Pro(1)C_β), 29.8 (Pro(2)C_β), 28.4 (BocCH_3), 25.0 (Pro(2)C_γ), 23.8 (Pro(1)C_γ).

The pentapeptide ligand 5G: The hydrolysis of the COOMe group of **S7** was carried out according to procedure *c*. The removal of the Boc group of **S5G** was carried out according to procedure *d*. The hydrolyzed **S7** (0.38 g, 1.7 mmol), Boc-free **S5G** (0.86 g, 1.6 mmol), HOBt (0.32 g, 2.1 mmol), EDCI (0.40 g, 2.1 mmol), and DIEA (1.2 mL, 6.9 mmol) were added in DMF (20 mL) and the mixture was stirred at room temperature. After 3 d, the solvents were removed by evaporation. The crude product was purified by SiO_2 column chromatography (EtOAc/MeOH), and then further purified by using recycle SEC (eluent: MeOH/H_2O). The white powder of **5G** 0.43 g was obtained (Y 38%).

M.p. 155–168 °C; HR-MS: calcd. for $[M+H]^+$: 667.2987, found: 667.2968 (error: 2.8 ppm); ^{1}H NMR (500 MHz, DMSO-d_6, 300 K, two conformers coexist), δ10.18 (s, 1H, Py(C)N*H*), 10.17 (s, 1H, xN*H*), 8.76 (d, 2 Hz, 1H, Py(C)$H_{2'}$), 8.67 (d, 1 Hz, 1H, Py(N)$H_{2'}$), 8.64 (dd, 2 Hz, 5 Hz, 1H, Py(N)$H_{6'}$), 8.57 (m, 1H, GlyN*H*), 8.24 (d, 5 Hz, 1H, Py(C)$H_{6'}$), 8.09, 8.04–8.00 (m, 2H, Py(C)$H_{4'}$, x$H_{2'}$), 7.88 (dd, 2 Hz, 8 Hz, 1H, Py(N)$H_{4'}$), 7.73 (d, 8 Hz, 1H, x$H_{4'}$), 7.54 (m, 1H, x$H_{6'}$), 7,45 (dd, 5 Hz, 8 Hz, 1H, Py(N)$H_{5'}$), 7.4–7.3 (m, 2H, x$H_{5'}$, Py(C)$H_{5'}$), 4.77 (m, 1H, Pro(1)H_α), 4.5 (m, 2H, Pro(2)H_α, Pro(3)H_α), 4.25–4.21, 4.05–4.00 (m, 2H, GlyH_α), 3.77, 3.66–3.50 (m, 4H, Pro(2)H_δ, Pro(3)H_δ), 3.46 (m, 2H, Pro(1)H_δ), 2.29 (m, 1H, Pro(1)H_β), 2.2, 2.1–1.7 (m, 11H, Pro(1)$H_{\beta',\gamma}$, Pro(2)$H_{\beta,\gamma}$, Pro(3)$H_{\beta,\gamma}$); ^{13}C NMR (125 MHz, DMSO-d_6, 300 K, two conformers coexist), δ171.6 (Pro(3)*C*O), 171.1 (Pro(2)*C*O), 170.0 (Pro(1)*C*O), 167.7 (Gly*C*O), 166.9 (x*C*O), 166.2 (Py(N)*C*O), 151.2 (Py(N)$C_{6'}$), 148.2 (Py(N)$C_{2'}$), 144.7 (Py(C)$C_{6'}$), 141.3 (Py(C)$C_{2'}$), 139.7, 135.2 (x$C_{1'}$, x$C_{3'}$), 136.1 (Py(C)$C_{3'}$), 135.1 (Py(N)$C_{4'}$), 132.8 (Py(N)$C_{3'}$), 129.1 (x$C_{5'}$), 126.6 (Py(C)$C_{4'}$), 124.1 (Py(C)$C_{5'}$), 123.9 (Py(N)$C_{5'}$), 122.2, 122.1 (x$C_{6'}$, x$C_{4'}$), 118.8 (x$C_{2'}$), 60.8, 60.6 (Pro(2)C_α, Pro(3)C_α), 58.6 (Pro(1)C_α), 50.1 (Pro(1)C_δ), 47.3, 47.2 (Pro(2)C_δ, Pro(3)C_δ), 42.2 (GlyC_α), 29.7 (Pro(2)C_β, Pro(3)C_β), 28.6 (Pro(1)C_β), 25.3–25.0 (ProC_γ).

***N*-(3-Pyridyl)-*N'*-Boc-L-prolyl-L-prolinamide**[1] **(S4P): S4P** was synthesised by condensation of Boc-Pro-OH and Boc-free **S3** by using DIEA and ECF (procedure *b*). The crude product was purified by SiO_2 column chromatography.

***N*-(3-Pyridyl)-*N*^{1,1}-Boc-L-prolylimino-(1,3-phenylene)carbonyl-L-prolyl-L-prolinamide (S5P):** The hydrolysis of the COOMe group of **S2** was carried out according to procedure *c*. The removal of the Boc group of **S4P** was carried out according to procedure *d*. The hydrolyzed **S2** (0.67 g, 2.0 mmol) was dissolved in $CHCl_3$ (10 mL). To the solution, Boc-free **S4P** (0.72 g, 2.0 mmol), HOBt (0.37 g, 2.4 mmol), EDCI (0.46 g, 2.4 mmol), and DIEA (1.2 mL, 7.0 mmol) were sequentially added. After overnight reaction, the mixture was washed with sat. $NaHCO_3$ aq. (×2). The organic layer was dried over $MgSO_4$ and concentrated by evaporation. The crude product was purified by SiO_2 column chromatography ($CHCl_3$/MeOH). The white solid of **S5P** 0.99 g was obtained (Y 81%).

M.p. 158–161 ºC; HR-MS: calcd. for $[M+H]^+$: 605.3082, found: 605.3095 (error 2.1 ppm); ^{1}H NMR (500 MHz, DMSO-d_6, 300 K, two conformers coexist), δ10.28 (s, 1H, PyN*H*), 10.11 (s, 1H, xN*H*), 8.74 (brs, 1H, Py$H_{2'}$), 8.25 (brs, 1H, Py$H_{6'}$), 8.05 (d, 8 Hz, 1H, Py$H_{4'}$), 7.82 (s, 1H, x$H_{2'}$), 7.66 (d, 8 Hz, 1H, x$H_{4'}$), 7.38 (m, 1H, x$H_{5'}$), 7.33 (dd, 5 Hz, 8 Hz, Py$H_{5'}$), 7.15 (d, 7 Hz, 1H, x$H_{6'}$), 4.78 (m, 1H,Pro(2)H_α), 4.49 (m, 1H, Pro(3)H_α), 4.18 (m, 1H, Pro(1)H_α), 3.81, 3.65 (m, 2H, Pro(3)H_δ), 3.44 (brs, 2H, Pro(2)H_δ), 3.42, 3.33 (m, 2H, Pro(1)H_δ), 2.31, 2.20, 2.1–1.8 (m, 12H, ProH_β, ProH_γ), 1.39, 1.26 (m, 9H, *t*-Bu); ^{13}C NMR (125 MHz, DMSO-d_6, 300 K, two conformers coexist), δ171.2 (Pro(1)*C*O), 171.6 (Pro(3)*C*O), 170.3 (Pro(2)*C*O), 168.1 (x*C*O), 153.6 (Boc*C*O), 144.6 (Py$C_{6'}$), 141.1 (Py$C_{2'}$), 139.4, 137.6 (x$C_{1'}$, x$C_{3'}$), 136.3 (Py$C_{3'}$), 129.2 (x$C_{5'}$), 126.4 (Py$C_{4'}$), 124.1 (Py$C_{5'}$), 122.1 (x$C_{6'}$), 120.9 (x$C_{4'}$, x$C_{6'}$), 118.3 (x$C_{2'}$), 79.0 (BocC_q), 60.9 (Pro(1)C_α), 60.7 (Pro(3)C_α), 58.4 (Pro(2)C_α), 50.2 (Pro(2)C_δ), 47.4 (Pro(3)C_δ), 47.0 (Pro(1)C_δ), 31.5, 30.6, 29.7 (ProC_β), 28.4 (BocCH_3), 25.2, 23.8 (ProC_γ).

The pentapeptide ligand 5P: The hydrolysis of the COOMe group of **S7** was carried out according to procedure *c*. The removal of the Boc group of **S5P** was carried out according to procedure *d*. The hydrolyzed **S7** (0.30 g, 1.4 mmol), Boc-free **S5P** (0.78 g, 1.4 mmol), HOBt (0.25 g, 1.6 mmol), EDCI (0.31 g, 1.6 mmol), and DIEA (1.0 mL, 5.4 mmol) were added in DMF (10 mL) and the mixture was stirred at room temperature. After 3 d, the solvents were removed by evaporation. The crude product was purified by SiO_2 column chromatography (EtOAc/MeOH), and then further purified by using recycle SEC (eluent: $CHCl_3$). The white powder of **5P** 0.20 g was obtained (Y 21%).

M.p. 178 ºC dec; HR-MS: calcd. for $[M+H]^+$: 707.3300, found: 707.3300 (error: 0.0 ppm); ^{1}H NMR (500 MHz, DMSO-d_6, 300 K, two conformers coexist), δ10.30 (s, 1H, Py(C)N*H*), 10.19 (s, 1H, xN*H*), 8.75 (brs, 1H, Py(C)$H_{2'}$), 8.69 (brs, 1H, Py(N)$H_{2'}$), 8.66 (brs, 1H, Py(N)$H_{6'}$), 8.26 (brs, 1H, Py(C)$H_{6'}$), 8.05 (d, 8Hz, 1H, Py(C)$H_{4'}$), 7.90 (d, 8 Hz, 1H, Py(N)$H_{4'}$), 7.78 (m, 1H, x$H_{2'}$), 7.56 (t, 8 Hz, 1H, x$H_{4'}$), 7.48 (m, 1H, Py(N)$H_{5'}$), 7.38–7.30 (m, 2H, x$H_{5'}$, Py(C)$H_{5'}$), 7.14 (m, 1H, x$H_{6'}$), 4.79 (m, 2H, Pro(1)H_α, Pro(3)H_α), 4.50–4.46 (m, 2H, Pro(2)H_α, Pro(4)H_α), 3.80, 3.64–3.57 (m, 4H, Pro(2)H_δ, Pro(4)H_δ), 3.49, 3.43 (m, 4H, Pro(1)H_δ, Pro(3)H_δ), 2.31, 2.20, 2.1–1.7 (m, 16H, Pro$H_{\beta,\gamma}$); ^{13}C NMR (125 MHz, DMSO-d_6, 300 K, two conformers coexist), δ171.6 (Pro(4)*C*O), 171.2, 170.4 (Pro(2)*C*O, Pro(3)*C*O), 170.0 (Pro(1)*C*O), 168.1 (x*C*O), 166.1 (Py(N)*C*O), 151.2 (Py(N)$C_{6'}$), 148.2 (Py(N)$C_{2'}$), 144.5 (Py(C)$C_{6'}$), 141.0 (Py(C)$C_{2'}$), 139.6, 136.3 (x$C_{1'}$, x$C_{3'}$), 137.7 (Py(C)$C_{3'}$), 135.1 (Py(N)$C_{4'}$), 132.8 (Py(N)$C_{3'}$), 129.1 (x$C_{5'}$), 126.4 (Py(C)$C_{4'}$), 124.1 (Py(C)$C_{5'}$), 123.9 (Py(N)$C_{5'}$), 121.8 (x$C_{4'}$), 120.5 (x$C_{6'}$), 118.0 (x$C_{2'}$), 60.7, 60.6 (Pro(2)C_α, Pro(4)C_α), 58.6 (Pro(1)C_α), 58.3 (Pro(3)C_α), 50.2, 50.1 (Pro(1)C_δ, Pro(3)C_δ), 47.2 (Pro(2)C_δ, Pro(4)C_δ), 30.8 (Pro(2)C_β), 29.7 (Pro(3)C_β, Pro(4)C_β), 28.6 (Pro(1)C_β), 25.3–25.2, 24.8, 23.1 (ProC_γ).

Ligands **5D** and **9** were synthesised according to the following procedure:

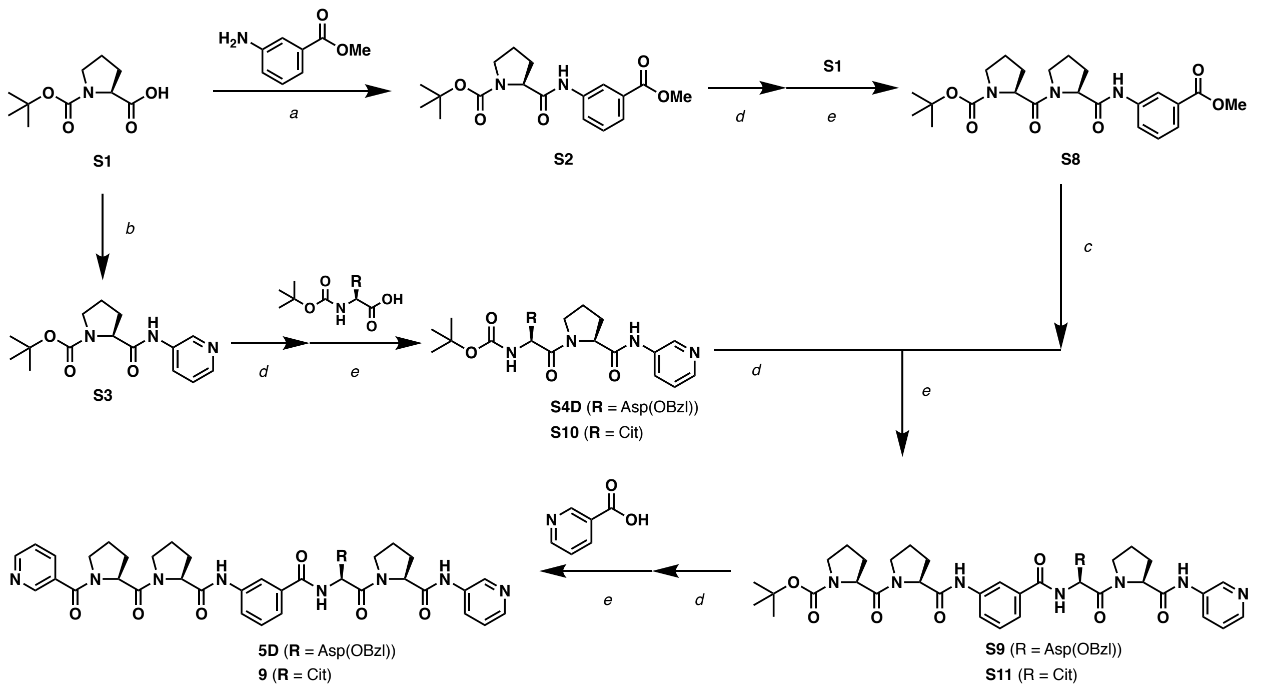

Reagents and conditions: (a) NMM, IBCF, in THF, 0 °C, 20 min, then RT, o/n, (b) DIEA, ECF, in THF, 0 °C, 20 min, then RT, o/n, (c) LiOH, in MeOH/H_2O, 2 h, (d) HCl, in MeOH/1,4-dioxane, 30 min, (e) HOBt/EDCI, DIEA, in $CHCl_3$, o/n.

***N*-(3-Pyridyl)-*N'*-Boc-β-benzyl-L-aspartylprolinamide (S4D):** The removal of the Boc group of **S3** was carried out according to procedure *d*. To the $CHCl_3$ (~10 mL) solution of Boc-free **S3** (0.94 g, 3.6 mmol), Boc-Asp(OBzl)-OH (1.2 g, 3.6 mmol), HOBt (0.66 g, 4.3 mmol), EDCI (0.83 g, 4.3 mmol), and DIEA (2.2 mL, 13 mmol) were sequentially added. The mixture was stirred at room temperature for 2 d. After washed with sat. $NaHCO_3$ aq. (×2), the organic layer was dried over $MgSO_4$ and concentrated by evaporation. The crude product was purified by SiO_2 column chromatography (EtOAc/MeOH). The white solid of **S4D** 1.43 g was obtained (Y 80%).

M.p. 56–61 °C; HR-MS: calcd. for $[M+H]^+$: 497.2395, found: 497.2410 (error 3.0 ppm); ^1H NMR (500 MHz, DMSO-d_6, 300 K, one conformer coexists), δ10.12 (s, 1H, PyN*H*), 8.73 (d, 2 Hz, 1H, Py$H_{2'}$), 8.26 (d, 4 Hz, 1H, Py$H_{6'}$), 8.02 (dt, 8 Hz, 2 Hz, 1H, Py$H_{4'}$), 7.39–7.30 (m, 7H, Py$H_{5'}$, AspN*H*, Ph), 5.09 (q, 12 Hz, 2H, PhCH_2), 4.68 (dd, 13 Hz, 8 Hz, 1H, AspH_α), 4.45 (m, 1H, ProH_α), 3.66 (m, 2H, ProH_δ), 2.80, 2.61 (m, 2H, AspH_β), 2.16, 2.02–1.91 (m, 4H, Pro$H_{\beta,\gamma}$), 1.37 (s, 9H, *t*-Bu); ^{13}C NMR (125 MHz, DMSO-d_6, 300 K, one conformer coexists), δ171.4 (Pro*C*O), 170.6 (Asp*C*O_β), 169.7 (Asp*C*O_α), 155.6 (Boc*C*O), 144.7 (Py$C_{6'}$), 141.3 (Py$C_{2'}$), 136.5 (PhC_i), 136.1 (Py$C_{3'}$), 128.8, 128.4 (Ph$C_{o,m,p}$), 126.6 (Py$C_{4'}$), 124.1 (Py$C_{5'}$), 78.8 (BocC_q), 66.2 (Ph*C*H_2), 60.9 (ProC_α), 49.5 (AspC_α), 47.2 (ProC_δ), 36.3 (AspC_β), 29.7 (ProC_β), 28.6 (Boc*C*H_3), 25.2 (ProC_γ).

Methyl 3-(*N*-Boc-L-prolyl-L-prolylimino)-benzoate (S8): The removal of the Boc group of **S2** was carried out according to procedure *d*. Boc-free **S2** (2.7 g, 8.5 mmol) was dissolved in $CHCl_3$. To the solution, **S1** (1.8 g, 8.5 mmol), HOBt (1.6 g, 10 mmol), EDCI (2.0 g, 10 mmol), and DIEA (3.7 mL, 21 mmol) were sequentially added and stirred at room temperature for overnight. The mixture was washed with sat. $NaHCO_3$ aq. (×2), 5% citric acid, and brine. The organic layer was dried over $MgSO_4$ and concentrated by evaporation. The crude product was purified by using recycle SEC (eluent: $CHCl_3$). The white solid of **S8** 1.8 g was obtained (Y 47%).

M.p. 86–91 ºC; HR-MS: calcd. for $[M+Na]^+$: 468.2105, found: 468.2094 (error 2.3 ppm); ^1H NMR (500 MHz, DMSO-d_6, 300 K, one conformer coexists), δ10.26 (s, 1H, PyN*H*), 8.31 (s, 1H, x$H_{2'}$), 7.79 (m, 7 Hz, 1H, x$H_{4'}$), 7.64 (d, 7.5 Hz, 1H, x$H_{6'}$), 7.46 (t, 8 Hz, 1H, x$H_{5'}$), 4.43 (m, 2H, Pro(1)H_α, Pro(2)H_α), 3.76 (s, 3H, OCH_3), 3.70–3.53 (m, 2H, Pro(2)H_δ), 3.30 (t, 6.5 Hz, 2H, Pro(1)H_δ), 2.2–1.7 (m, 8H, Pro$H_{\beta,\gamma}$), 1.38 (s, 9H, *t*-Bu); ^{13}C NMR (125 MHz, DMSO-d_6, 300 K, one conformer coexists), δ171.4 (Pro(1)*C*O), 171.2 (Pro(2)*C*O), 166.6 (x*C*O), 153.9 (Boc*C*O), 140.0, 130.5 (x$C_{1'}$, x$C_{3'}$), 129.7 (x$C_{5'}$), 124.2 (x$C_{6'}$), 123.9 (x$C_{4'}$), 119.9 (x$C_{2'}$), 78.9 (BocC_q), 60.6 (Pro(2)C_α), 57.8 (Pro(1)C_α), 52.7 (O*C*H_3), 47.1, 46.9 (Pro(1)C_δ, Pro(2)C_δ), 30.0 (Pro(1)C_β), 29.7 (Pro(2)C_β), 28.4 (Boc*C*H_3), 25.1 (Pro(2)C_γ), 23.7 (Pro(1)C_γ).

***N*-(3-Pyridyl)-$N^{1,1}$-Boc-L-prolyl-L-prolylimino-(1,3-phenylene)carbonyl-β-benzyl-L-aspartyl-L-prolinamide (S9):** The hydrolysis of the COOMe group of **S8** was carried out according to procedure *c*. The removal of the Boc group of **S4D** was carried out according to procedure *d*. The hydrolyzed **S8** (1.73 g, 4.0 mmol) was dissolved in $CHCl_3$ (15 mL). To the solution, Boc-free **S4D** (1.89 g, 4.0 mmol), HOBt (0.74 g, 4.8 mmol), EDCI (0.92 g, 4.8 mmol), and DIEA (2.5 mL, 14 mmol) were sequentially added and stirred at room temperature for overnight. After washed with sat. $NaHCO_3$ aq. (×2), the organic layer was dried over $MgSO_4$ and concentrated by evaporation. The crude product was purified by SiO_2 column chromatography ($CHCl_3$/MeOH). The white solid of **S9** 3.0 g was obtained (Y 94%).

M.p. 132–137 ºC; HR-MS: calcd. for $[M+H]^+$: 810.3821, found: 810.3825 (error 0.5 ppm); ^1H NMR (500 MHz, DMSO-d_6, 300 K, two conformers coexist), δ10.17 (s, 1H, xN*H*), 10.12 (s, 1H, PyN*H*), 8.87 (d, 8 Hz, 1H, AspN$_\alpha$*H*), 8.74 (m, 1H, Py$H_{2'}$), 8.26 (dd, 1 Hz, 4.5 Hz, 1H, Py$H_{6'}$), 8.08 (s, 1H, x$H_{2'}$), 8.02 (d, 9 Hz, 1H, Py$H_{4'}$), 7.72 (d, 8 Hz, 1H, x$H_{4'}$), 7.53 (d, 7.5 Hz, 1H, x$H_{6'}$), 7.39 (m, 1H, x$H_{5'}$), 7.35–7.28 (m, 6H, Py$H_{5'}$, Ph), 5.18 (m, 1H, AspH_α), 5.10 (m, 2H, PhCH_2), 4.50–4.41 (m, 3H, Pro(1)H_α, Pro(2)H_α, Pro(3)H_α,), 3.73 (m, 2H, Pro(3)H_δ), 3.61 (m, 2H, Pro(2)H_δ), 3.30 (m, 2H, Pro(1)H_δ), 2.93–2.83 (m, 2H, AspH_β), 2.25–1.7 (m, 12H, Pro$H_{\beta,\gamma}$, Gln$H_{\beta,\gamma}$), 1.37, 1.32 (m, 9H, *t*-Bu); ^{13}C NMR (125 MHz, DMSO-d_6, 300 K, two conformers coexist), δ171.4–170.7 (Pro(1)*C*O, Pro(2)*C*O, Asp*C*O, Pro(3)*C*O), 169.4 (AspC_βO), 166.6 (x*C*O), 153.5 (Boc*C*O), 144.7 (Py$C_{6'}$), 141.3 (Py$C_{2'}$), 139.7 (X$C_{3'}$), 136.4 (PhC_i), 136.1 (Py$C_{3'}$), 134.8 (x$C_{1'}$), 129.1 (x$C_{5'}$), 128.8, 128.4, 128.2 (Ph$C_{o,m,p}$), 126.6 (Py$C_{4'}$), 124.1 (Py$C_{5'}$), 122.4 (x$C_{4'}$, x$C_{6'}$), 119.0 (x$C_{2'}$), 78.7 (BocC_q), 66.3 (Ph*C*H_2), 60.9 (Pro(3)C_α), 60.5 (Pro(2)C_α), 57.8 (Pro(1)C_α), 52.1 (Pro(2)C_δ), 48.8 (AspC_α), 47.4–46.9 (Pro(1)C_δ, Pro(3)C_δ), 36.1 (AspC_β), 30.0, 29.7, 29.6 (Pro(1)C_β, Pro(2)C_β, Pro(3)C_β), 28.5 (Boc*C*H_3), 25.2, 25.1, 24.2 (Pro(1)C_γ, Pro(2)C_γ, Pro(3)C_γ).

Pentapeptide ligand 5D: The removal of the Boc group of **S9** was carried out according to procedure *d*. Nicotinic acid (0.42 g, 3.4 mmol), Boc-free **S9** (2.7 g, 3.4 mmol), HOBt (0.63 g, 4.1 mmol), EDCI (0.78 g, 4.1 mmol), and DIEA (2.1 mL, 12 mmol) were added in $CHCl_3$ (15 mL) and the mixture was stirred at room temperature for 2 d. The solvents were removed by evaporation. The crude product was purified by SiO_2 column chromatography (EtOAc/MeOH), and then further purified by using recycle SEC (eluent: $CHCl_3$). The white powder of **5D** 0.80 g was obtained (Y 29%).

M.p. 148–152 ºC dec; HR-MS: calcd. for $[M+H]^+$: 815.3511, found: 815.3535 (error: 2.9 ppm); ^1H NMR (500 MHz, DMSO-d_6, 300 K, two conformers coexist), δ10.21 (s, 1H, xN*H*), 10.13 (s, 1H, Py(C)N*H*), 8.87 (d, 7.5 Hz, 1H, AspN$_\alpha$*H*), 8.75 (m, 1H, Py(C)$H_{2'}$), 8.69 (d, 1.5 Hz, 1H, Py(N)$H_{2'}$), 8.66 (dd, 1.5 Hz, 5 Hz, 1H, Py(N)$H_{6'}$), 8.26 (dd, 1 Hz, 4.5 Hz, 1H, Py(C)$H_{6'}$), 8.10, 8.03 (m, 2H, x$H_{2'}$, Py(C)$H_{4'}$), 7.91 (d, 8 Hz, 1H, Py(N)$H_{4'}$), 7.73 (d, 8 Hz, 1H, x$H_{4'}$), 7.53 (m, 1H, x$H_{6'}$), 7.49 (dd, 5 Hz, 8 Hz, 1H, Py(N)$H_{5'}$), 7.40 (t, 8 Hz, 1H, x$H_{5'}$), 7.37–7.28 (m, 6H, Py(C)$H_{5'}$, Ph), 5.18–5.10 (m, 3H, AspH_α, PhCH_2), 4.81 (dd, 4.5 Hz, 8 Hz, 1H, Pro(1)H_α), 4.51–4.46 (m, 2H, Pro(2)H_α, Pro(3)H_α), 3.8–3.6 (m, 4H, Pro(2)H_δ, Pro(3)H_δ), 3.50 (m, 2H, Pro(1)H_δ), 2.98–2.73 (m, 2H, AspH_β), 2.33 (m, 1H, Pro(1)H_β), 2.2, 2.1–1.7 (m, 11H, Pro(1)$H_{\beta',\gamma}$, Pro(2)$H_{\beta,\gamma}$, Pro(3)$H_{\beta,\gamma}$); ^{13}C NMR (125 MHz, DMSO-d_6, 300 K, two conformers coexist), δ171.3–170.7 (Pro(1)*C*O, Pro(2)*C*O, Asp*C*O, Pro(3)*C*O), 169.4 (AspC_βO), 166.6 (x*C*O), 166.1 (Py(N)*C*O), 151.2 (Py(N)$C_{6'}$), 148.2 (Py(N)$C_{2'}$), 144.7 (Py(C)$C_{6'}$), 141.3 (Py(C)$C_{2'}$), 139.7, 134.8 (x$C_{1'}$, x$C_{3'}$), 136.4 (PhC_i), 136.2 (Py(C)$C_{3'}$), 135.2 (Py(N)$C_{4'}$), 132.8 (Py(N)$C_{3'}$), 129.1 (x$C_{5'}$), 128.8, 128.4, 128.3 (Ph$C_{o,m,p}$), 126.6 (Py(C)$C_{4'}$), 124.1 (Py(C)$C_{5'}$), 123.9 (Py(N)$C_{5'}$), 122.4 (x$C_{6'}$, x$C_{4'}$), 119.0 (x$C_{2'}$), 66.3 (Ph*C*H_2), 61.0, (Pro(3)C_α), 60.6 (Pro(2)C_α), 58.6 (Pro(1)C_α), 50.1 (Pro(1)C_δ), 48.8 (AspC_α), 47.4–46.9 (Pro(2)C_δ, Pro(3)C_δ), 36.1 (AspC_β), 29.7, 28.6 (Pro(1)C_β, Pro(2)C_β, Pro(3)C_β), 25.4–24.9 (Pro(1)C_γ, Pro(2)C_γ, Pro(3)C_γ).

***N*-(3-Pyridyl)-*N'*-Boc-L-citrullylprolinamide (S10):** The removal of the Boc group of **S3** was carried out according to procedure *d*. To the $CHCl_3$ (~10 mL) solution of Boc-free **S3** (0.45 g, 1.7 mmol), Boc-Cit-OH (0.47 g, 1.7 mmol), HOBt (0.31 g, 2.0 mmol), EDCI (0.38 g, 2.0 mmol), and DIEA (1.0 mL, 6.0 mmol) were sequentially added. The mixture was stirred at room temperature for overnight. After washed with sat. $NaHCO_3$ aq. (×2), the organic layer was dried over $MgSO_4$ and concentrated by evaporation. The crude product was purified by SiO_2 column chromatography ($CHCl_3$/MeOH). The white solid of **S10** 0.47 g was obtained (Y 59%).

M.p. 102–107 ºC; HR-MS: calcd. for $[M+H]^+$: 449.2507, found: 449.2528 (error 4.7 ppm); ^1H NMR (500 MHz, DMSO-d_6, 300 K, one conformer coexists), δ10.21 (s, 1H, PyN*H*), 8.70 (s, 1H, Py$H_{2'}$), 8.23 (d, 4.5 Hz, 1H, Py$H_{6'}$), 8.01 (d, 8 Hz, 1H, Py$H_{4'}$), 7.32 (dd, 4.5 Hz, 8 Hz, 1H, Py$H_{5'}$), 6.93 (d, 7.5 Hz, 1H, CitN*H*), 5.89 (brs, 1H, CitN$_\delta$*H*), 5.37 (s, 2H, CitNH_2), 4.43 (m, 1H, ProH_α), 4.16 (m, 1H, CitH_α), 3.68, 3.58 (m, 2H, ProH_δ), 2.94 (m, 2H, CitH_δ), 2.16, 2.02–1.85 (m, 4H, Pro$H_{\beta,\gamma}$), 1.60, 1.43 (m, 4H, Cit$H_{\beta,\gamma}$), 1.35 (s, 9H, *t*-Bu); ^{13}C NMR (125 MHz, DMSO-d_6, 300 K, one conformer coexists), δ171.6 (Pro*C*O), 171.2 (Cit*C*O), 159.2 (Cit*C*ONH_2), 155.9 (Boc*C*O), 144.6 (Py$C_{6'}$), 141.1 (Py$C_{2'}$), 136.2 (Py$C_{3'}$), 126.4 (Py$C_{4'}$), 124.1 (Py$C_{5'}$), 78.4 (BocC_q), 60.6 (ProC_α), 52.3 (CitC_α), 47.2 (ProC_δ), 39.3 (CitC_δ), 29.7 (ProC_β), 28.6 (BocCH_3, CitC_β), 26.7 (CitC_γ), 25.2 (ProC_γ).

***N*-(3-Pyridyl)-*N*^{1,1}-Boc-L-prolyl-L-prolylimino-(1,3-phenylene)carbonyl-L-citrullyl-L-prolinamide (S11):** The hydrolysis of the COOMe group of **S8** was carried out according to procedure *c*. The removal of the Boc group of **S10** was carried out according to procedure *d*. The hydrolyzed **S10** (0.70 g, 1.7 mmol) was dissolved in $CHCl_3$ (10 mL). To the solution, Boc-free **S10** (0.81 g, 1.7 mmol), HOBt (0.31 g, 2.0 mmol), EDCI (0.39 g, 2.0 mmol), and DIEA (1.0 mL, 5.9 mmol) were sequentially added and stirred at room temperature for overnight. After washed with sat. $NaHCO_3$ aq. (×2), the organic layer was dried over $MgSO_4$ and concentrated by evaporation. The crude product was recrystallised from EtOAc($CHCl_3$/MeOH). The white solid of **S11** 0.83 g was obtained (Y 63%).

M.p. 141–146 ºC; HR-MS: calcd. for $[M+H]^+$: 762.3933, found: 762.3969 (error 4.7 ppm); ^1H NMR (500 MHz, DMSO-d_6, 300 K, two conformers coexist), δ10.24 (s, 1H, xN*H*), 10.15 (s, 1H, PyN*H*), 8.72 (m, 1H, Py$H_{2'}$), 8.54 (d, 12 Hz, 1H, CitN$_\alpha$*H*), 8.24 (d, 4 Hz, 1H, Py$H_{6'}$), 8.03, 8.01 (m, 2H, x$H_{2'}$, Py$H_{4'}$), 7.69 (d, 7.5 Hz, 1H, x$H_{4'}$), 7.55 (d, 8 Hz, 1H, x$H_{6'}$), 7.36 (t, 7.5 Hz, 1H, x$H_{5'}$), 7.32 (m, 1H, Py$H_{5'}$), 5.93 (brs, 1H, CitN$_\delta$*H*), 5.38 (s, 2H, CitNH_2), 4.68, 4.46–4.40 (m, 3H, Pro(1)H_α, Pro(2)H_α, Pro(3)H_α, CitH_α), 3.81 (m, 2H, Pro(3)H_δ), 3.66, 3.55 (m, 2H, Pro(2)H_δ), 3.30 (m, 2H, Pro(1)H_δ), 2.98 (m, 2H, CitH_δ), 2.2–1.6 (m, 14H, Pro$H_{\beta,\gamma}$, CitH_β), 1.48 (m, 2H, CitH_γ), 1.35, 1.30 (m, 9H, *t*-Bu); ^{13}C NMR (125 MHz, DMSO-d_6, 300 K, two conformers coexist), δ171.5–170.7 (Pro(1)*C*O, Pro(2)*C*O, Cit*C*O, Pro(3)*C*O), 166.6 (x*C*O), 159.3 (Cit*C*ONH_2), 153.5 (Boc*C*O), 144.6 (Py$C_{6'}$), 141.2 (Py$C_{2'}$), 139.6 (X$C_{3'}$), 136.2 (Py$C_{3'}$), 135.2 (x$C_{1'}$), 129.0 (x$C_{5'}$), 126.5 (Py$C_{4'}$), 124.1 (Py$C_{5'}$), 122.4, 122.3 (x$C_{4'}$, x$C_{6'}$), 119.0 (x$C_{2'}$), 78.7 (BocC_q), 60.8 (Pro(3)C_α), 60.5 (Pro(2)C_α), 57.8 (Pro(1)C_α), 51.7 (CitC_α), 47.4–46.9 (Pro(1)C_δ, Pro(2)C_δ, Pro(3)C_δ), 39.3 (CitC_δ), 30.0, 29.8, 29.7 (Pro(1)C_β, Pro(2)C_β, Pro(3)C_β), 28.5 (BocCH_3, CitC_β), 26.9 (CitC_γ), 25.2, 25.1, 24.2 (Pro(1)C_γ, Pro(2)C_γ, Pro(3)C_γ).

Pentapeptide ligand 9: The removal of the Boc group of **S11** was carried out according to procedure *d*. Nicotinic acid (0.13 g, 1.0 mmol), Boc-free **S11** (0.78 g, 1.0 mmol), HOBt (0.19 g, 1.2 mmol), EDCI (0.24 g, 1.2 mmol), and DIEA (0.6 mL, 3.5 mmol) were added in $CHCl_3$ (15 mL) and the mixture was stirred at room temperature. After overnight reaction, the solution was concentrated by evaporation. The crude product was purified by SiO_2 column chromatography (EtOAc/MeOH), and then further purified by using recycle SEC (eluent: MeOH/H_2O). The white powder of **9** 0.41 g was obtained (Y 51%).

M.p. 177–182 ºC dec; HR-MS: calcd. for $[M+H]^+$: 767.3624, found: 767.3651 (error: 3.5 ppm); ^1H NMR (500 MHz, DMSO-d_6, 300 K, two conformers coexist), δ10.24 (s, 1H, xN*H*), 10.17 (s, 1H, Py(C)N*H*), 8.72 (brs, 1H, Py(C)$H_{2'}$), 8.66 (brs, 1H, Py(N)$H_{2'}$), 8.64 (d, 4.5 Hz, 1H, Py(N)$H_{6'}$), 8.53 (m, 1H, CitN$_\alpha$*H*), 8.24 (dd, 1 Hz, 4.5 Hz, 1H, Py(C)$H_{6'}$), 8.04–8.00 (m, 2H, x$H_{2'}$, Py(C)$H_{4'}$), 7.88 (d, 8 Hz, 1H, Py(N)$H_{4'}$), 7.71 (d, 8 Hz, 1H, x$H_{4'}$), 7.55 (m, 1H, x$H_{6'}$), 7.46 (m, 1H, Py(N)$H_{5'}$), 7.36 (t, 8 Hz, 1H, x$H_{5'}$), 7.33 (m, 1H, Py(C)$H_{5'}$), 5.93 (brs, 1H, CitN$_\delta$*H*), 5.38 (brs, 2H, CitNH_2), 4.78 (dd, 5 Hz, 7.5 Hz, 1H, Pro(1)H_α), 4.68 (m,

1H, CitH_{α}), 4.47 (m, 2H, Pro(2)H_{α}, Pro(3)H_{α}), 4.48–4.44 (m, 2H, Pro(2)H_{α}, Pro(3)H_{α}), 3.8–3.6 (m, 4H, Pro(2)H_{δ}, Pro(3)H_{δ}), 3.47 (m, 2H, Pro(1)H_{δ}), 2.98 (m, 2H, CitH_{δ}), 2.33 (m, 1H, Pro(1)H_{β}), 2.2, 2.1–1.7 (m, 11H, Pro(1)$H_{\beta',\gamma}$, Pro(2)$H_{\beta,\gamma}$, Pro(3)$H_{\beta,\gamma}$), 1.7–1.4 (m, 4H, CitN$H_{\beta,\gamma}$); ^{13}C NMR (125 MHz, DMSO-d_6, 300 K, two conformers coexist), δ171.6, 171.1, 170.8, 170.0 (Pro(1)CO, Pro(2)CO, Cit$C$O, Pro(3)CO), 166.9 (xCO), 166.1 (Py(N)CO), 159.2 (Cit$C$ONH$_2$), 151.2 (Py(N)$C_{6'}$), 148.2 (Py(N)$C_{2'}$), 144.5 (Py(C)$C_{6'}$), 141.0 (Py(C)$C_{2'}$), 139.6, 135.1 (x$C_{1'}$, x$C_{3'}$), 136.3 (Py(C)$C_{3'}$), 135.2 (Py(N)$C_{4'}$), 132.8 (Py(N)$C_{3'}$), 129.0 (x$C_{5'}$), 126.6 (Py(C)$C_{4'}$), 124.1 (Py(C)$C_{5'}$), 123.9 (Py(N)$C_{5'}$), 122.4, 122.3 (x$C_{6'}$, x$C_{4'}$), 119.0 (x$C_{2'}$), 60.8 (Pro(3)C_{α}), 60.6 (Pro(2)C_{α}), 58.6 (Pro(1)C_{α}), 51.7 (CitC_{α}), 50.1 (Pro(1)C_{δ}), 47.4–46.8 (Pro(2)C_{δ}, Pro(3)C_{δ}), 39.3 (CitC_{δ}), 29.7, 28.6 (Pro(1)C_{β}, Pro(2)C_{β}, Pro(3)C_{β}), 28.4 (CitC_{β}), 26.9 (CitC_{γ}), 25.4–24.9 (Pro(1)C_{γ}, Pro(2)C_{γ}, Pro(3)C_{γ}).

Guest-tethered ligands **7** and **8** were synthesised according to the following procedure:

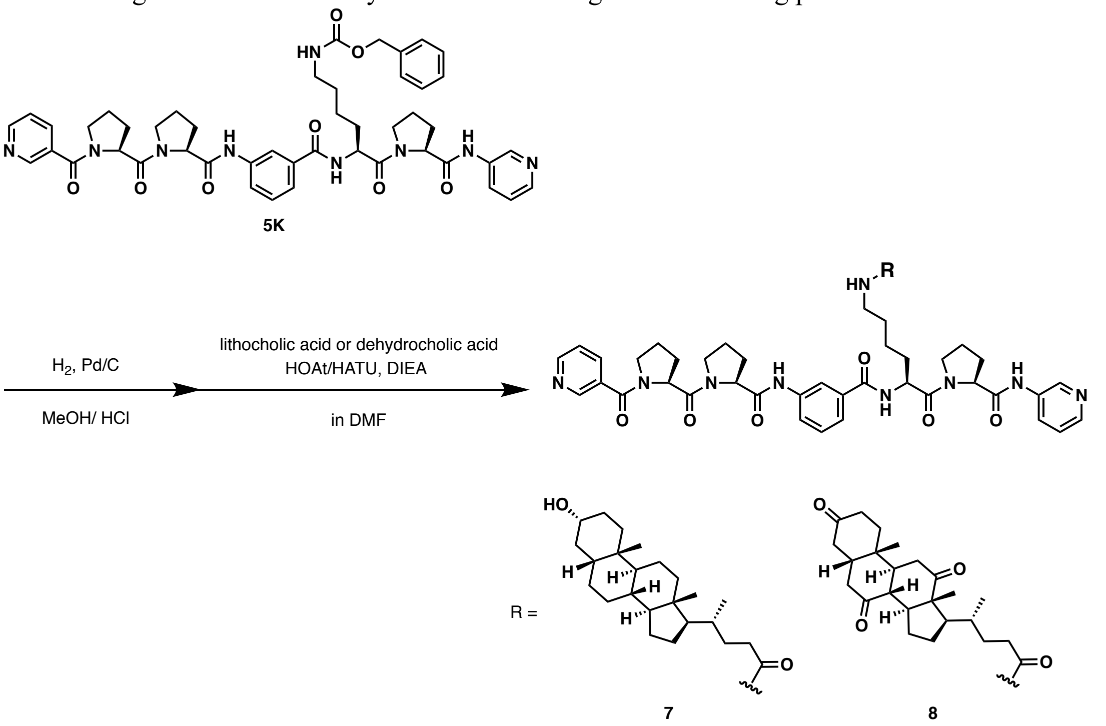

Pentapeptide ligand 7 (guest: lithocholic acid)**:** The deprotection of the Cbz group was carried out as follows: To the MeOH solution of **1K** (50 mg, 57 μmol), 10% Pd/C (106 mg) and 1N HCl aq. (0.25 mL) were sequentially added and the mixture was stirred under the H_2 (5 atm) atmosphere at room temperature for 12 h. The resulting mixture was passed through Celite and the solvent was removed by evaporation. White powder of Cbz-free **1K**•(HCl)$_3$ (49 mg) was obtained. The deprotection of the Cbz group was confirmed by ^{1}H NMR measurement. The Cbz-free **1K** (50 mg, 70 μmol) was dissolved in DMF (~10 mL). To the solution, lithocholic acid (54 mg, 0.14 mmol), HATU (65 mg, 0.17 mmol), HOAt (23 mg, 0.17 mmol), and DIEA (50 μL, 0.30 mmol) were sequentially added and stirred at room temperature. After 3 d, the solvents were removed by evaporation. The crude product was purified by SiO_2 column chromatography (EtOAc/MeOH), and then further purified by using recycle SEC (eluent: $CHCl_3$). The white powder of **7** 38 mg was obtained (Y 50%).

M.p. 172–184 °C dec; HR-MS: calcd. For [M+H]$^+$: 1096.6594, found: 1096.6597 (error: 0.3 ppm); ^{1}H NMR (500 MHz, DMSO-d_6, 300 K, two conformers coexist), δ10.25 (s, 1H, Py(C)NH), 10.18 (s, 1H, xNH), 8.74 (d, 2 Hz, 1H, Py(C)$H_{2'}$), 8.68 (d, 1 Hz, 1H, Py(N)$H_{2'}$), 8.66 (dd, 1.5 Hz, 5 Hz, 1H, Py(N)$H_{6'}$), 8.48 (t, 7 Hz, 1H, LysN$_{\alpha}$$H$), 8.25 (dd, 1 Hz, 4.5 Hz, 1H, Py(C)$H_{6'}$), 8.06–8.00 (m, 2H, Py(C)$H_{4'}$, x$H_{2'}$), 7.91 (td, 2 Hz, 8 Hz, 1H, Py(N)$H_{4'}$), 7.74–7.73 (m, 2H, LysN$_{\varepsilon}$$H$, x$H_{4'}$), 7.57 (t, 8 Hz, 1H, x$H_{6'}$), 7.49 (dd, 5 Hz, 8 Hz, 1H, Py(N)$H_{5'}$), 7.40–7.32 (m, 2H, x$H_{5'}$, Py(C)$H_{5'}$), 4.80 (m, 1H, Pro(1)H_{α}), 4.66 (m, 1H, LysH_{α}), 4.50–4.44 (m, 2H, Pro(2)H_{α}, Pro(3)H_{α}), 3.82, 3.67–3.62 (m, 4H, Pro(2)H_{δ}, Pro(3)H_{δ}), 3.50 (m, 2H, Pro(1)H_{δ}), 3.07–2.96 (m, 5H), 2.33, 2.21, 2.1–1.6 (m, 22H), 1.5–1.0 (m, 23H), 0.86 (s, 3H, Me), 0.84 (s, 3H, Me), 0.58 (s, 3H, Me); ^{13}C NMR (125 MHz, DMSO-d_6, 300 K, two conformers coexist), δ172.9

(C(24)ONH), 171.5 (Pro(3)CO), 171.1, 170.9 (Pro(2)CO, Lys$C$O), 170.0 (Pro(1)CO), 166.8 (xCO), 166.1 (Py(N)CO), 151.2 (Py(N)$C_{6'}$), 148.2 (Py(N)$C_{2'}$), 144.6 (Py(C)$C_{6'}$), 141.1 (Py(C)$C_{2'}$), 139.6, 135.2 (x$C_{1'}$, x$C_{3'}$), 136.2 (Py(C)$C_{3'}$), 135.1 (Py(N)$C_{4'}$), 132.8 (Py(N)$C_{3'}$), 129.0 (x$C_{5'}$), 126.4 (Py(C)$C_{4'}$), 124.1 (Py(C)$C_{5'}$), 123.9 (Py(N)$C_{5'}$), 122.4, 122.2 (x$C_{6'}$, x$C_{4'}$), 119.0 (x$C_{2'}$), 70.3, 60.7, 60.6 (Pro(2)C_{α}, Pro(3)C_{α}), 58.6 (Pro(1)C_{α}), 56.5, 55.9, 51.9 (LysC_{α}), 50.1 (Pro(1)C_{δ}), 47.4, 47.2 (Pro(2)C_{δ}, Pro(3)C_{δ}), 42.7, 42.0, 39.9 (LysC_{ε}), 38.5, 36.8, 35.8, 35.6, 35.3, 34.7, 32.8, 32.0, 30.8 (Pro(2)C_{β}), 30.6 (LysC_{β}), 29.7 (LysC_{δ}, Pro(3)C_{β}), 29.5, 28.6 (Pro(1)C_{β}), 28.1, 27.3, 26.6, 25.3–25.2, 24.8 (ProC_{γ}), 24.3, 23.8, 23.2 (LysC_{γ}), 20.8, 18.7, 12.3. (Unlabeled chemical shifts are derived from the lithocholic acid moiety.)

Pentapeptide ligand 8 (guest: dehydrocholic acid)**:** The deprotection of the Cbz group was carried out by the same procedure as **7**. The Cbz-free **5K** (36 mg, 50 μmol) was dissolved in DMF (~10 mL). To the solution, dehydrocholic acid (40 mg, 100 μmol), HATU (45 mg, 120 μmol), HOAt (16 mg, 120 μmol), and DIEA (50 μL, 0.30 mmol) were sequentially added and stirred at room temperature. After 3 d, the solvents were removed by evaporation. The crude product was purified by SiO_2 column chromatography (EtOAc/MeOH), and then further purified by using recycle SEC (eluent: $CHCl_3$). The white powder of **8** 16 mg was obtained (Y 29%).

M.p. 180–185 ºC dec; HR-MS: calcd. For $[M+H]^+$: 1122.6023, found: 1122.6002 (error: 1.9 ppm); ^{1}H NMR (500 MHz, DMSO-d_6, 300 K, two conformers coexist), δ10.24 (s, 1H, Py(C)NH), 10.17 (s, 1H, XNH), 8.72 (d, 1.5 Hz, 1H, Py(C)$H_{2'}$), 8.68 (brs, 1H, Py(N)$H_{2'}$), 8.66 (d, 6.5 Hz, 1H, Py(N)$H_{6'}$), 8.48 (t, 8 Hz, 1H, LysN$_{\alpha}H$), 8.25 (d, 4.5 Hz, 1H, Py(C)$H_{6'}$), 8.06–8.00 (m, 2H, Py(C)$H_{4'}$, X$H_{2'}$), 7.90 (d, 8 Hz, 1H, Py(N)$H_{4'}$), 7.75 (d, 5.5 Hz, 1H, LysN$_{\varepsilon}H$), 7.72 (d, 9 Hz, 1H, X$H_{4'}$), 7.56 (t, 7.5 Hz, 1H, X$H_{6'}$), 7.48 (dd, 5 Hz, 8 Hz, 1H, Py(N)$H_{5'}$), 7.40–7.32 (m, 2H, X$H_{5'}$, Py(C)$H_{5'}$), 4.80 (m, 1H, Pro(1)H_{α}), 4.66 (m, 1H, LysH_{α}), 4.50–4.46 (m, 2H, Pro(2)H_{α}, Pro(3)H_{α}), 3.82, 3.67–3.62 (m, 4H, Pro(2)H_{δ}, Pro(3)H_{δ}), 3.49 (m, 2H, Pro(1)H_{δ}), 3.05–2.96 (m, 5H), 2.81 (t, 12 Hz, 1H), 2.45 (m, 1H), 2.4–1.6 (m, 27H), 1.48–1.41 (m, 5H), 1.30 (s, 3H, Me), 1.25–1.15 (m, 5H), 0.98 (s, 3H, Me), 0.75 (d, 5.5 Hz, 3H, Me); ^{13}C NMR (125 MHz, DMSO-d_6, 300 K, two conformers coexist), δ212.4 (C(12)O), 210.0 (C(3)O, C(7)O), 172.7 (C(24)ONH), 171.6 (Pro(3)CO), 171.1, 170.9 (Pro(2)CO, Lys$C$O), 170.0 (Pro(1)CO), 166.9 (XCO), 166.1 (Py(N)CO), 151.2 (Py(N)$C_{6'}$), 148.2 (Py(N)$C_{2'}$), 144.6 (Py(C)$C_{6'}$), 141.1 (Py(C)$C_{2'}$), 139.6, 135.2 (X$C_{1'}$, X$C_{3'}$), 136.2 (Py(C)$C_{3'}$), 135.1 (Py(N)$C_{4'}$), 132.8 (Py(N)$C_{3'}$), 129.0 (X$C_{5'}$), 126.4 (Py(C)$C_{4'}$), 124.1 (Py(C)$C_{5'}$), 123.9 (Py(N)$C_{5'}$), 122.4, 122.2 (X$C_{6'}$, X$C_{4'}$), 119.0 (X$C_{2'}$), 60.7, 60.6 (Pro(2)C_{α}, Pro(3)C_{α}), 58.6 (Pro(1)C_{α}), 56.7, 51.9 (LysC_{α}), 51.6, 50.1 (Pro(1)C_{δ}), 49.1, 48.4, 47.4, 47.2 (Pro(2)C_{δ}, Pro(3)C_{δ}), 46.5, 45.8, 45.0, 44.4, 43.0, 39.9 (LysC_{ε}), 38.8, 38.6, 36.6, 36.1, 35.5, 35.0, 33.2, 31.7, 30.8 (Pro(2)C_{β}), 30.6 (LysC_{β}), 29.8, 29.7 (LysC_{δ}, Pro(3)C_{β}), 29.4, 28.6 (Pro(1)C_{β}), 27.7, 25.3–24.8 (ProC_{γ}), 23.2 (LysC_{γ}), 21.6, 19.3, 11.9. (Unlabeled chemical shifts are derived from the dehydrocholic acid moiety).

Supplementary Figures

NMR data of ligands

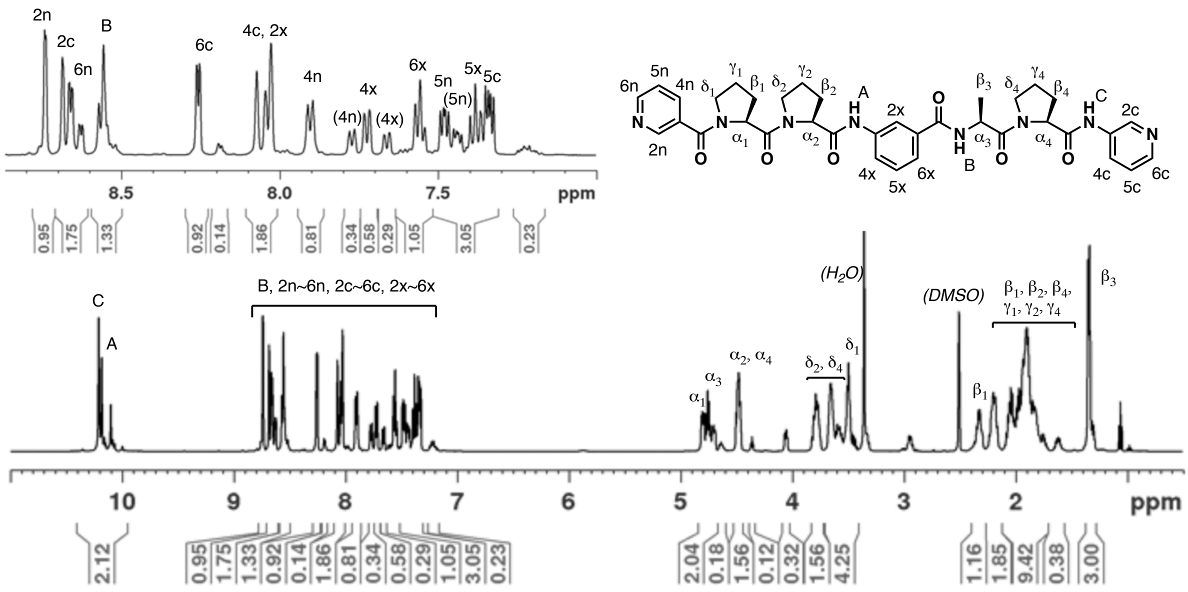

Supplementary Figure 1 |

^{1}H NMR spectrum (500 MHz, DMSO-d_6, 300 K) of **5**. Conformers coexist.

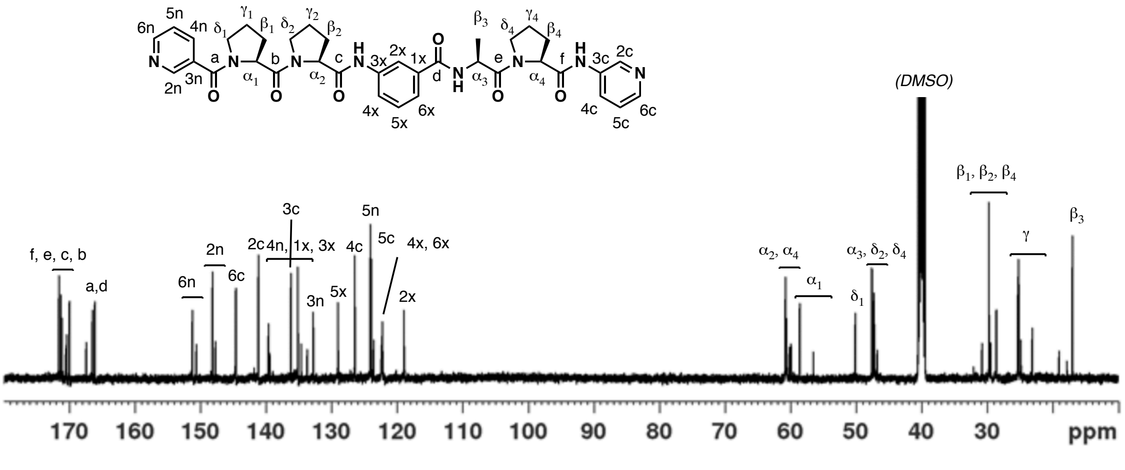

Supplementary Figure 2 |

^{13}C NMR spectrum (125 MHz, DMSO-d_6, 300 K) of **5**. Conformers coexist.

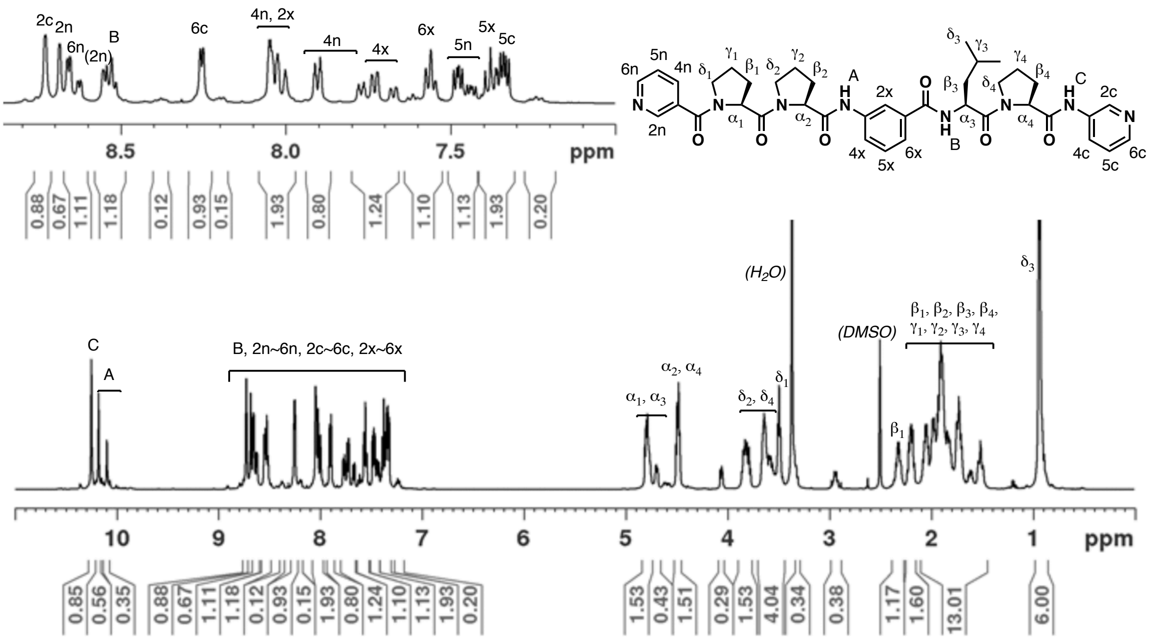

Supplementary Figure 3 |
^{1}H NMR spectrum (500 MHz, DMSO-d_6, 300 K) of **5L**. Conformers coexist.

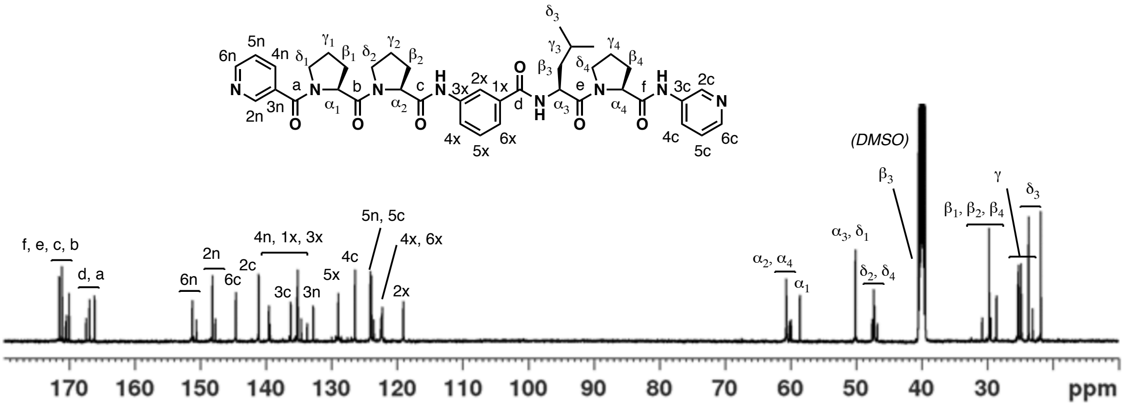

Supplementary Figure 4 |
^{13}C NMR spectrum (125 MHz, DMSO-d_6, 300 K) of **5L**. Conformers coexist.

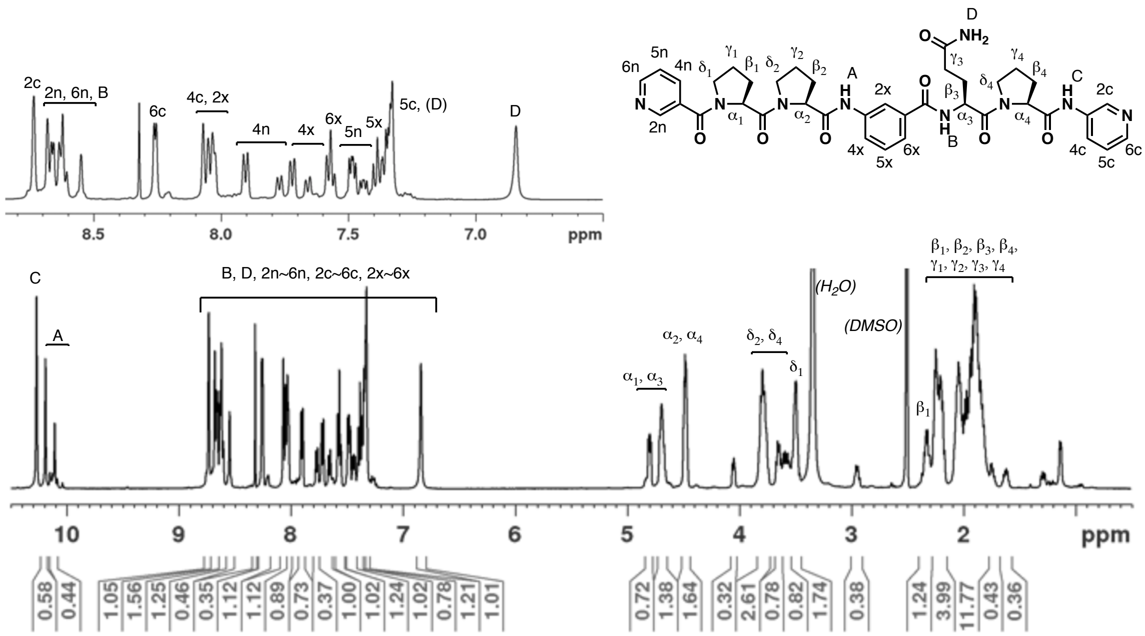

Supplementary Figure 5 |

^{1}H NMR spectrum (500 MHz, DMSO-d_6, 300 K) of **5Q**. Conformers coexist.

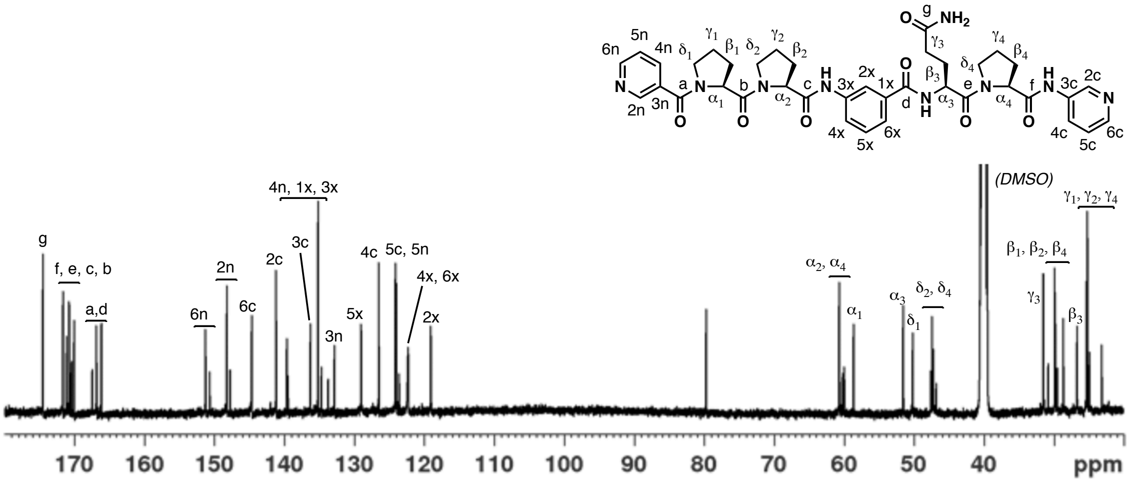

Supplementary Figure 6 |

^{13}C NMR spectrum (125 MHz, DMSO-d_6, 300 K) of **5Q**. Conformers coexist.

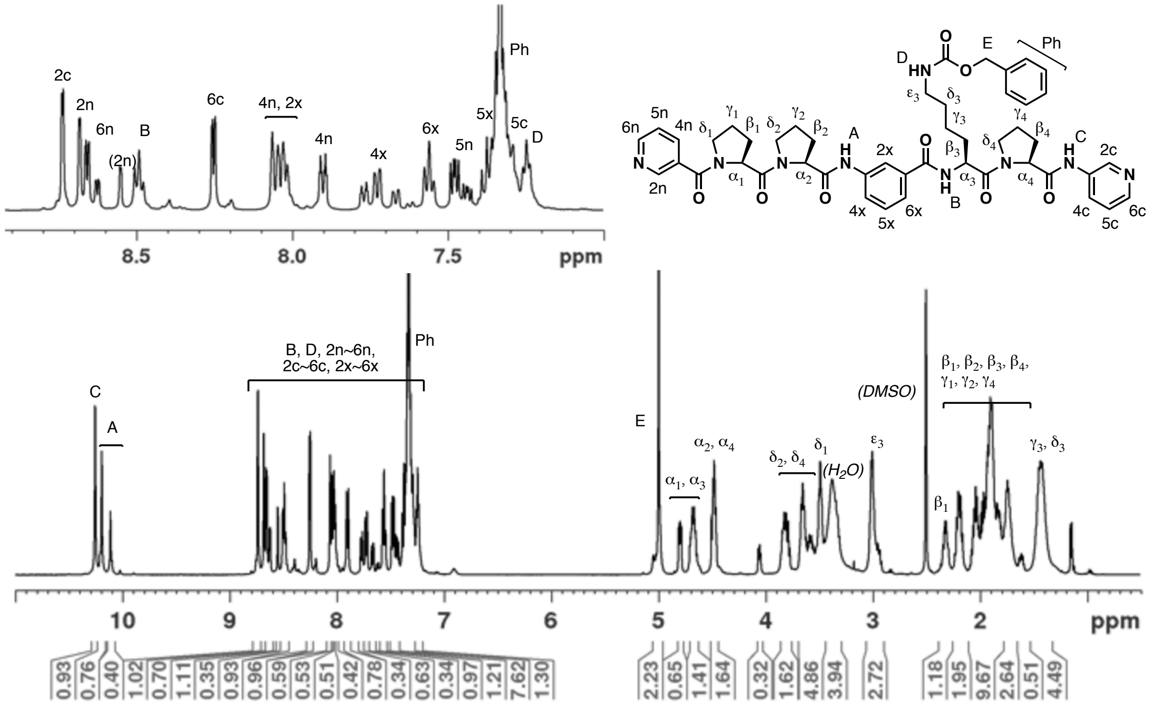

Supplementary Figure 7 |
^{1}H NMR spectrum (500 MHz, DMSO-d_6, 300 K) of **5K**. Conformers coexist.

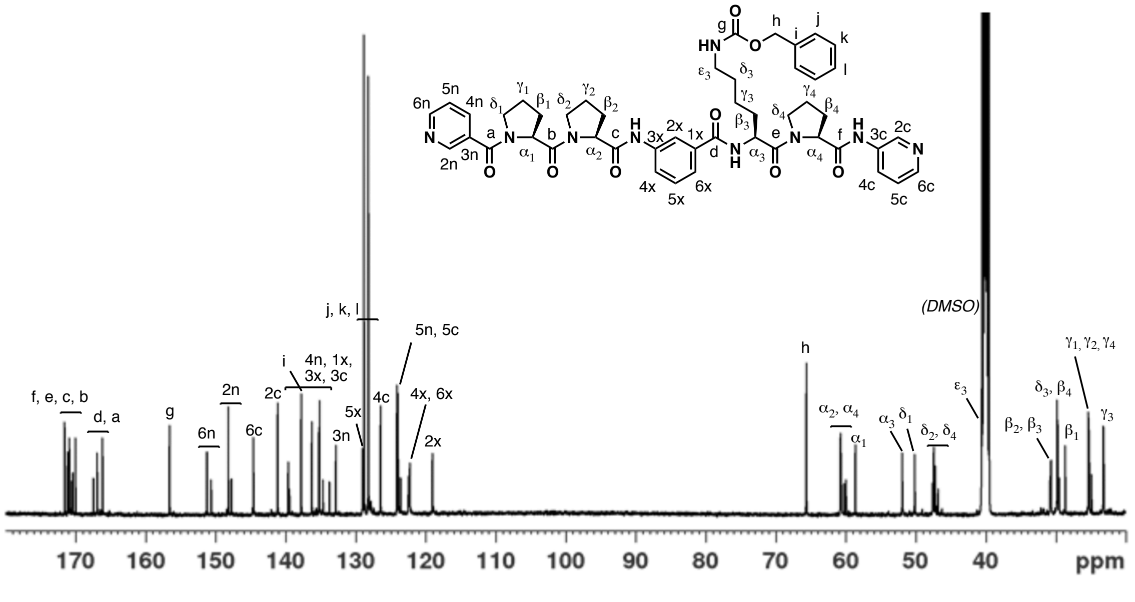

Supplementary Figure 8 |
^{13}C NMR spectrum (125 MHz, DMSO-d_6, 300 K) of **5K**. Conformers coexist.

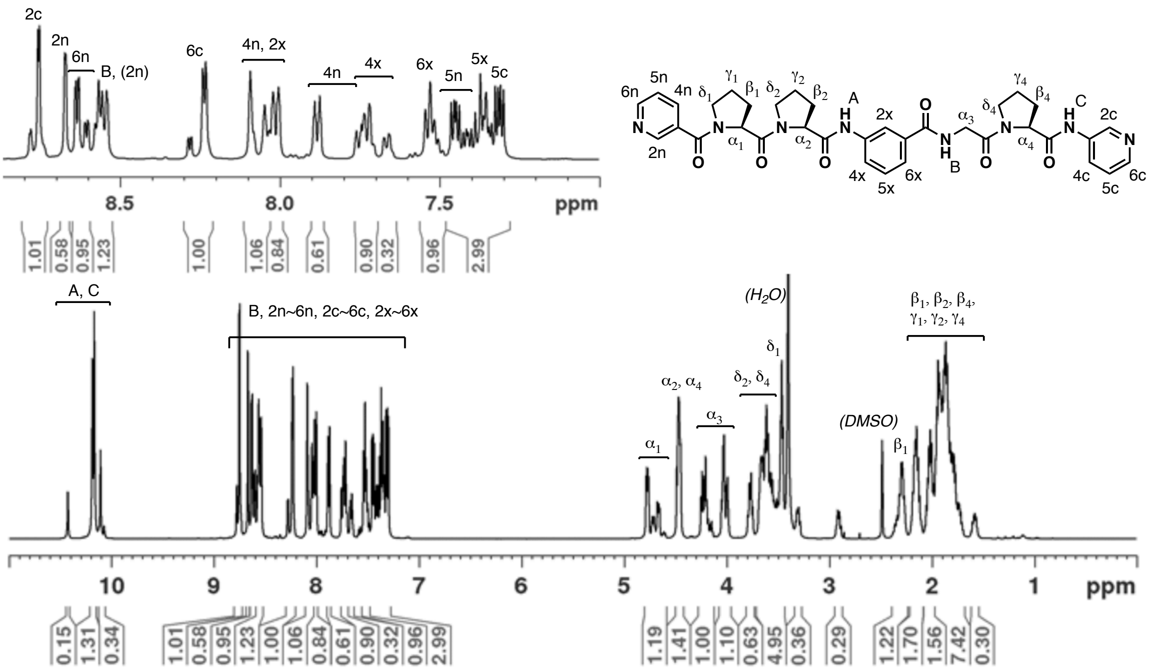

Supplementary Figure 9 |
^{1}H NMR spectrum (500 MHz, DMSO-d_6, 300 K) of **5G**. Conformers coexist.

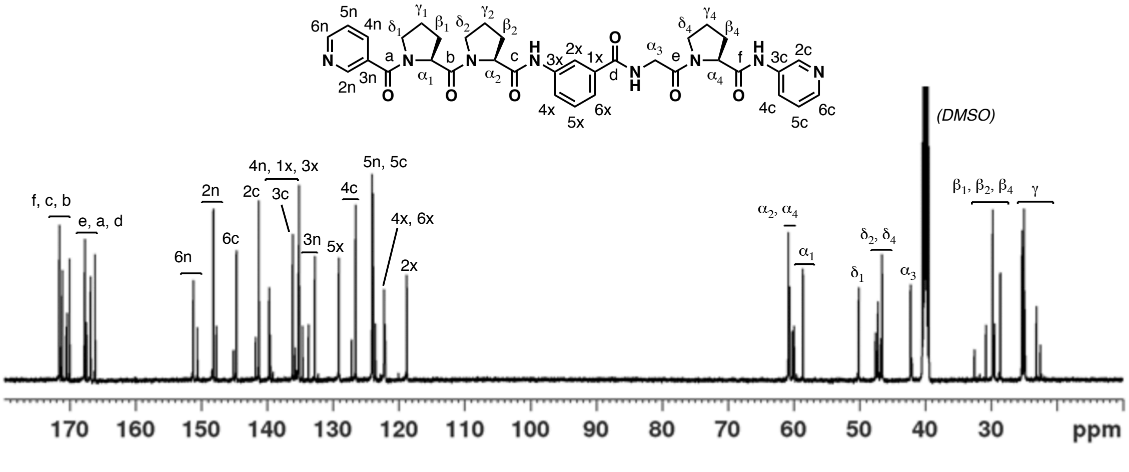

Supplementary Figure 10 |
^{13}C NMR spectrum (125 MHz, DMSO-d_6, 300 K) of **5G**. Conformers coexist.

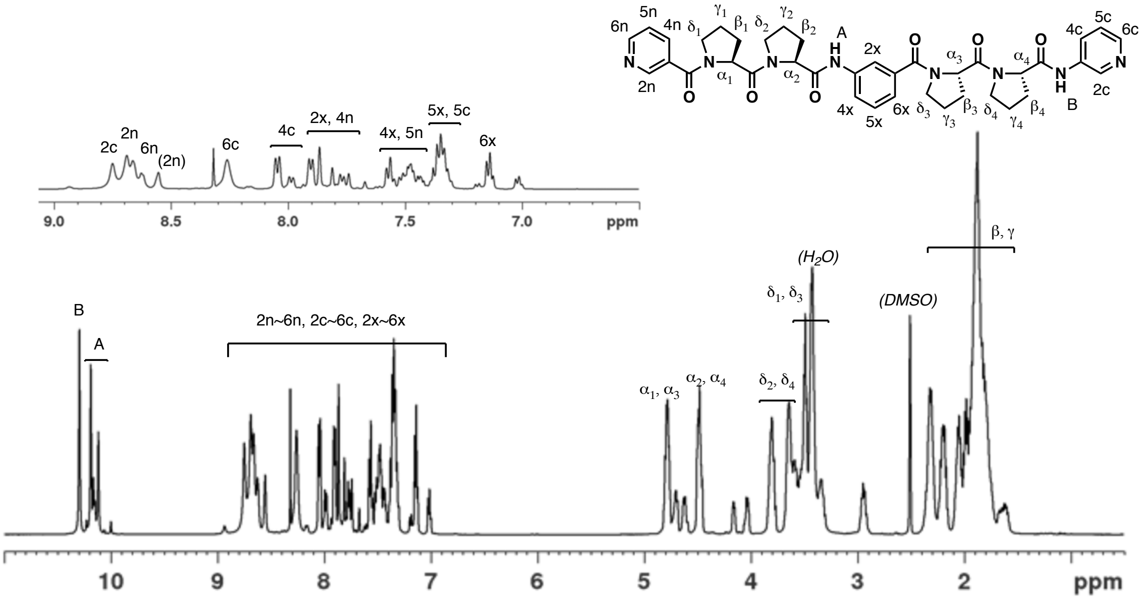

Supplementary Figure 11 |
^{1}H NMR spectrum (500 MHz, DMSO-d_6, 300 K) of **5P**. Conformers coexist.

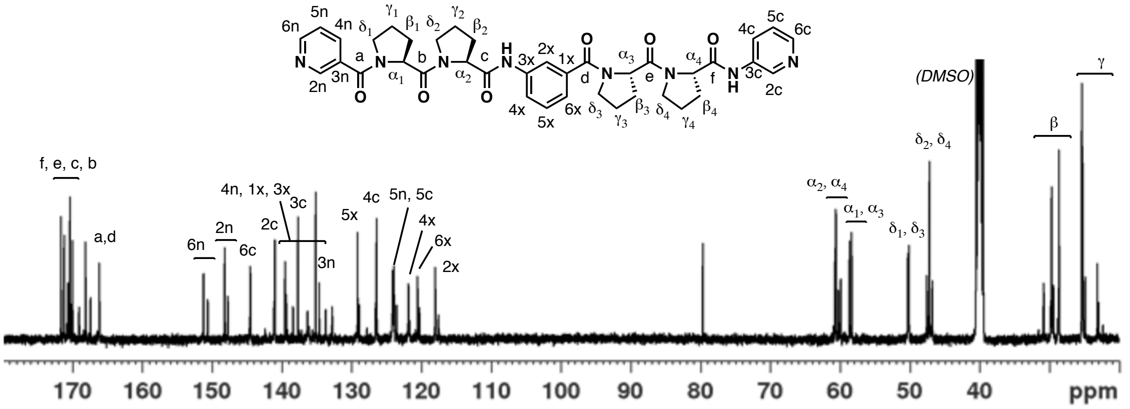

Supplementary Figure 12 |
^{13}C NMR spectrum (125 MHz, DMSO-d_6, 300 K) of **5P**. Conformers coexist.

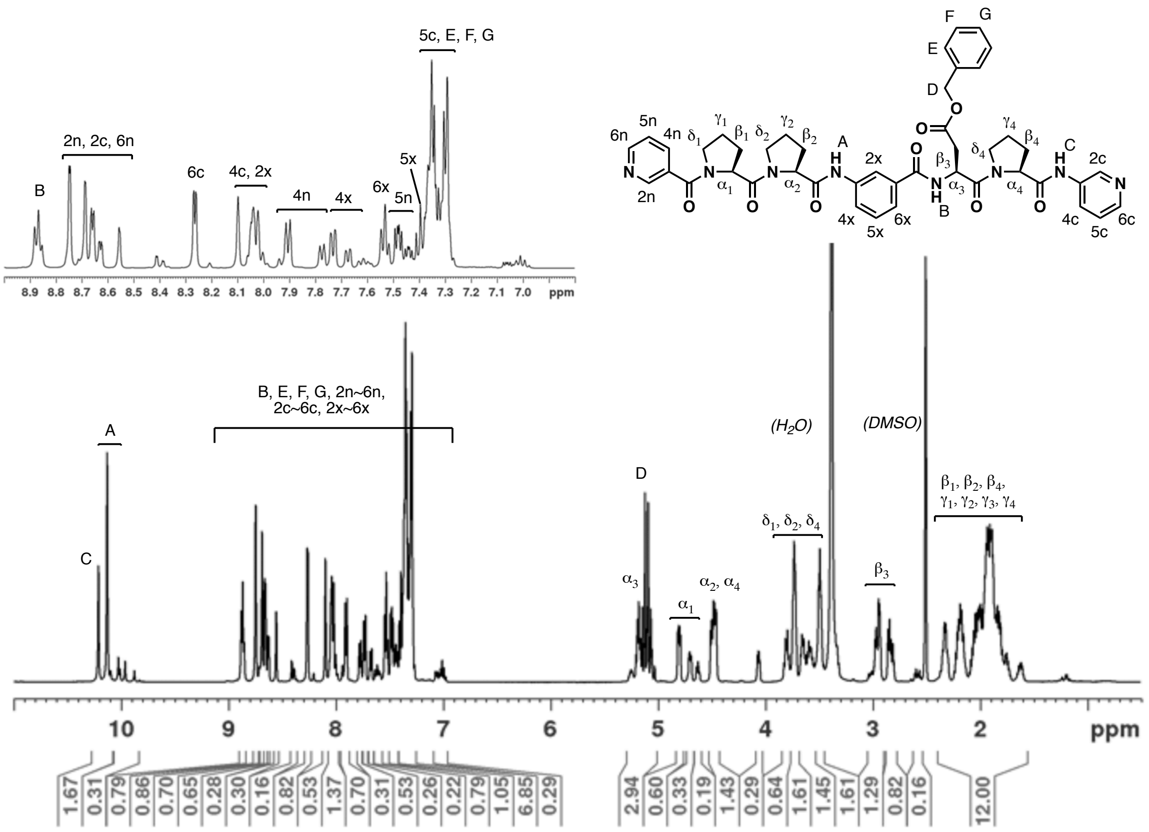

Supplementary Figure 13 |
^{1}H NMR spectrum (500 MHz, DMSO-d_6, 300 K) of **5D**. Conformers coexist.

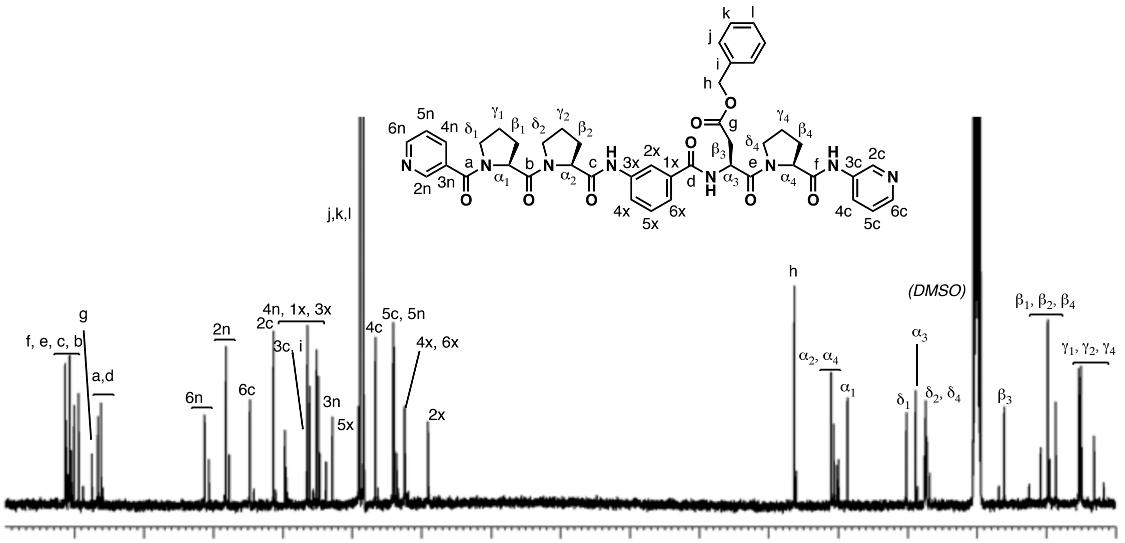

Supplementary Figure 14 |
^{13}C NMR spectrum (125 MHz, DMSO-d_6, 300 K) of **5D**. Conformers coexist.

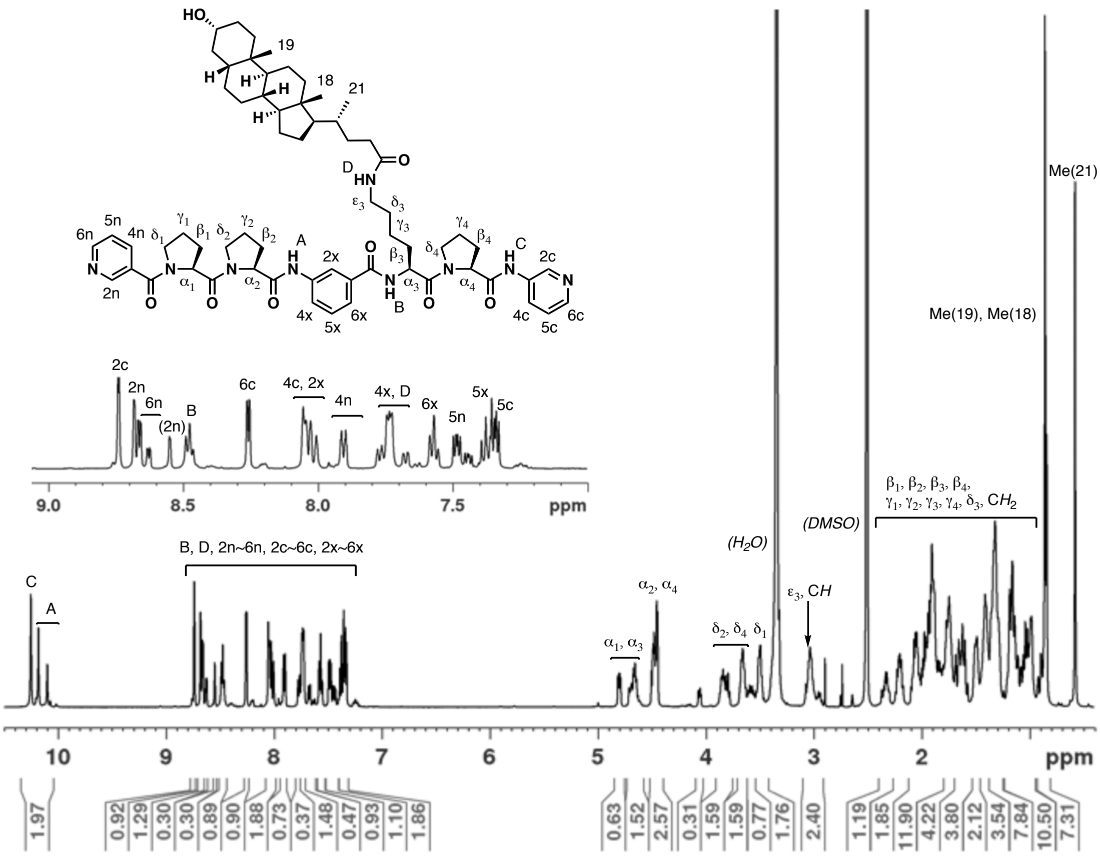

Supplementary Figure 15 |
^{1}H NMR spectrum (500 MHz, DMSO-d_6, 300 K) of **7**. Conformers coexist.

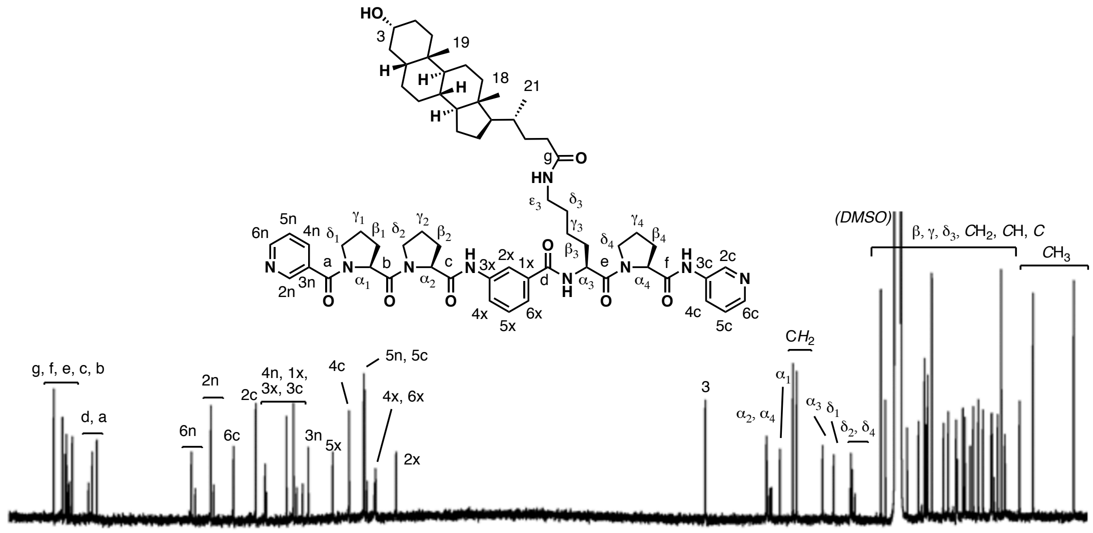

Supplementary Figure 16 |
^{13}C NMR spectrum (125 MHz, DMSO-d_6, 300 K) of **7**. Conformers coexist.

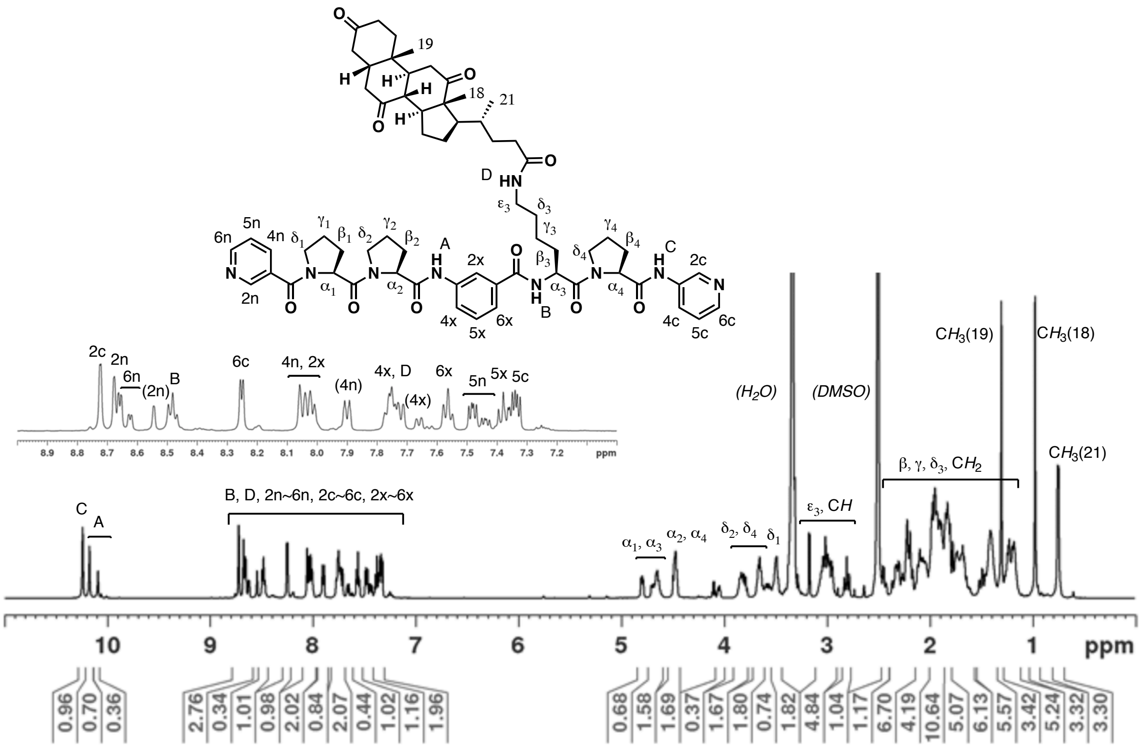

Supplementary Figure 17 |
^{1}H NMR spectrum (500 MHz, DMSO-d_6, 300 K) of **8**. Conformers coexist.

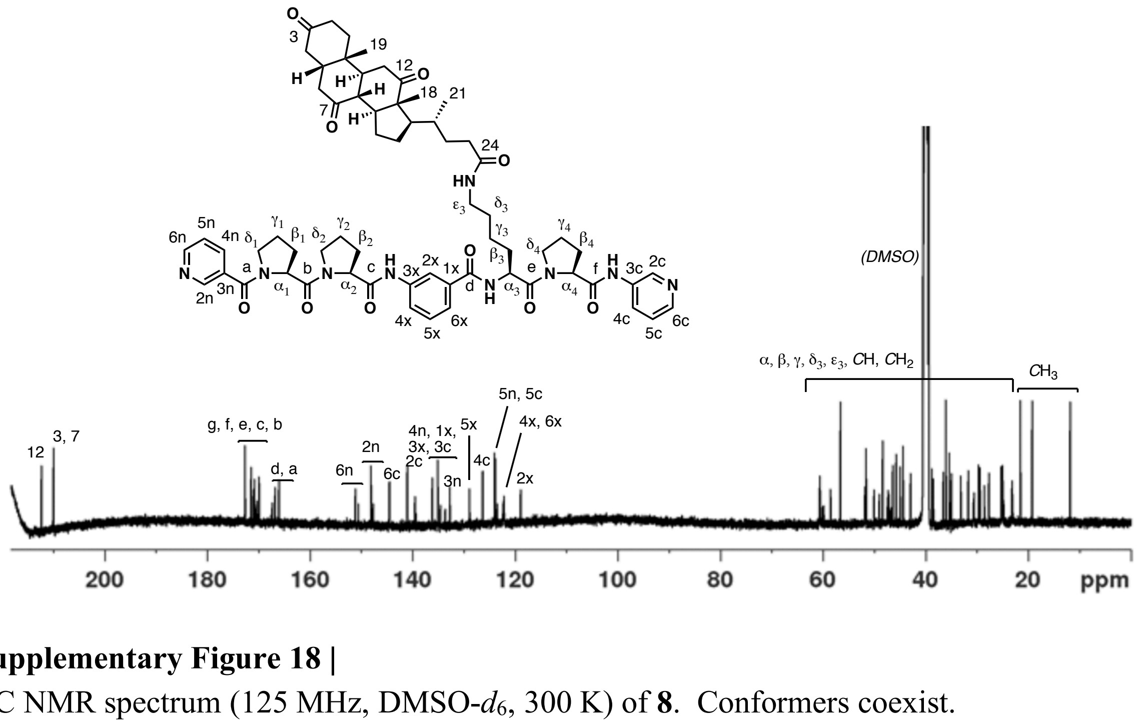

Supplementary Figure 18 |
^{13}C NMR spectrum (125 MHz, DMSO-d_6, 300 K) of **8**. Conformers coexist.

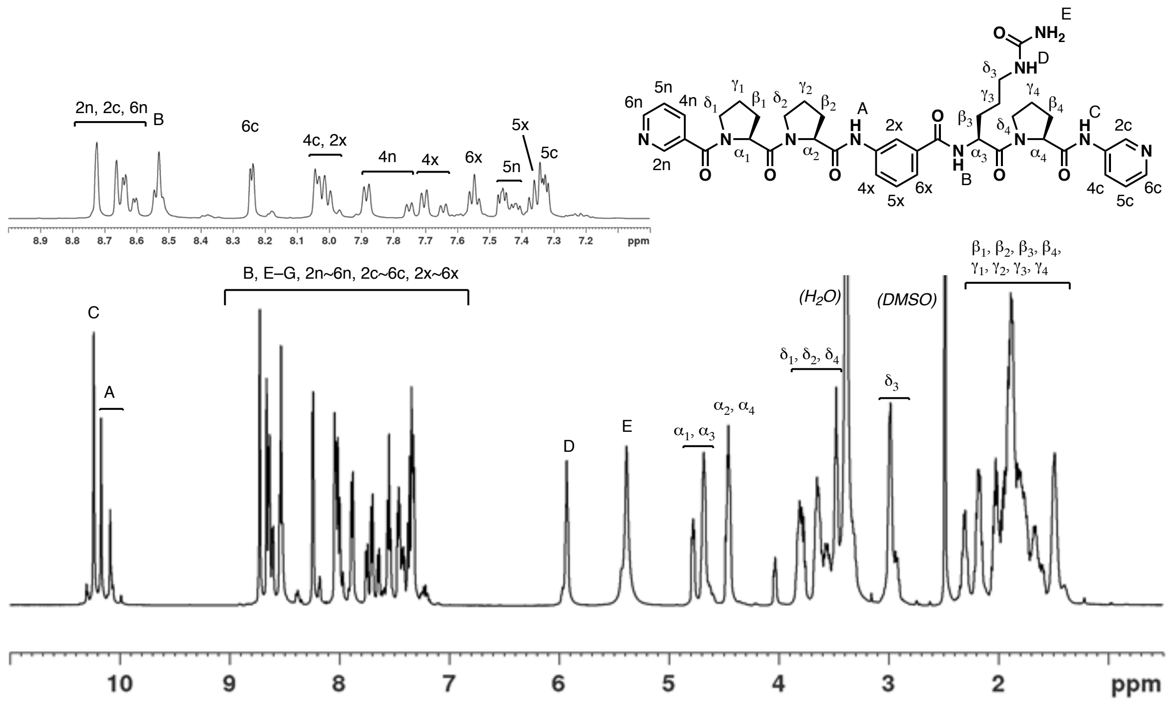

Supplementary Figure 19 |
^{1}H NMR spectrum (500 MHz, DMSO-d_6, 300 K) of **9**. Conformers coexist.

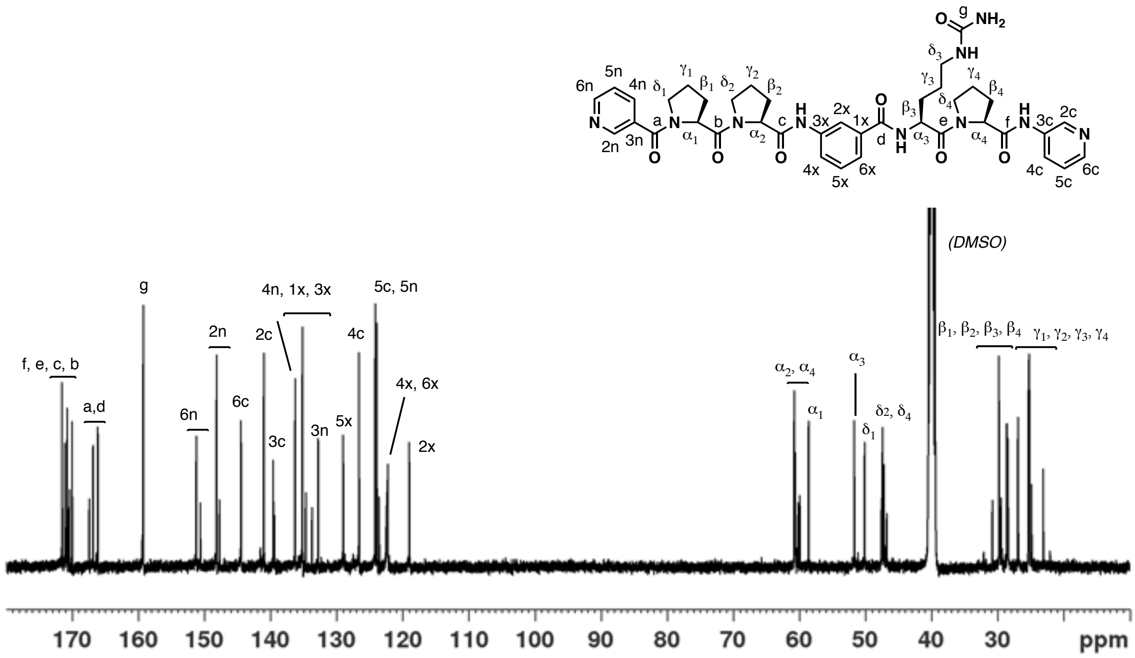

Supplementary Figure 20 |
^{13}C NMR spectrum (125 MHz, DMSO-d_6, 300 K) of **9**. Conformers coexist.

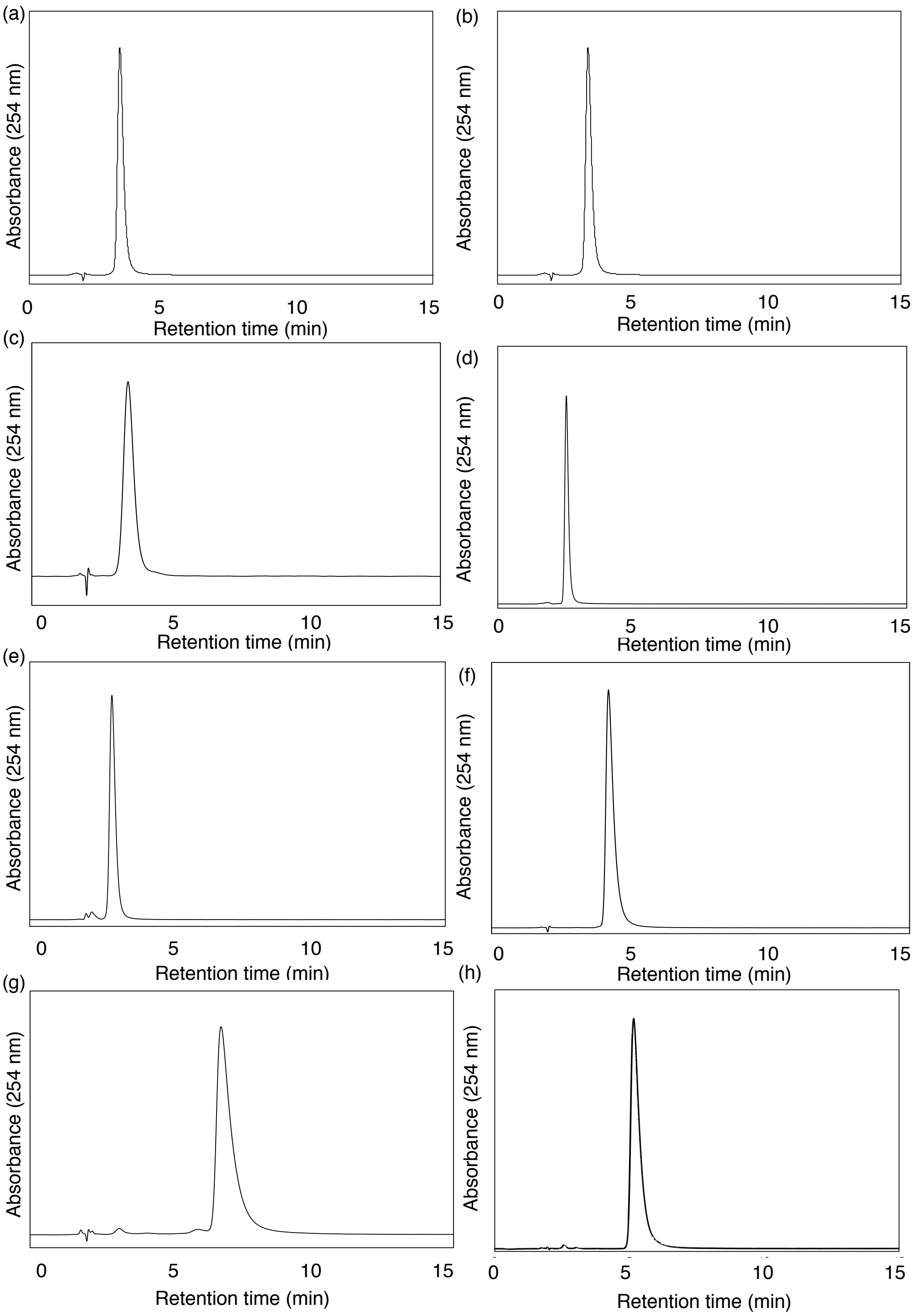

Supplementary Figure 21 |

Analytical HPLC profiles of (a) **5**, (b) **5L**, (c) **5Q**, (d) **5D**, (e) **5K**, (f) **5G**, (g) **5P**, and (h) **9** (eluent: (a-g) $CHCl_3$:EtOH = 90:10, (h) $CHCl_3$:EtOH = 85:15; flow rate: 1.0 mL/min, column: InertSustain NH2).

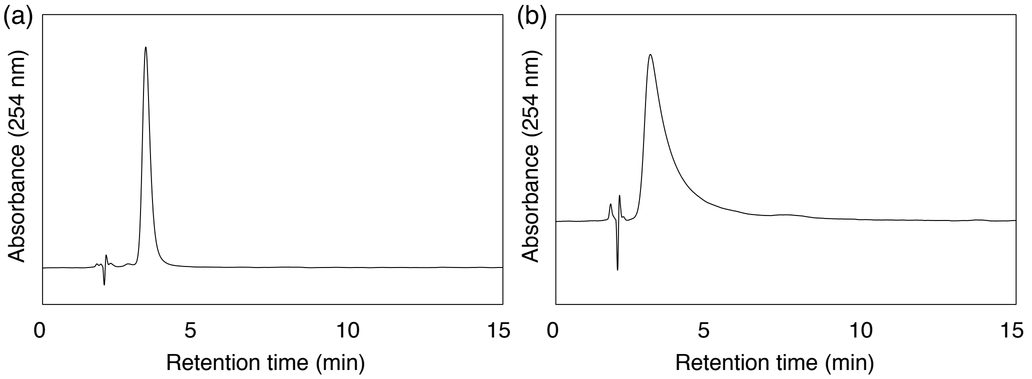

Supplementary Figure 22 |

Analytical HPLC profiles of (a) **7** and (b) **8** (eluent: $CHCl_3$:EtOH = 90:10; flow rate: 1.0 mL/min, column: InertSustain NH2).

NMR data of [6]catenane 6

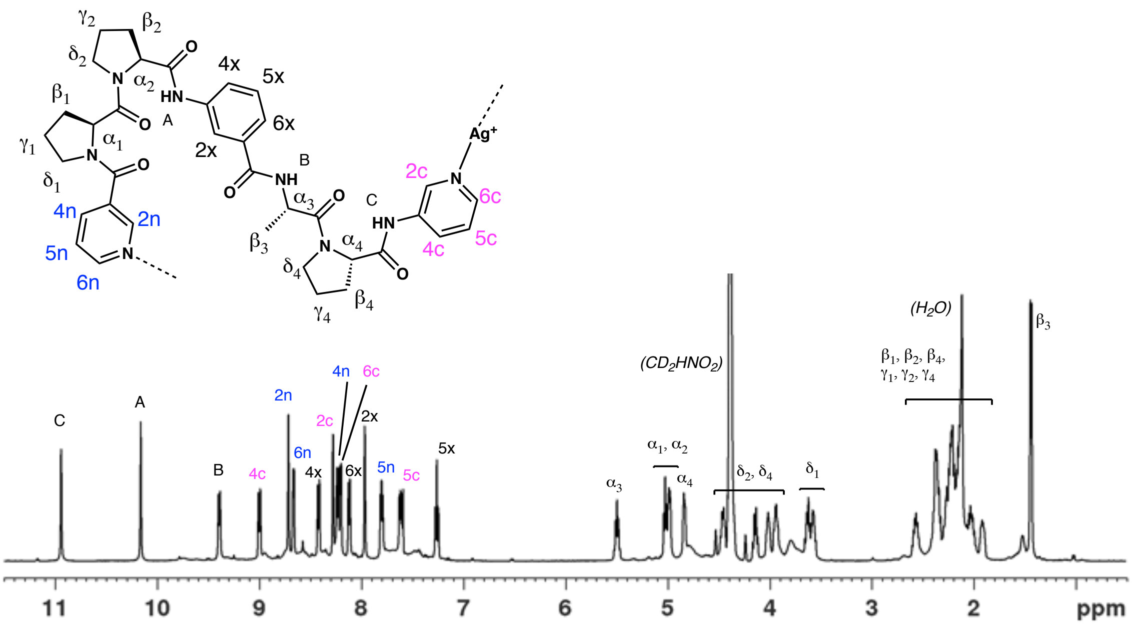

Supplementary Figure 23 |
^{1}H NMR spectrum of **6**•$(Tf_2N)_{24}$ (500 MHz, CD_3NO_2, 300 K, [**6**] = 0.83 mM).

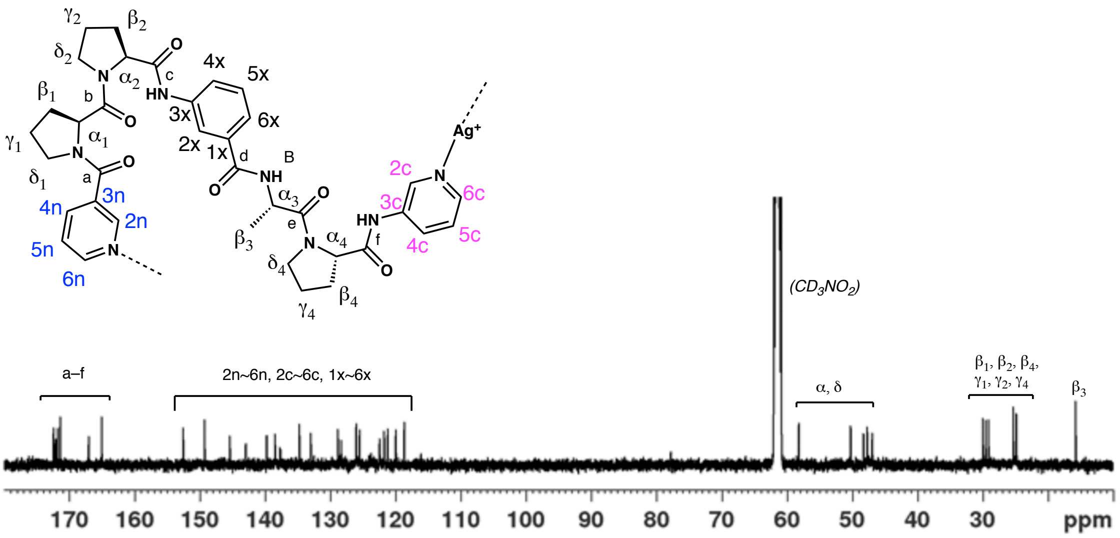

Supplementary Figure 24 |
^{13}C NMR spectrum of **6**•$(Tf_2N)_{24}$ (125 MHz, CD_3NO_2, 300 K, [**6**] = 0.83 mM).

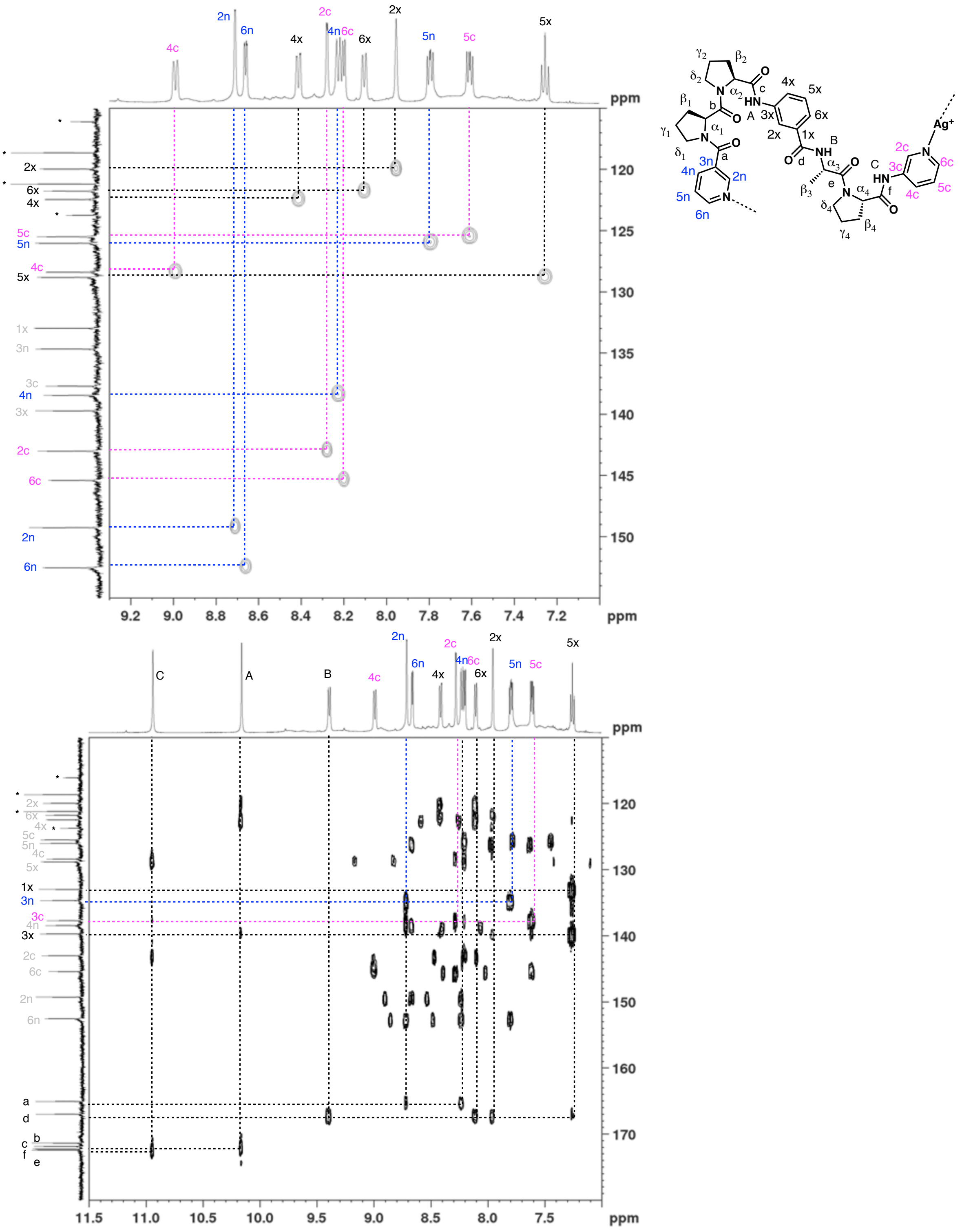

Supplementary Figure 25 |

^{1}H–^{13}C HSQC spectrum (top) and ^{1}H–^{13}C HMBC spectrum (bottom) of **6**•$(Tf_2N)_{24}$ (^{1}H: 500 MHz, ^{13}C: 125 MHz, CD_3NO_2, 300 K, [**6**] = 0.83 mM). Signals with asterisks derive from Tf_2N^-.

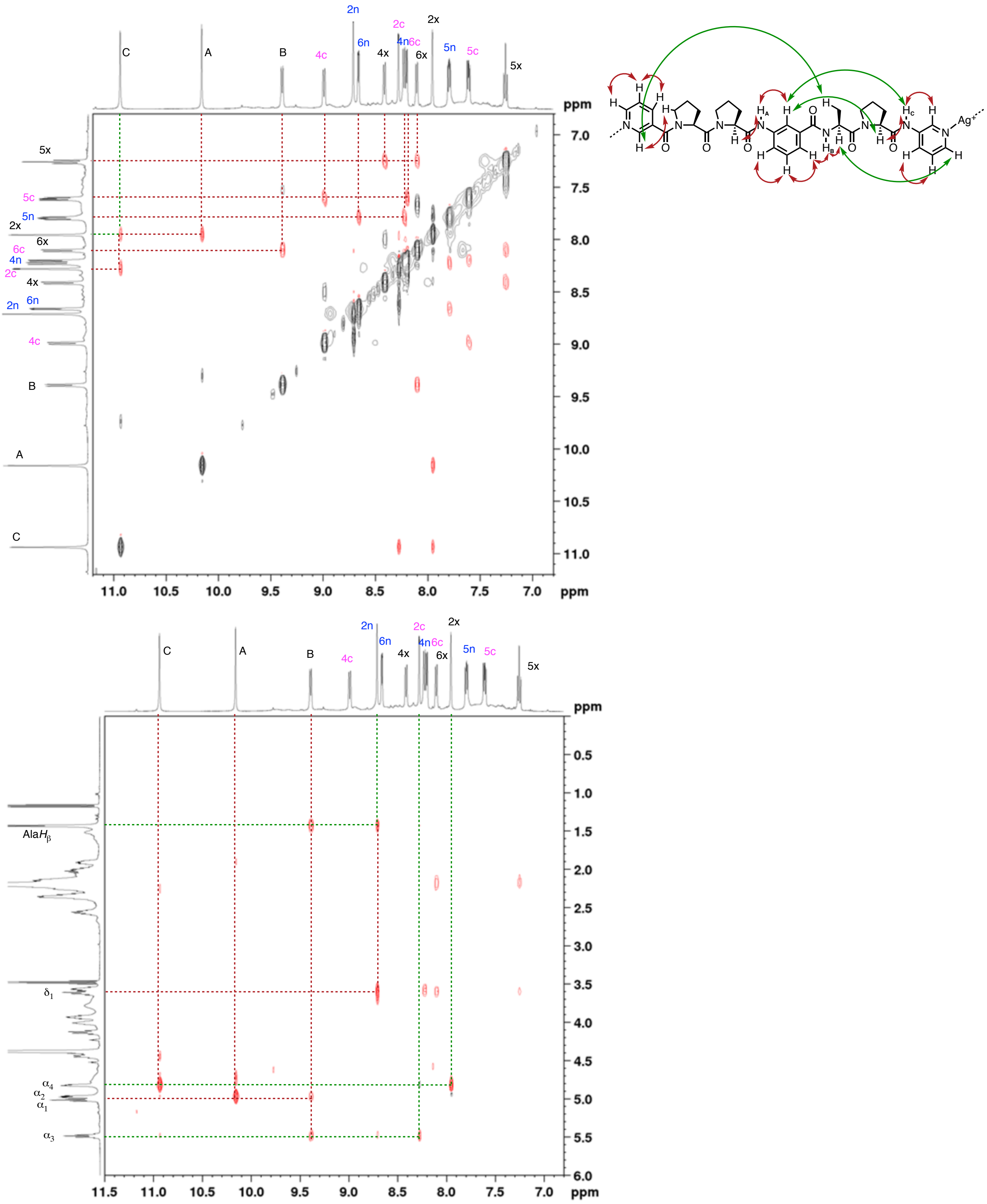

Supplementary Figure 26 |

^{1}H–^{1}H ROESY spectra (500 MHz, CD_3NO_2, 300 K) of **6**•$(Tf_2N)_{24}$ ([**6**] = 0.83 mM). The red and green arrows on the chemical structure indicate intra- and inter-strand's correlations, respectively.

NMR data of functionalised [6]catenanes

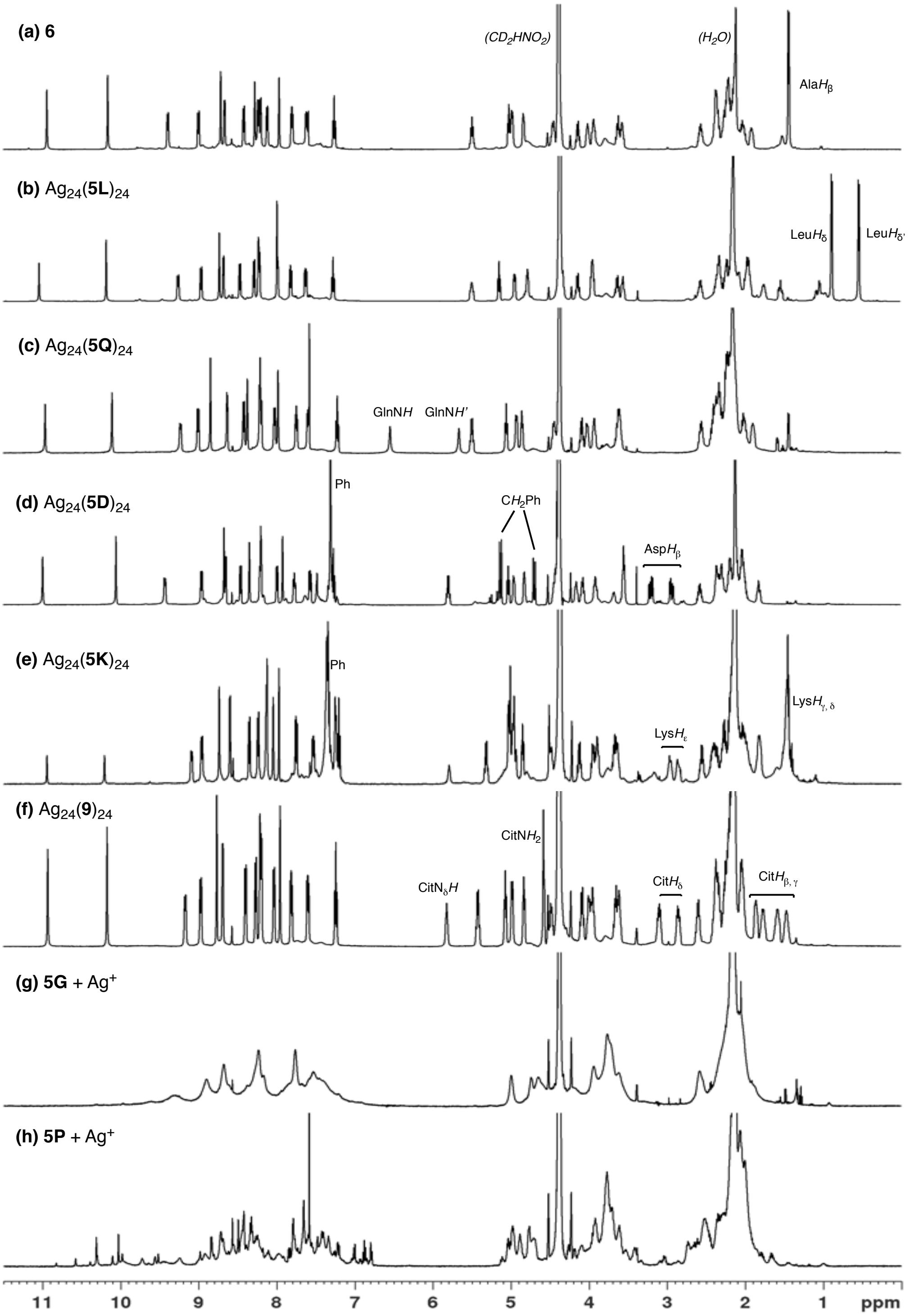

Supplementary Figure 27 |

Side chain scope for the [6]catenane formation: ^{1}H NMR spectra (500 MHz, CD_3NO_2, 300 K) of (a) **6**•$(Tf_2N)_{24}$, (b) $[Ag_{24}($**5L**$)_{24}](Tf_2N)_{24}$, (c) $[Ag_{24}($**5Q**$)_{24}](Tf_2N)_{24}$, (d) $[Ag_{24}($**5D**$)_{24}](PF_6)_{24}$, (e) $[Ag_{24}($**5K**$)_{24}](Tf_2N)_{24}$, (f) $[Ag_{24}($**9**$)_{24}](Tf_2N)_{24}$, (g) **5G** + $AgBF_4$, and (h) **5P** + $AgTf_2N$. Concentrations of both Ag^+ and each ligand are 10 mM, respectively. Broaden or non-convergent signals in (g) and (h) indicate unsuccessful formation of [6]catenanes.

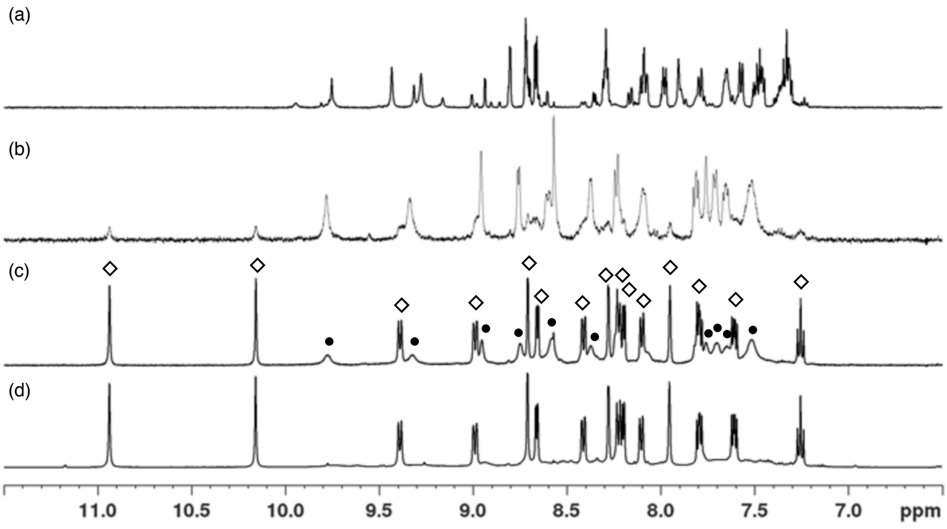

Supplementary Figure 28 |

Concentration dependence on the [6]catenane formation: ^{1}H NMR spectra (500 MHz, CD_3NO_2, 300 K) of (a) ligand **5**, (b) [**6**] = 0.042 mM, (c) [**6**] = 0.21 mM, and (d) [**6**] = 0.42 mM. In case of [**6**] ≥ 0.42 mM, signals of the [6]catenane were quantitatively observed (square). In contrast, signals derived from smaller subcomponents exist at lower concentrations (black dots). $AgTf_2N$ was used for these complexation reactions.

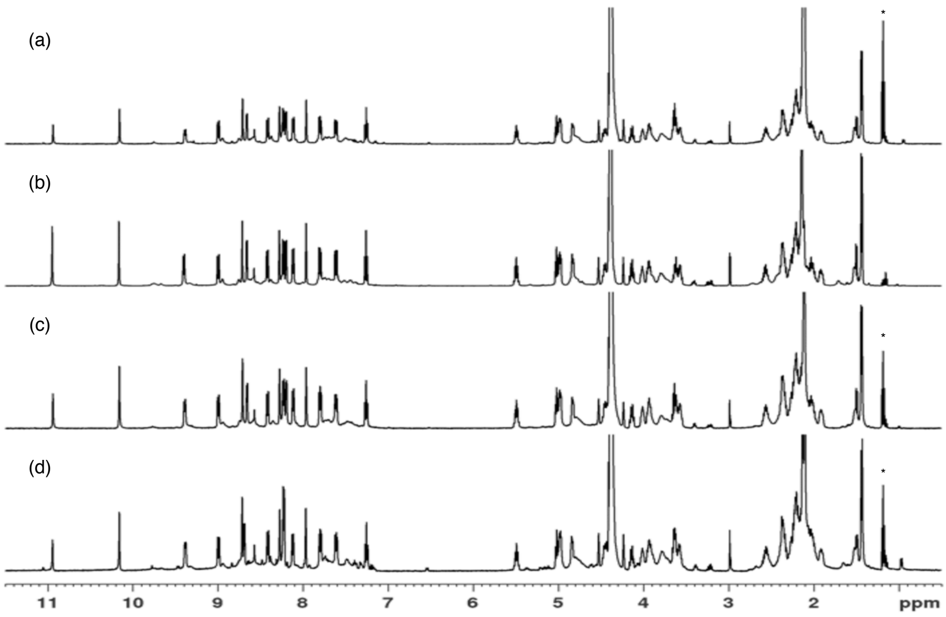

Supplementary Figure 29 |

Counter anion dependence on the [6]catenane formation: ^{1}H NMR spectra (500 MHz, CD_3NO_2, 300 K) of (a) **6**•$(BF_4)_{24}$, (b) **6**•$(Tf_2N)_{24}$, (c) **6**•$(PF_6)_{24}$, and (d) **6**•$(OTf)_{24}$ ([**6**] = 0.42 mM). Formation of the [6]catenane proceeds smoothly by using these counter anions. Signals with asterisks derive from a solvent.

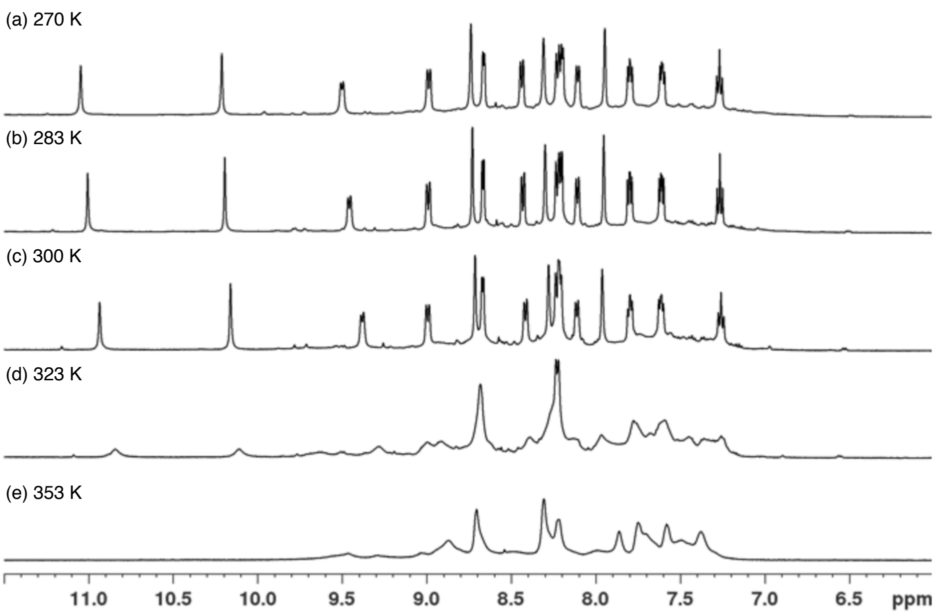

Supplementary Figure 30 |

Thermal stability of **6**: ^{1}H NMR spectra (500 MHz, CD_3NO_2) of **6**•$(Tf_2N)_{24}$ measured at (a) 270 K, (b) 283 K, (c) 300 K, (d) 323 K, and (e) 353 K. At higher temperature than 323 K, it was suggested that the [6]catenane framework disassembled into an undeterminable species.

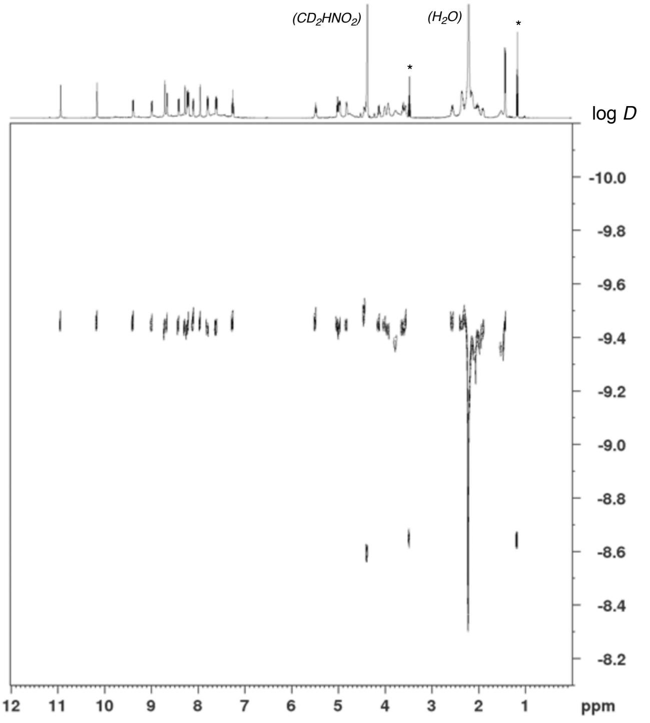

Supplementary Figure 31 |

^{1}H DOSY NMR spectrum (500 MHz, CD_3NO_2, 300 K) of **6**•$(Tf_2N)_{24}$ ([**6**] = 0.83 mM, D = 3.4×10^{-10} m^2•s^{-1}). The signals with asterisks derive from a solvent.

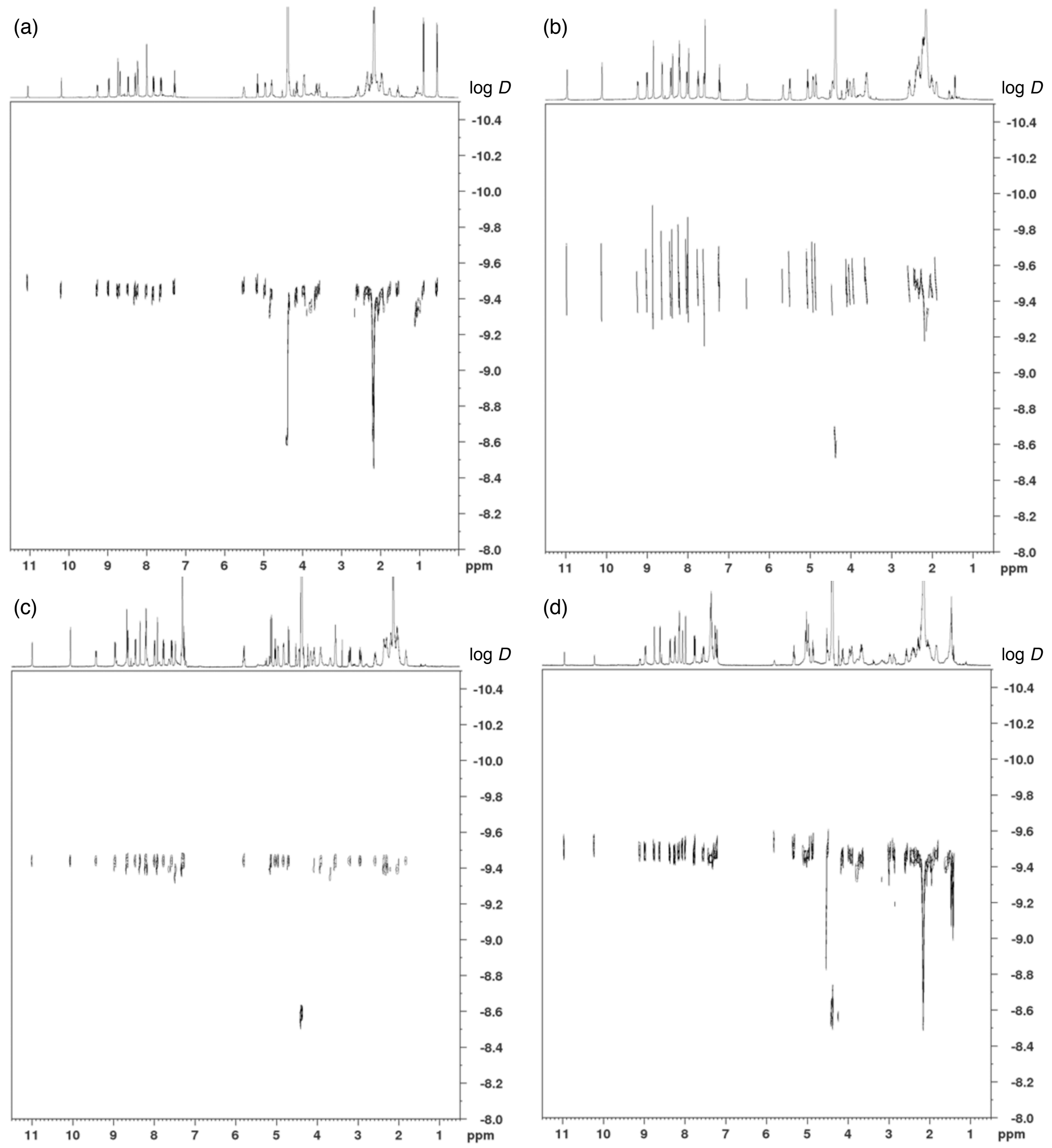

Supplementary Figure 32 |

^{1}H DOSY NMR spectra (500 MHz, CD_3NO_2, 300 K) of (a) $[Ag_{24}(\mathbf{5L})_{24}](Tf_2N)_{24}$ (D = 3.5×10^{-10} $m^2\bullet s^{-1}$), (b) $[Ag_{24}(\mathbf{5Q})_{24}](Tf_2N)_{24}$ (D = 3.2×10^{-10} $m^2\bullet s^{-1}$), (c) $[Ag_{24}(\mathbf{5D})_{24}](PF_6)_{24}$ (D = 3.5×10^{-10} $m^2\bullet s^{-1}$), and (d) $[Ag_{24}(\mathbf{5K})_{24}](Tf_2N)_{24}$ (D = 3.4×10^{-10} $m^2\bullet s^{-1}$). The concentration of each complex is 0.42 mM.

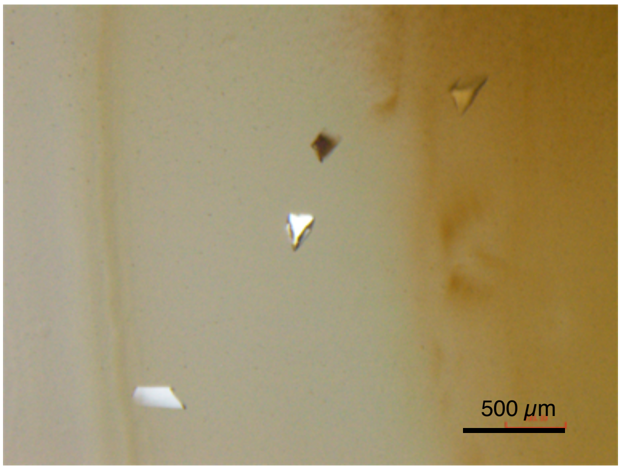

Supplementary Figure 33 |
Crystal photo of **6**•$(Tf_2N)_{24}$.

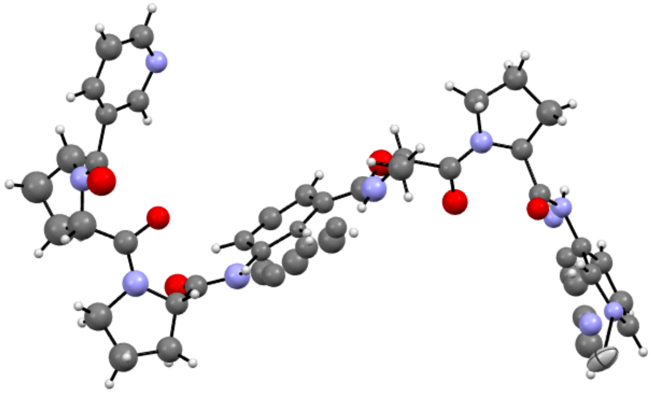

Supplementary Figure 34 |

ORTEP drawings (30% probability ellipsoids) of the asymmetric unit structure of **6** (crystal structure A). Solvents and counter anions were omitted for clarity. The *m*-phenylene spacer and the *C*-terminal pyridine were disordered in two positions (50% occupancies), respectively.

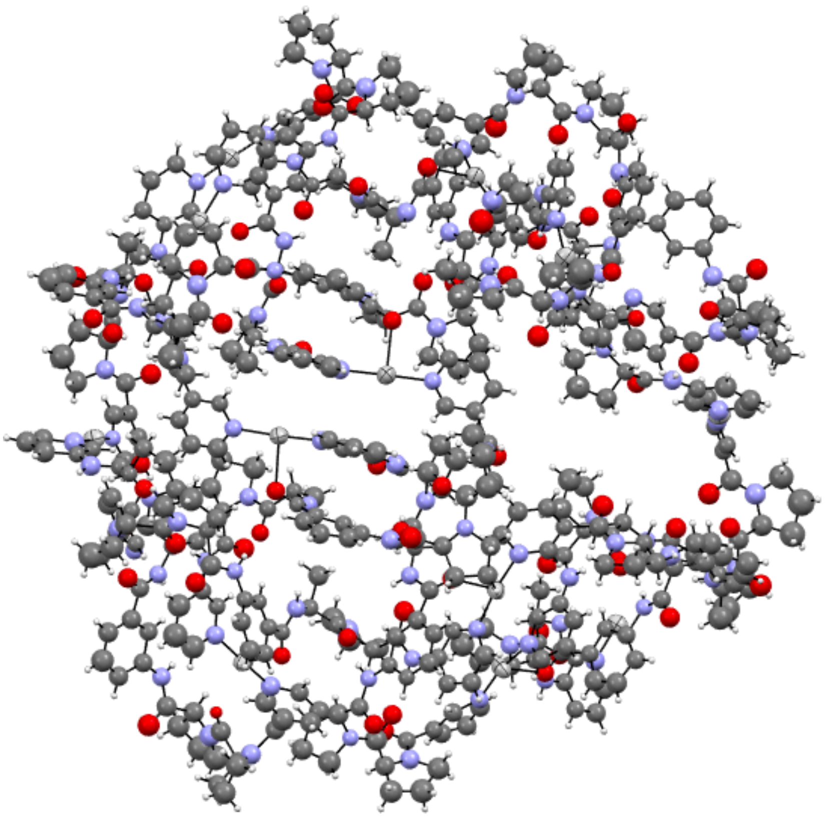

Supplementary Figure 35 |

ORTEP drawings (30% probability ellipsoids) of the asymmetric unit structure of **6** (crystal structure B). Solvents and counter anions were omitted for clarity.

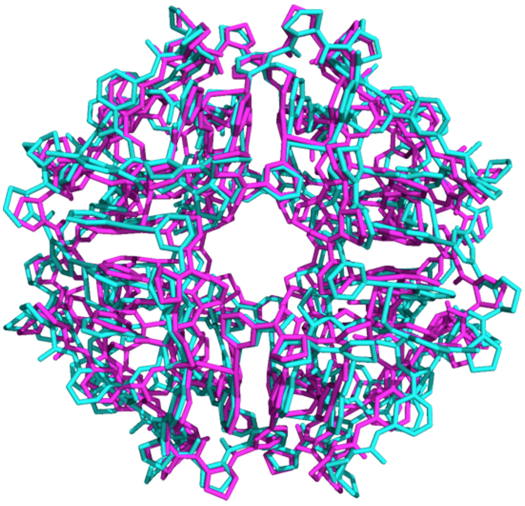

Supplementary Figure 36 |

Overlay of two crystal coordinates of crystal structure A (magenta) and B (cyan). RMS of 24 Ag atoms = 0.936 Å. Solvents and counter anions were omitted for clarity.

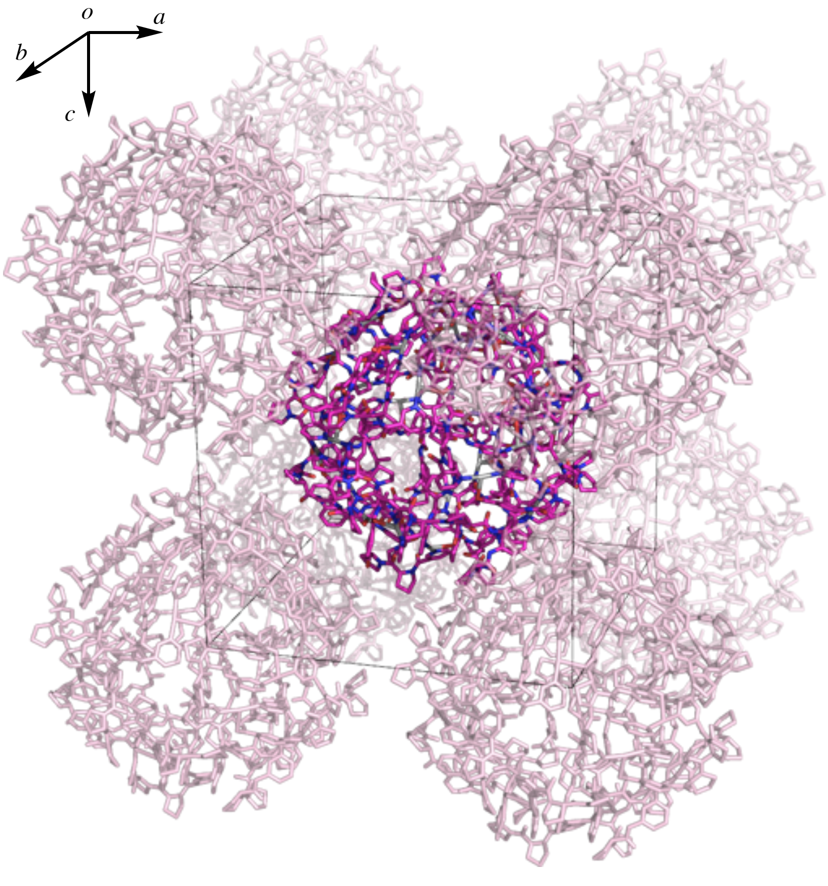

Supplementary Figure 37 |

Crystal packing of **6** (crystal structure A). Solvents and counter anions were omitted for clarity.

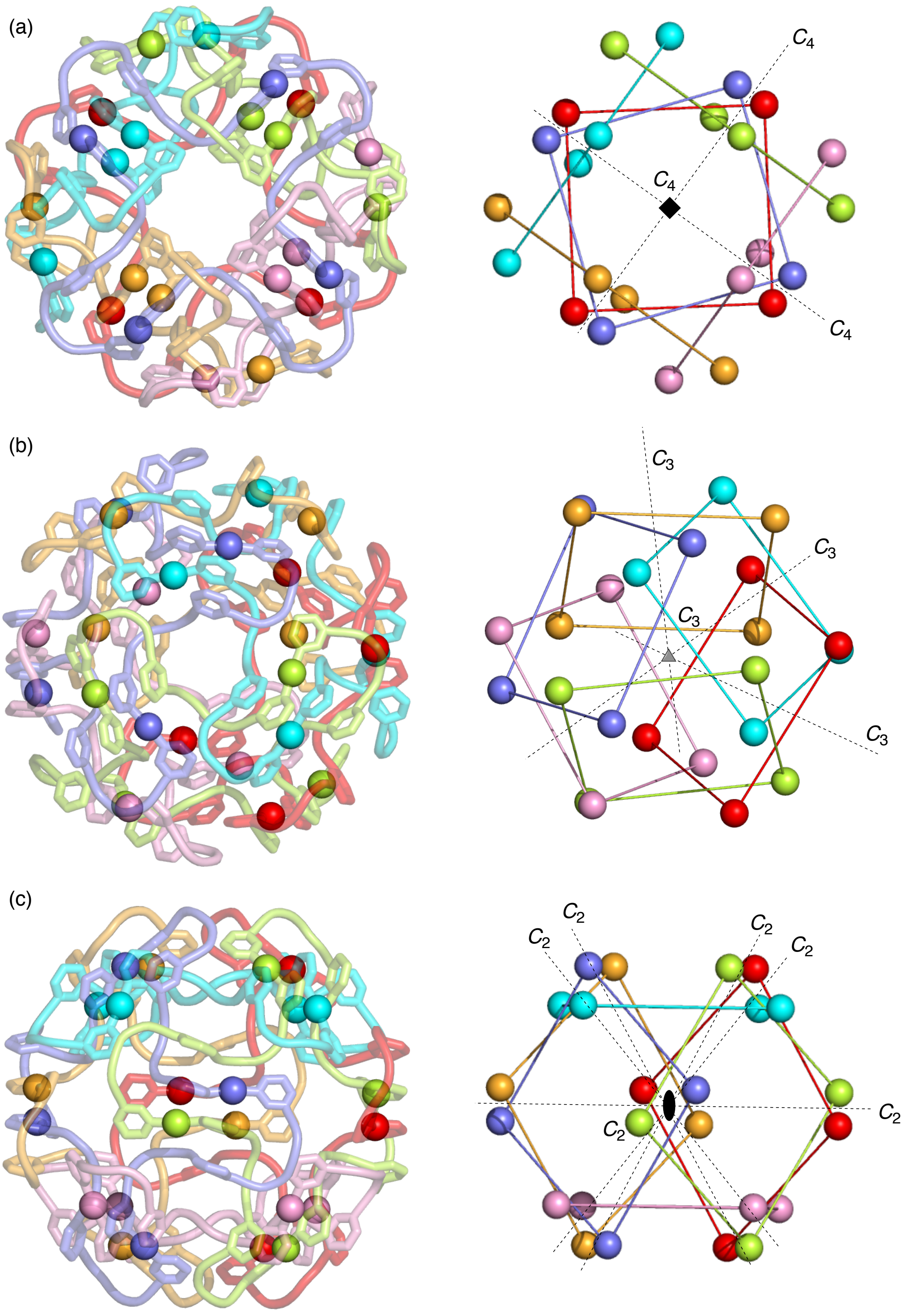

Supplementary Figure 38 |

Views along (a) the C_4 axis, (b) the C_3 axis, and (c) the C_2 axis of the crystal structure of **6** (crystal structure A). The geometries of Ag^+ positions were extracted to right. Each metal–peptide macrocycle was colour-coded.

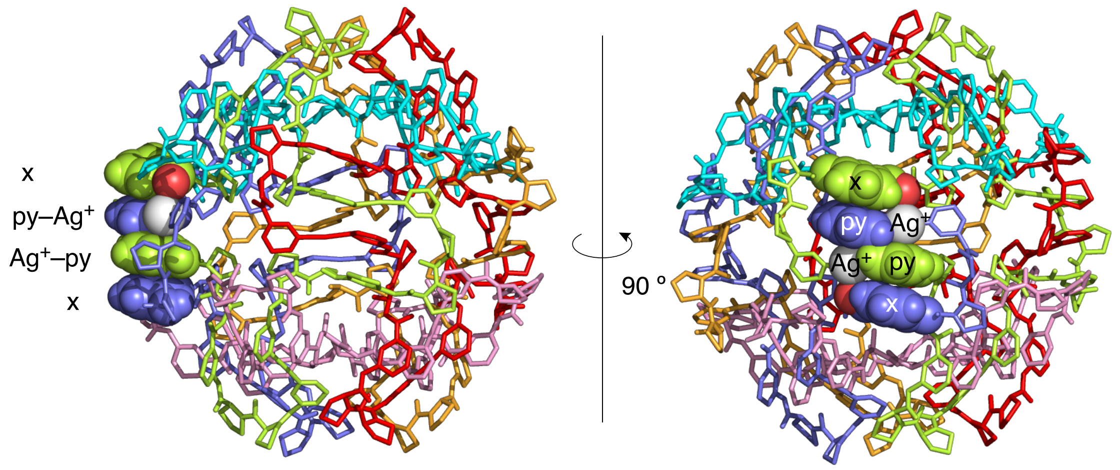

Supplementary Figure 39 |

A quadruple stacking structure of x and py–Ag^+ observed in the crystal of **6** (one set of Ag^+, py and x groups are highlighted in space-filling representation). Carbonyl oxygen of x spacer was also weakly coordinated to Ag^+ (Ag^+•••O distance: ~ 2.6 Å).

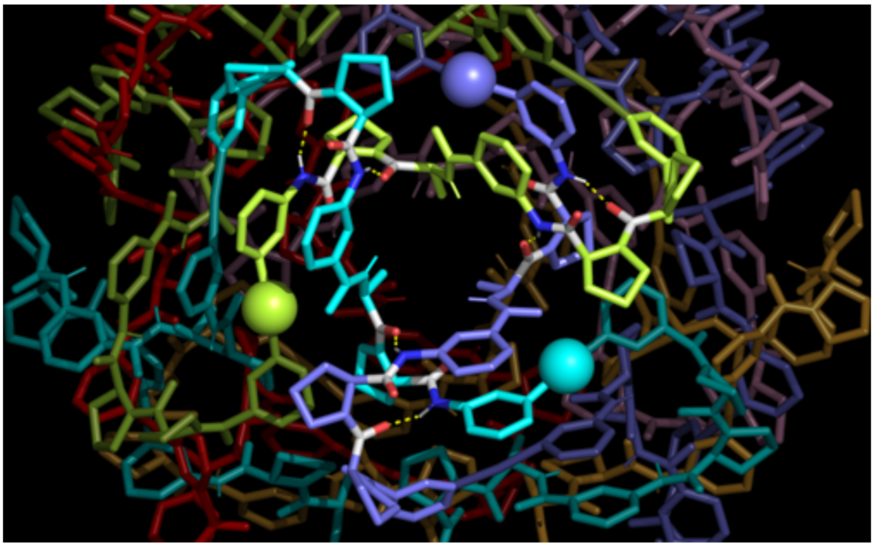

Supplementary Figure 40 |

Inter-strand H-bonds observed in a three-way junction unit in **6**. Yellow dash lines indicate H-bonds.

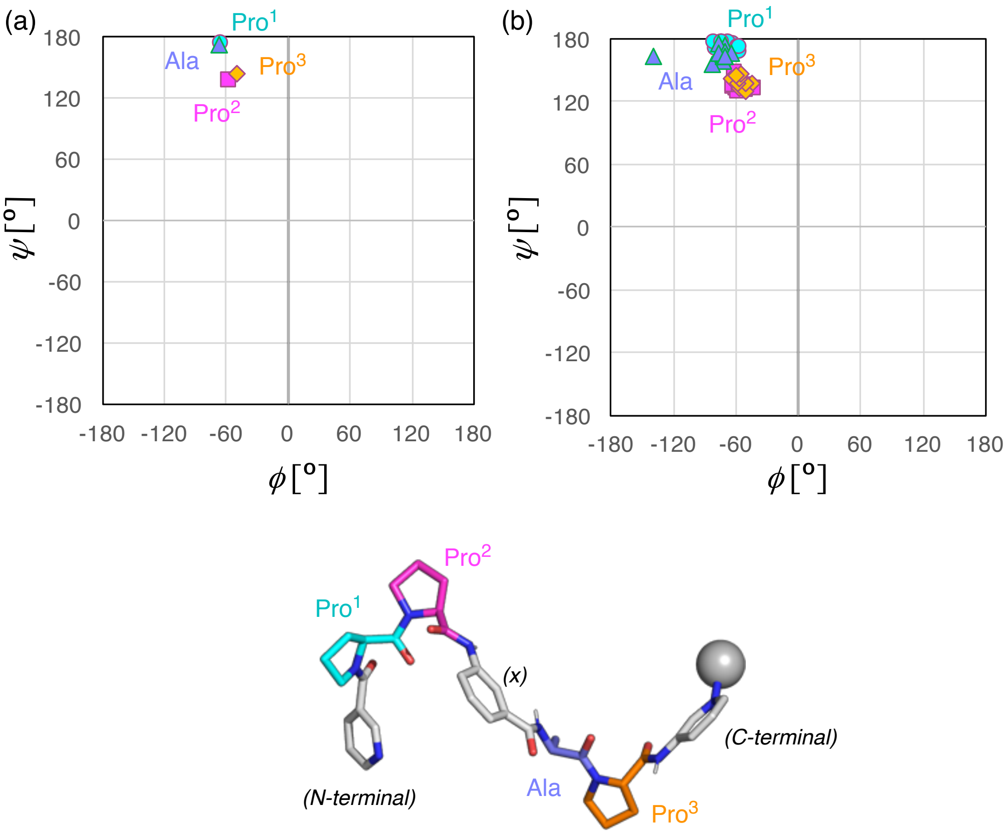

Supplementary Figure 41 |

Ramachandran plots of (a) **6** of crystal structure A and (b) that of crystal structure B. Crystallographically independent one pentapeptide ligand was plotted in (a), and twelve pentapeptide ligands in (b).

(a) [6]catenane **6**•$(PF_6)_{24}$

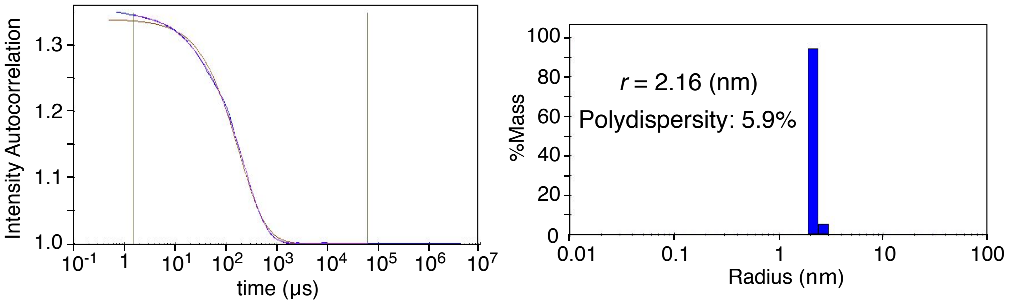

(b) unconvergent species **5G** + AgOTf

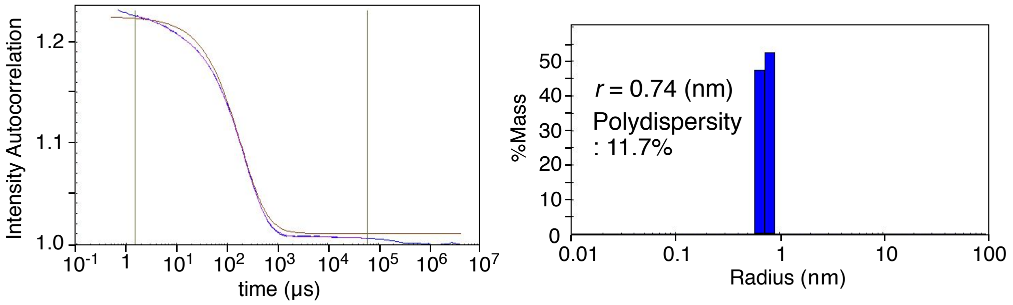

(c) [6]catenane $[Ag_{24}(\mathbf{5L})_{24}](OTf)_{24}$

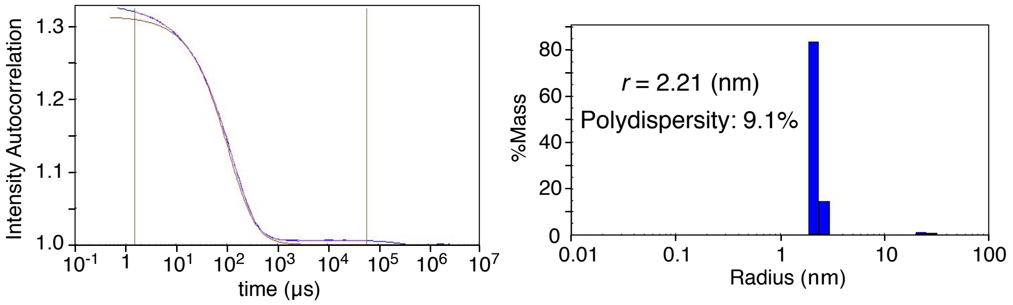

Supplementary Figure 42 |

DLS data of (a) **6**•$(PF_6)_{24}$, (b) **5G** + Ag^+, (c) $[Ag_{24}(\mathbf{5L})_{24}](OTf)_{24}$, (d) $[Ag_{24}(\mathbf{5Q})_{24}](OTf)_{24}$, (e) $[Ag_{24}(\mathbf{5D})_{24}](PF_6)_{24}$, and (f) $[Ag_{24}(\mathbf{5K})_{24}](OTf)_{24}$ (*left*: correlation graphs, *right*: regularization graphs, measured at 20 ºC).

(d) [6]catenane $[Ag_{24}(\mathbf{5Q})_{24}](OTf)_{24}$

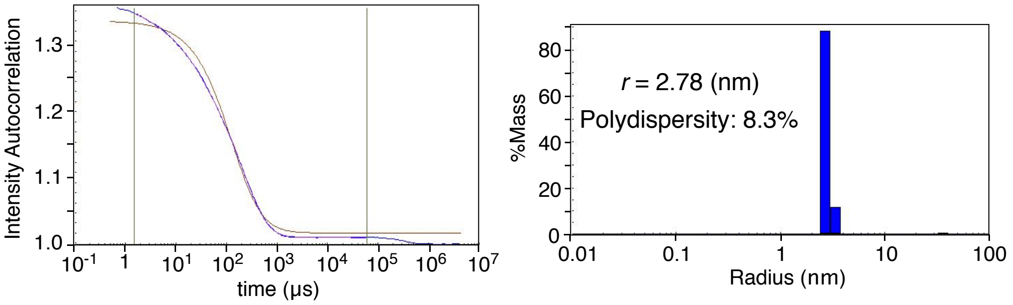

(e) [6]catenane $[Ag_{24}(\mathbf{5D})_{24}](PF_6)_{24}$

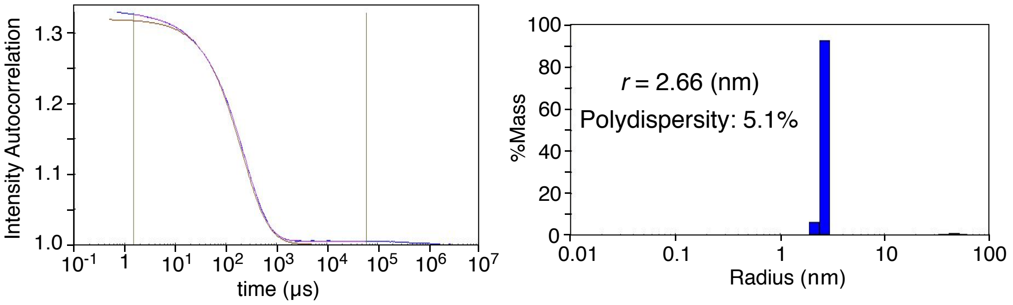

(f) [6]catenane $[Ag_{24}(\mathbf{5K})_{24}](OTf)_{24}$

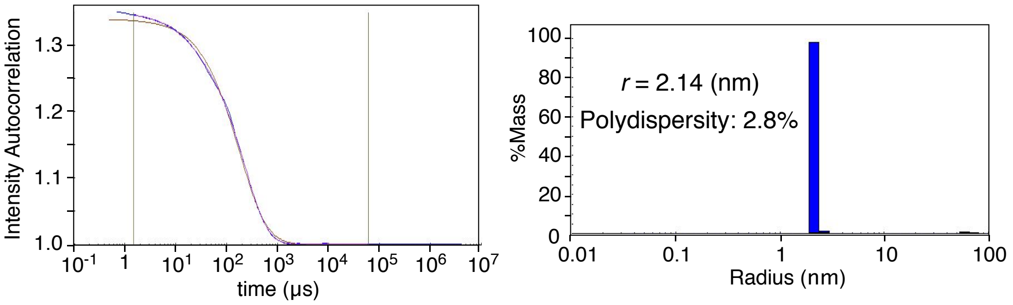

Supplementary Figure 42 (continues) |

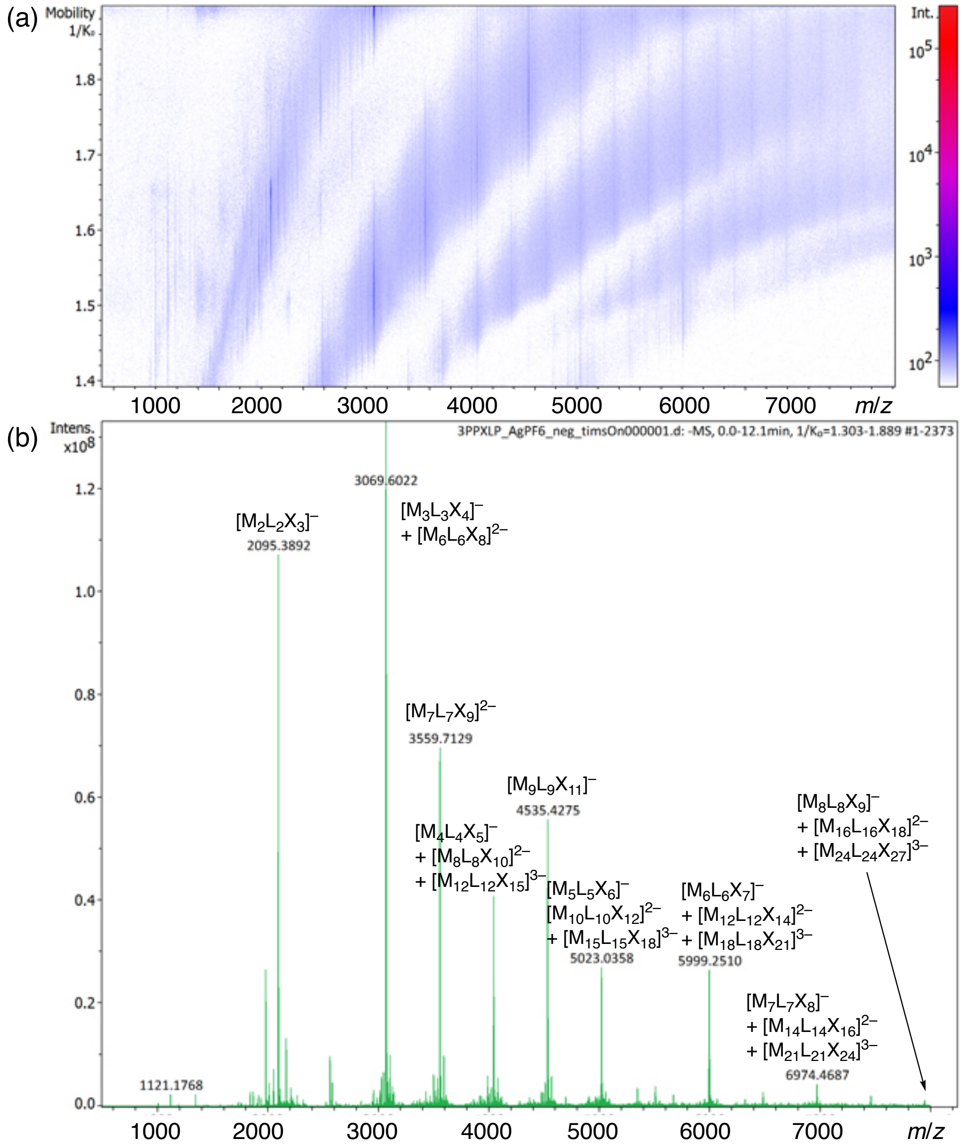

Supplementary Figure 43 |

(a) TIMS heatmap and (b) overall ESI-TOF-MS spectrum (M: Ag, L: $C_{39}H_{46}N_8O_6$, and X: PF_6). A series of fragment ions with the same charge state were observed in the heatmap. Because of the labile nature of the py–Ag^+ coordination bond, fragmentation was inevitably observed.

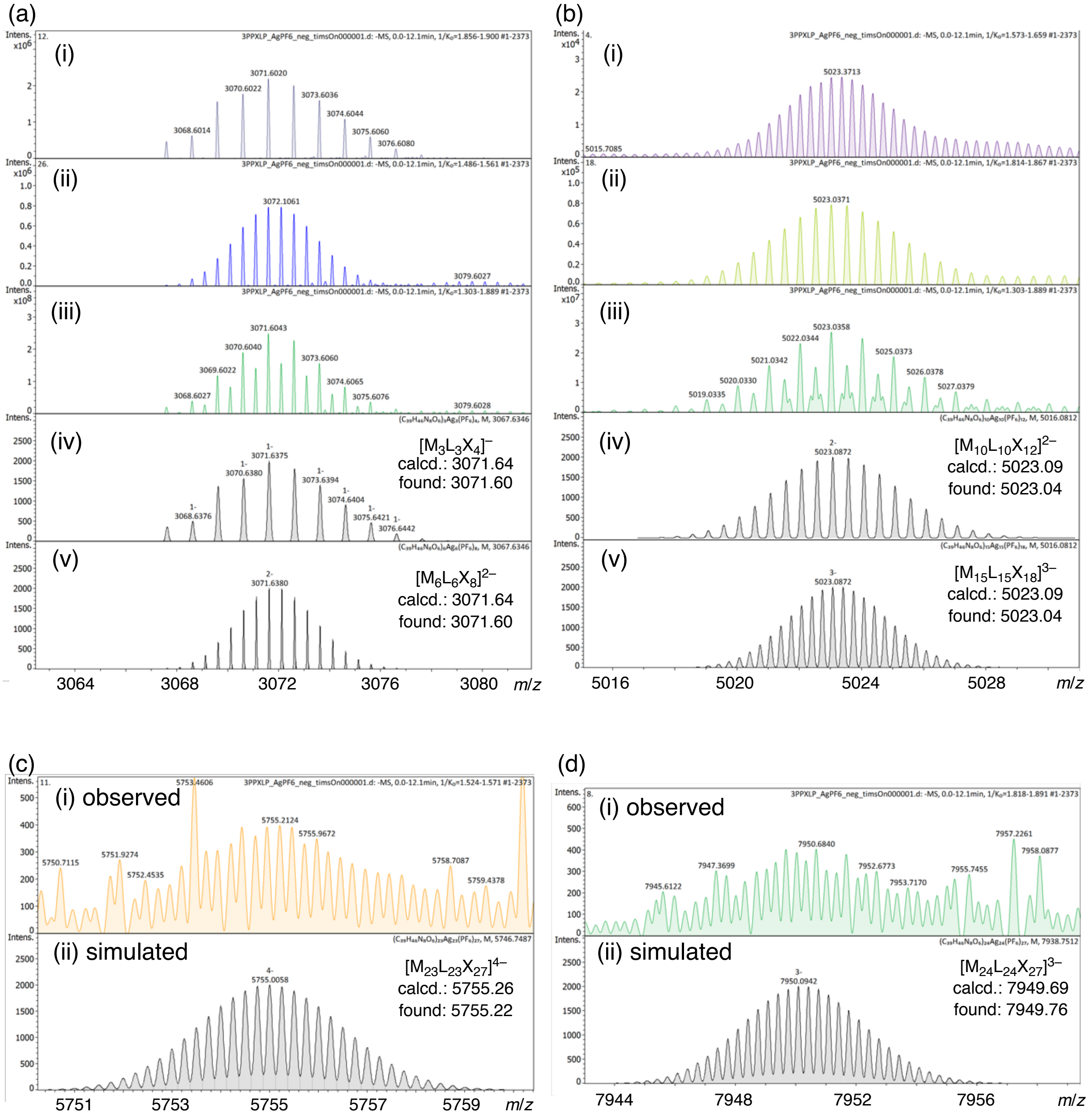

Supplementary Figure 44 |

Selected mass spectra of (a) $[M_3L_3X_4]^{-}$ and $[M_6L_6X_8]^{2-}$, (b) $[M_{10}L_{10}X_{12}]^{2-}$ and $[M_{15}L_{15}X_{18}]^{3-}$, (c) $[M_{23}L_{23}X_{27}]^{4-}$, and (d) $[M_{24}L_{24}X_{27}]^{3-}$ separated by TIMS. In (a) and (b), (i) and (ii) shows observed spectra in a certain region of the TIMS heatmap, (iii) shows integrated spectra of the same *m*/*z* region, and (iv) and (v) shows calculated patterns. In (c) and (d), (i) shows observed spectra and (ii) shows calculated patterns. Note that spectra of (c) $[M_{23}L_{23}X_{27}]^{4-}$ and (d) $[M_{24}L_{24}X_{27}]^{3-}$ were observed at low intensities because of severe fragmentation. (M: Ag, L: $C_{39}H_{46}N_8O_6$, and X: PF_6).

Complexation of ligand mixtures

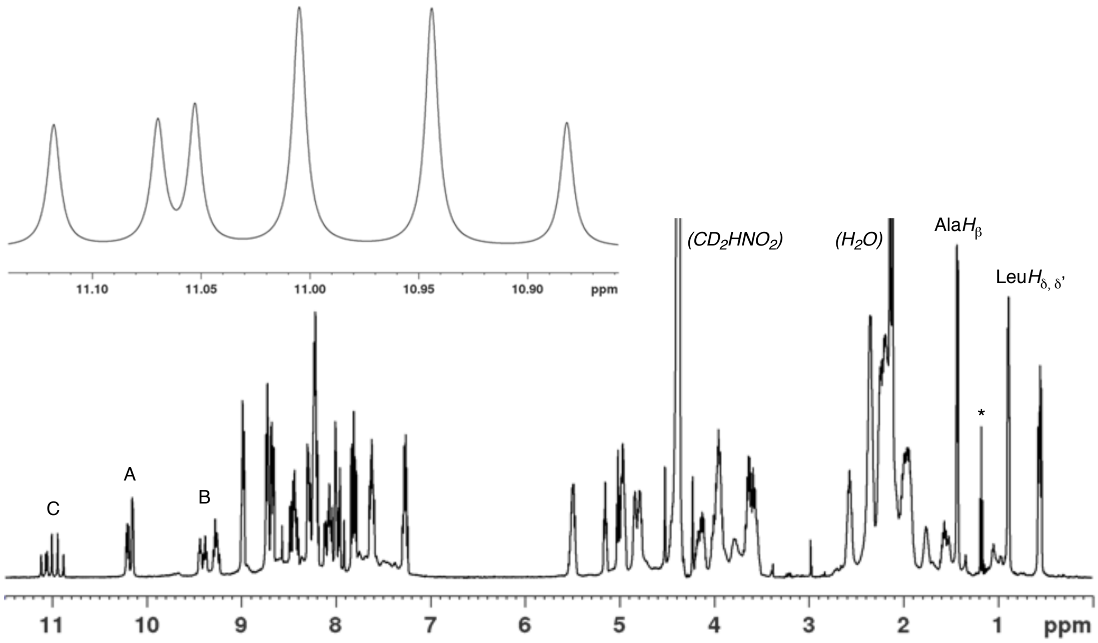

Supplementary Figure 45 |

^{1}H NMR spectrum (500 MHz, CD_3NO_2, 300 K) after complexation of **5** + **5L** + $AgPF_6$. [**5**] = [**5L**] = 10 mM and [$AgPF_6$] = 20 mM. The 10.8–11.2 ppm region is enlarged at the top. A signal with an asterisk indicates a solvent.

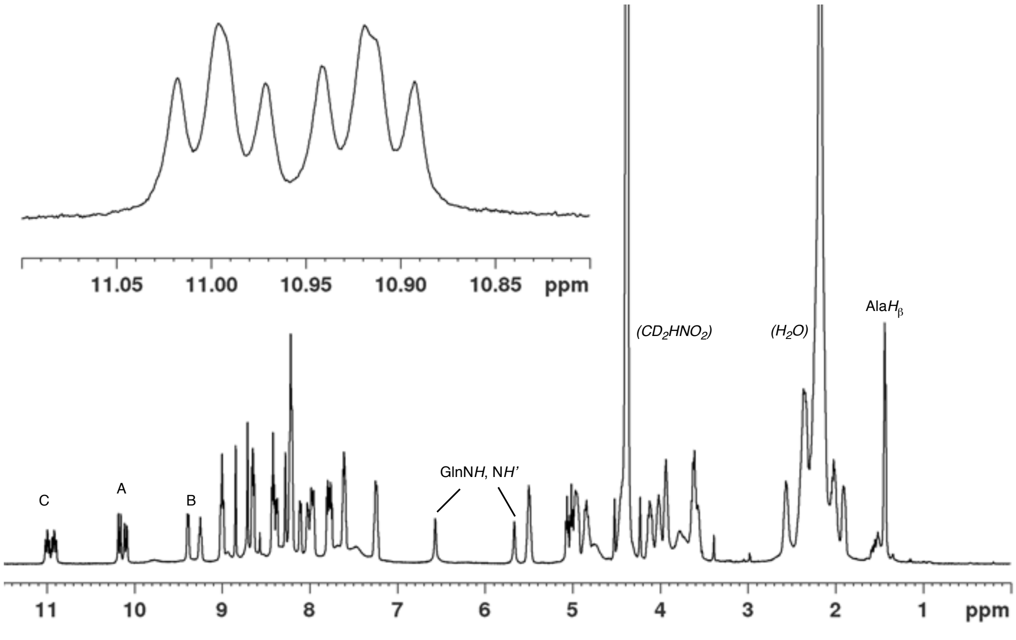

Supplementary Figure 46 |

^{1}H NMR spectrum (500 MHz, CD_3NO_2, 300 K) after complexation of **5** + **5Q** + $AgPF_6$. [**5**] = [**5Q**] = 10 mM and [$AgPF_6$] = 20 mM. The 10.9–11.1 ppm region is enlarged at the top.

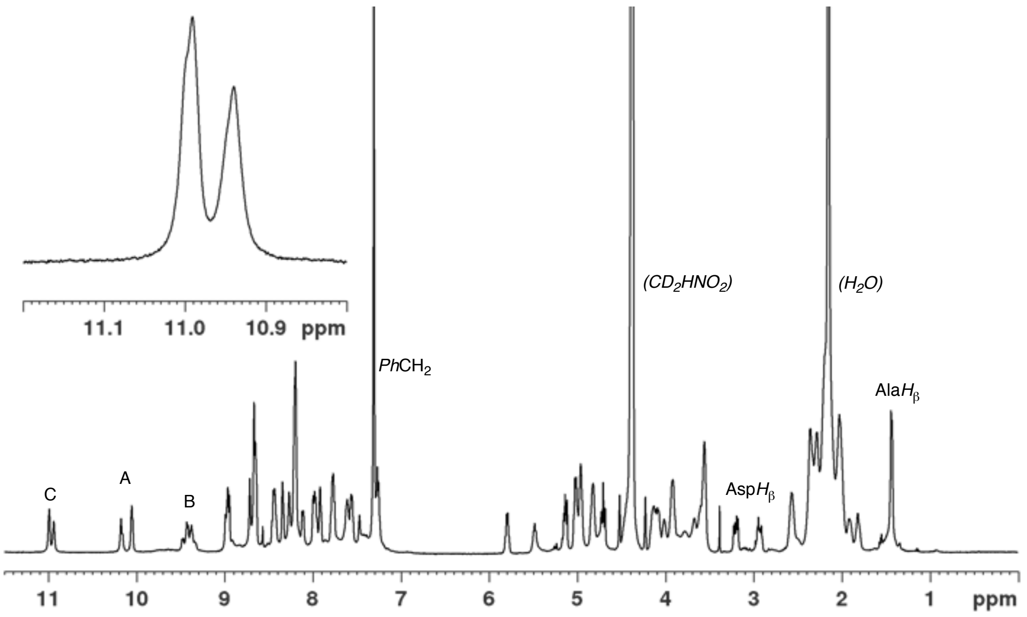

Supplementary Figure 47 |
^{1}H NMR spectrum (500 MHz, CD_3NO_2, 300 K) after complexation of **5** + **5D** + $AgPF_6$. [**5**] = [**5D**] = 10 mM and [$AgPF_6$] = 20 mM. The 10.8–11.2 ppm region is enlarged at the top.

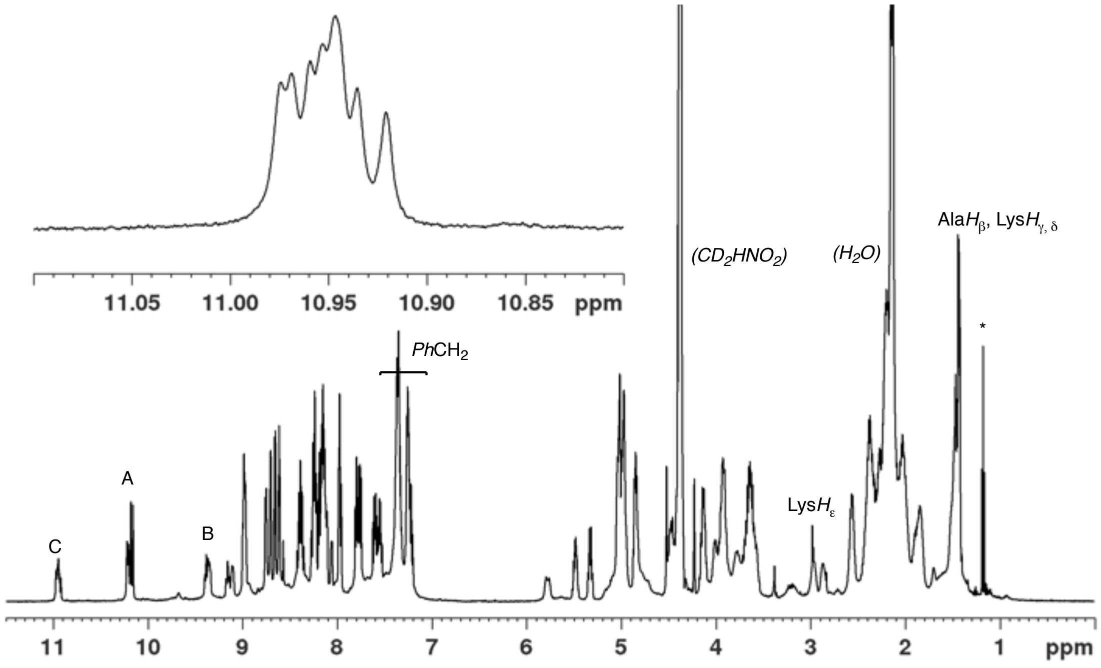

Supplementary Figure 48 |
^{1}H NMR spectrum (500 MHz, CD_3NO_2, 300 K) after complexation of **5** + **5K** + $AgPF_6$. [**5**] = [**5K**] = 10 mM and [$AgPF_6$] = 20 mM. The 10.8–11.2 ppm region is enlarged at the top. A signal with an asterisk indicates a solvent.

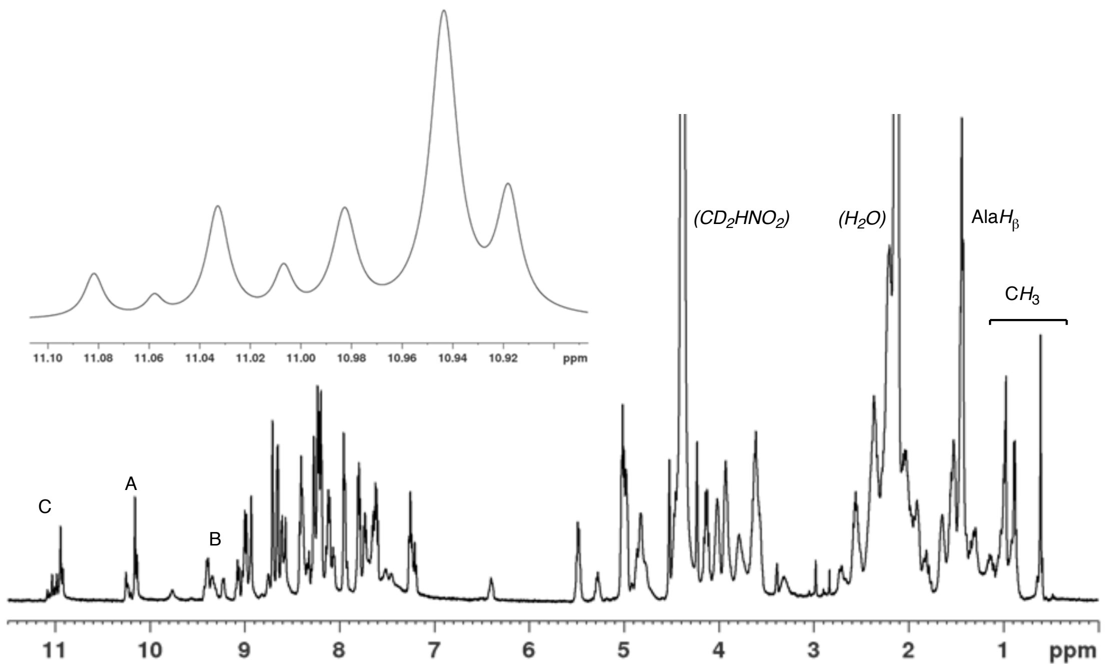

Supplementary Figure 49 |

^{1}H NMR spectrum (500 MHz, CD_3NO_2, 300 K) after complexation of **5** + **7** + $AgPF_6$. [**5**] = 7.5 mM, [**7**] = 2.5 mM, and [$AgPF_6$] = 10 mM. The 10.9–11.1 ppm region is enlarged at the top.

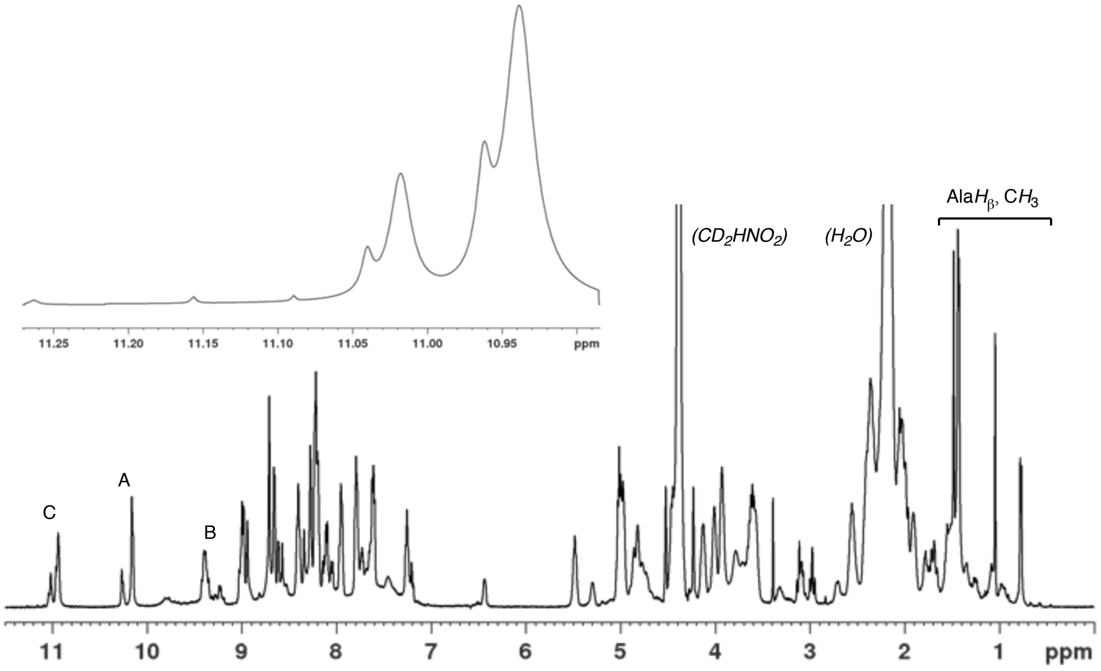

Supplementary Figure 50 |

^{1}H NMR spectrum (500 MHz, CD_3NO_2, 300 K) after complexation of **5** + **8** + $AgPF_6$. [**5**] = 7.5 mM, [**8**] = 2.5 mM, and [$AgPF_6$] = 10 mM. The 10.9–11.3 ppm region is enlarged at the top.

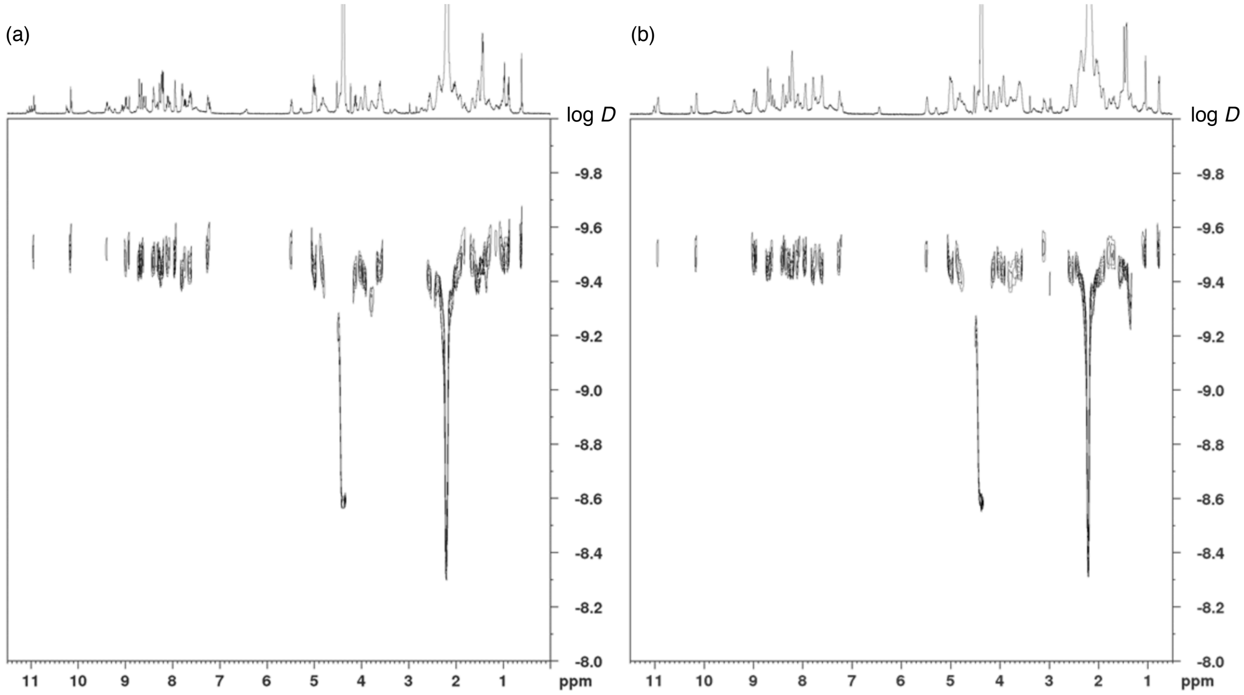

Supplementary Figure 51 |

^{1}H DOSY NMR spectra (500 MHz, CD_3NO_2, 300 K) after complexation of (a) **5** + **7** + $AgPF_6$ ($D = 3.2\times10^{-10}$ m^2•s^{-1}) and (a) **5** + **8** + $AgPF_6$ ($D = 3.3\times10^{-10}$ m^2•s^{-1}). [**5**] = 7.5 mM, [**7**] or [**8**] = 2.5 mM, and [$AgPF_6$] = 10 mM. The same diffusion constant among aromatic/amide signals of **5** and aliphatic signals of **7** or **8** shows the successful formation of [6]catenanes. Note that all the signals are desymmetrized due to the ligand-swapping on the [6]catenane framework.

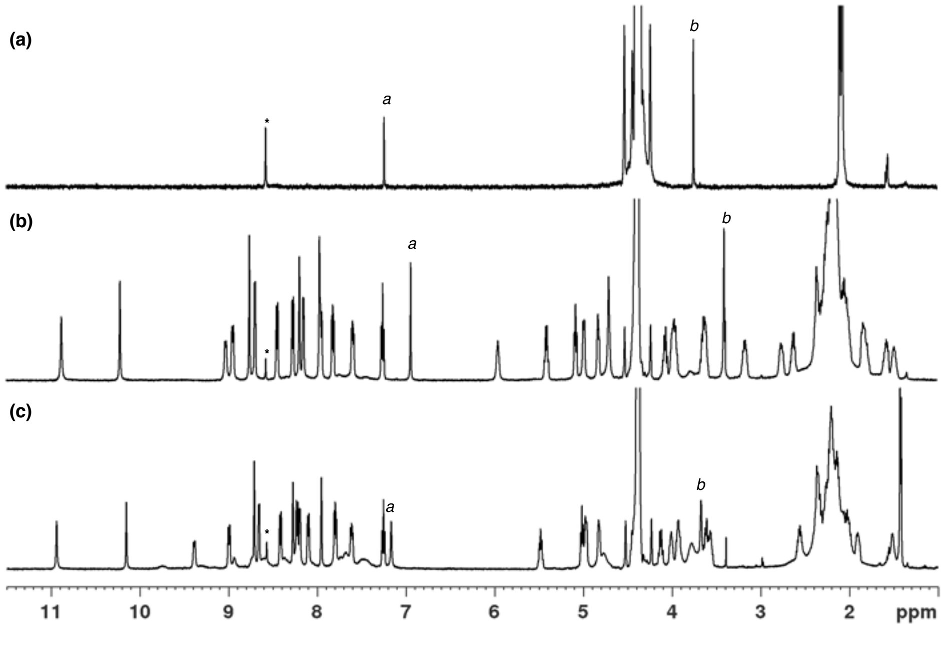

Supplementary Figure 52 |

^{1}H NMR spectra (500 MHz, CD_3NO_2, 300 K) of (a) 1,3,5-benzenetriacetic acid (**G**), (b) $[Ag_{24}(\mathbf{9})_{24}](Tf_2N)_{24}$ + **G**, and (c) **6**•$(Tf_2N)_{24}$ + **G**. $[Ag_{24}(\mathbf{9})_{24}]^{24+}$ = [**6**] = 0.42 mM and [**G**] = 2.3 mM (5.5 equiv. to the host). Signals with asterisks derive from the solvent.

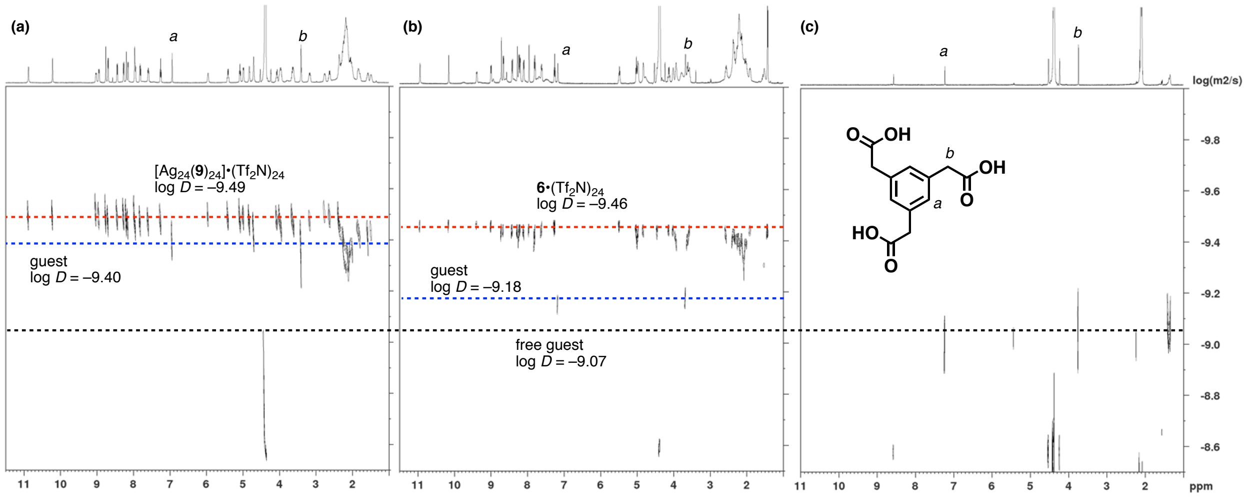

Supplementary Figure 53 |

^{1}H DOSY NMR spectra (500 MHz, CD_3NO_2, 300 K) of (a) $[Ag_{24}(\mathbf{9})_{24}](Tf_2N)_{24}$ + **G** ($D_{complex}$ = 3.3×10^{-10} m^2•s^{-1}, D_{guest} = 3.9×10^{-10} m^2•s^{-1}), (b) **6**•$(Tf_2N)_{24}$ + **G** ($D_{complex}$ = 3.4×10^{-10} m^2•s^{-1}, D_{guest} = 6.6×10^{-10} m^2•s^{-1}) and (c) 1,3,5-benzenetriacetic acid **G** (*D* = 8.8×10^{-10} m^2•s^{-1}). $[Ag_{24}(\mathbf{9})_{24}]^{24+}$ or [**6**] = 0.42 mM and [**G**] = 2.2 mM. Guest signals in (a) showed the smaller diffusion constant to (c), which indicates the shift of the equilibration state toward the formation of host–guest complex $[Ag_{24}(\mathbf{9})_{24}]^{24+}$•$(\mathbf{G})_n$. Note that the diffusion constant of the guest here was the average of free guests and host–guest complexes due to the fast equilibrium. Larger diffusion constant of the guest signals in (b) indicates that **6** has less ability to encapsulate **G** than $[Ag_{24}(\mathbf{9})_{24}]^{24+}$.

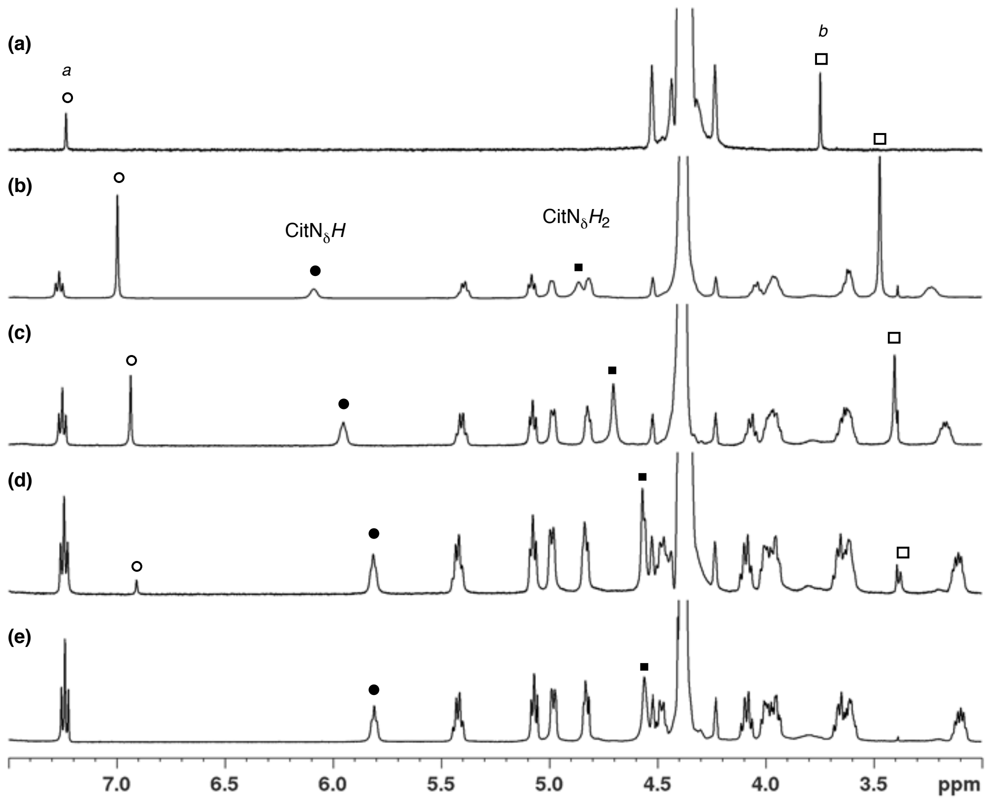

Supplementary Figure 54 |

^{1}H NMR spectra (500 MHz, CD_3NO_2, 300 K) of (a) 1,3,5-benzenetriacetic acid (**G**), (b-d) $[Ag_{24}(\mathbf{9})_{24}](Tf_2N)_{24}$ + **G**, and (e) $[Ag_{24}(\mathbf{9})_{24}](Tf_2N)_{24}$. $[Ag_{24}(\mathbf{9})_{24}]^{24+}$ = 0.42 mM, and [**G**] = (b) 6.0 mM, (c) 2.3 mM, and (d) 0.42 mM. Circles and squares indicate the signals of **G**. Black dots and rectangulars indicate the urea signals of $[Ag_{24}(\mathbf{9})_{24}]^{24+}$.

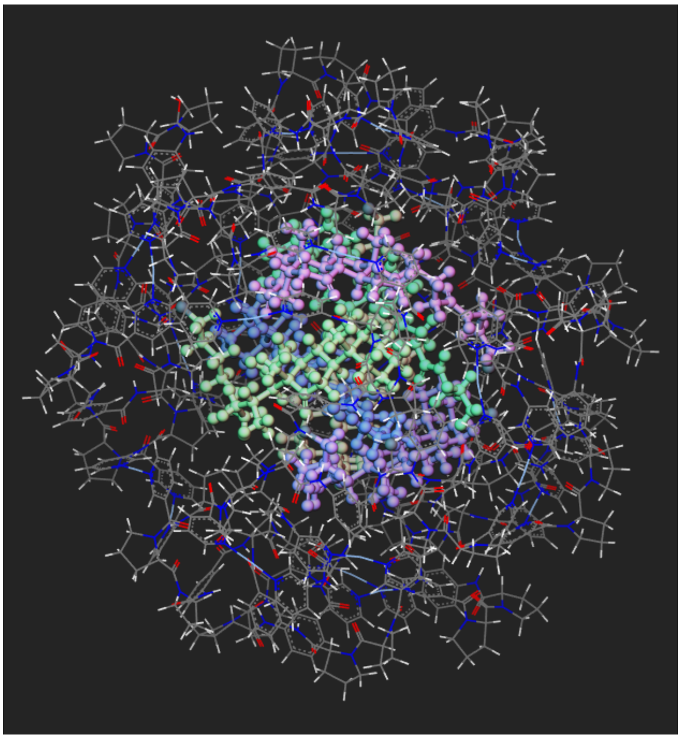

Supplementary Figure 55 |
Modeling structure of [Ag_{24}(**5**)$_{18}$(**7**)$_6$]. The [6]catenane framework was modeled by using 24 Ag^+ ions, 6 units of guest-tethered ligand **7**, and 18 units of ligand **5**. Each lithocholic acid is highlighted in colour-coded ball and stick representations. Atoms except for the lithocholic acid moiety are shown as line for clarity. It was confirmed that the inner cavity volume was large enough to load six molecules of lithocholic acid or dehydrocholic acid. Molecular modeling was performed in BIOVIA Materials studio 2018. Molecular mechanics calculation (forcite: *Universal*) was applied for the geometry optimization of the complex and guest molecules.

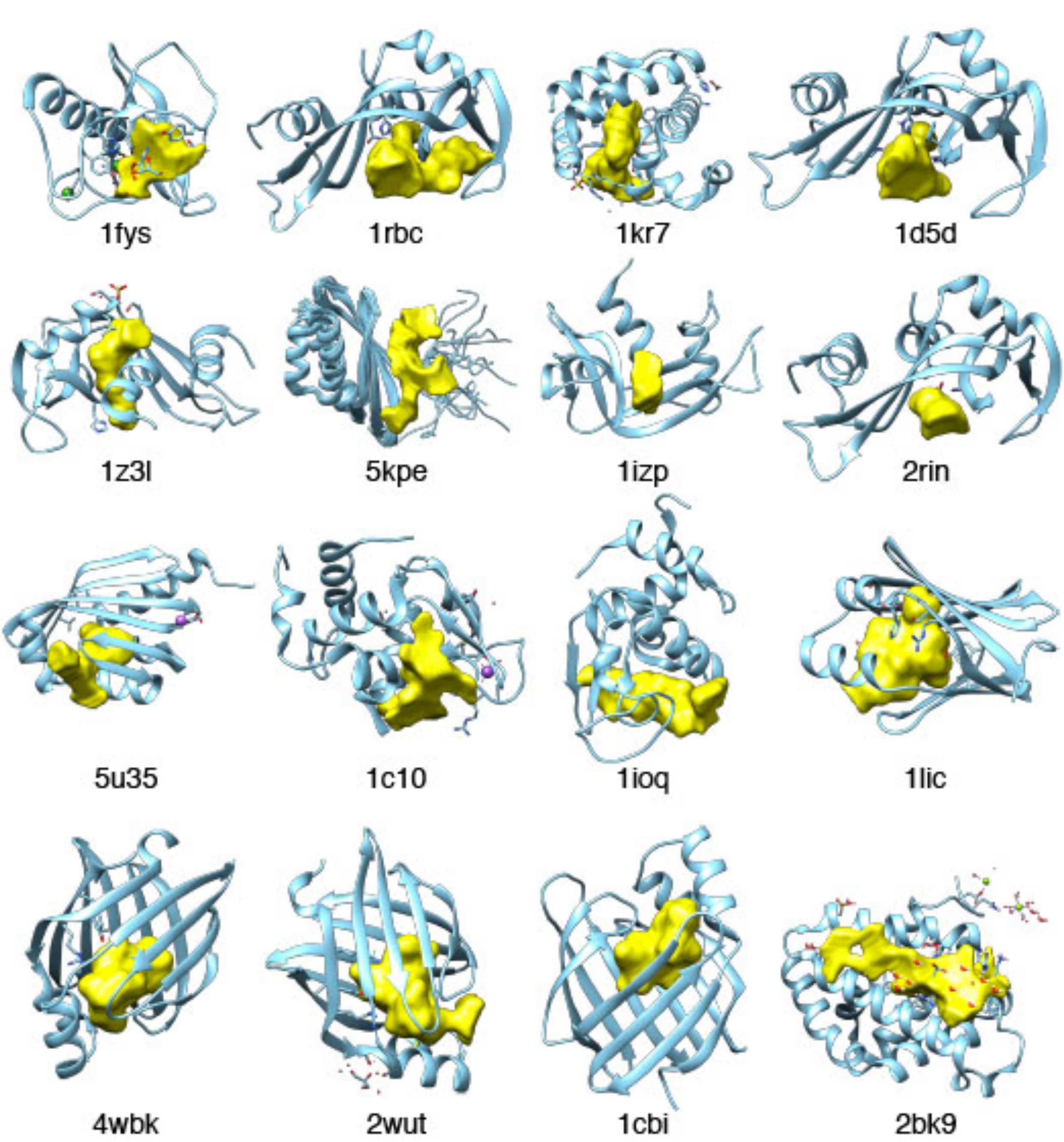

Supplementary Figure 56 |

Graphical representation of calculated cavities listed in Supplementary Table 6. Calculated cavities are shown as yellow surface, each protein is drawn by cyan cartoon, and the corresponding PDB ID is shown below. Graphics were generated with UCSF Chimera.[4]

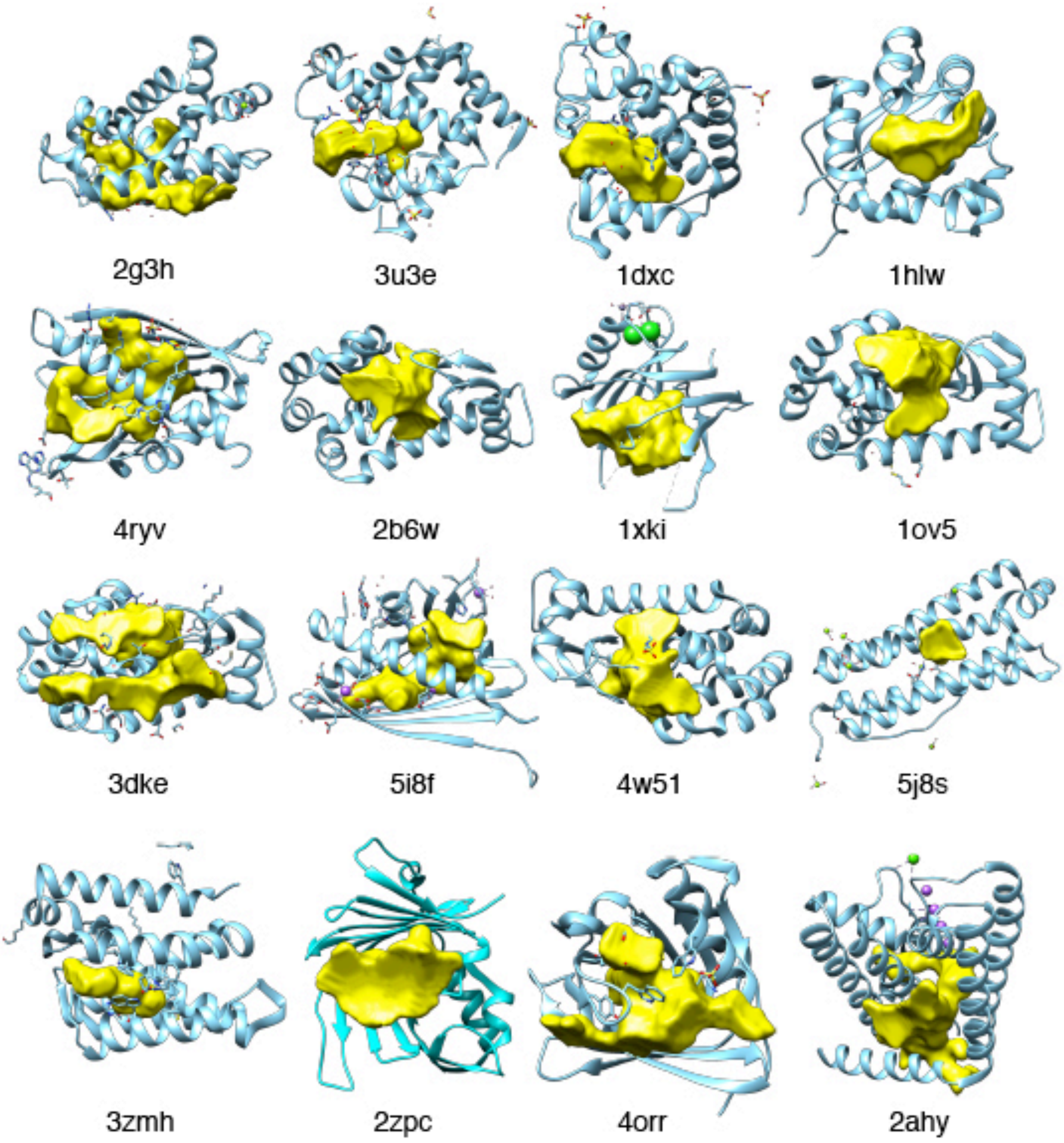

Supplementary Figure 56 (continues) |

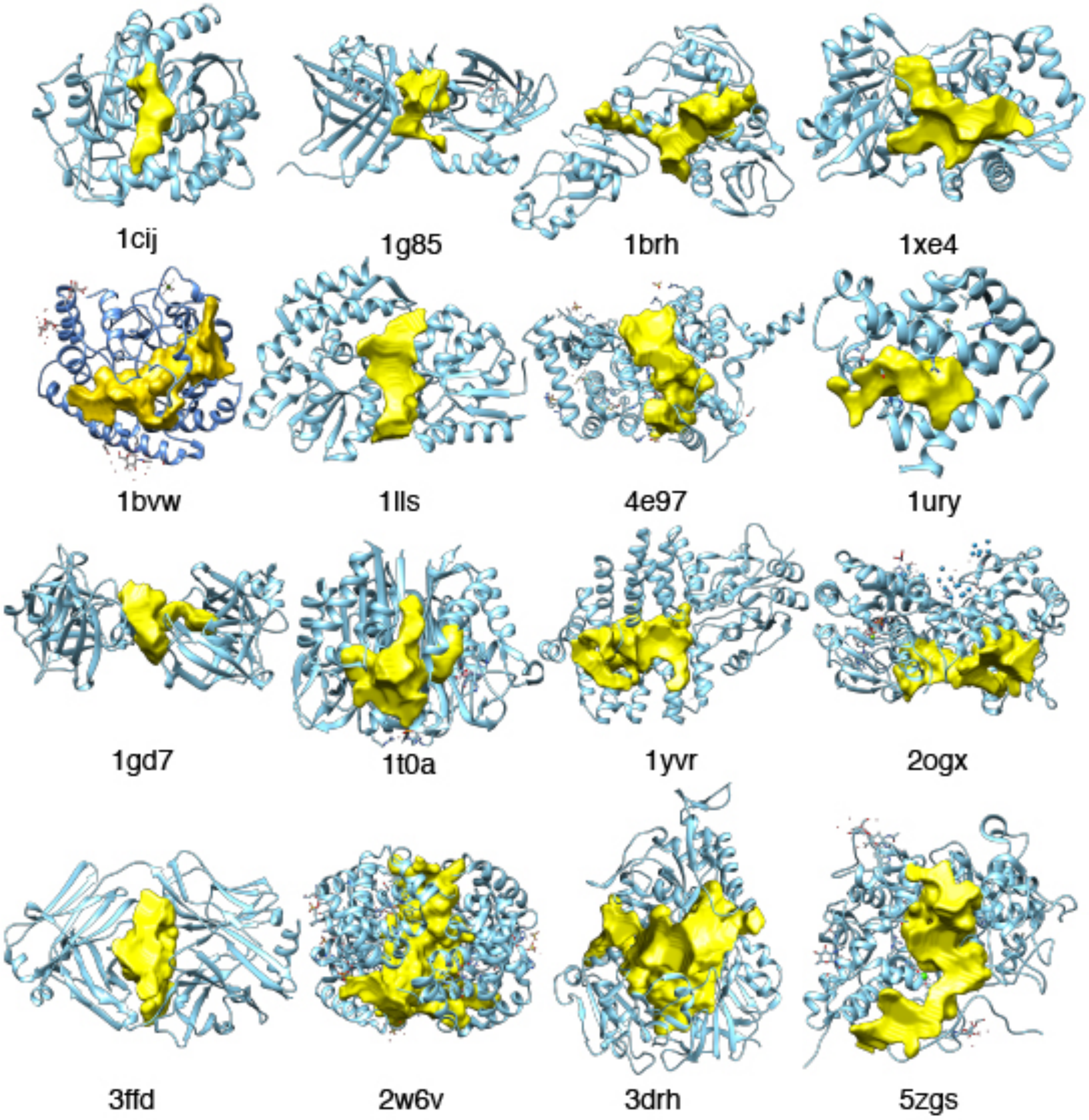

Supplementary Figure 57 |

Graphical representation of calculated cavities listed in Supplementary Table 7. Calculated cavities are shown as yellow surface, each protein is drawn by cyan cartoon, and the corresponding PDB ID is shown below. Graphics were generated with UCSF Chimera.[4]

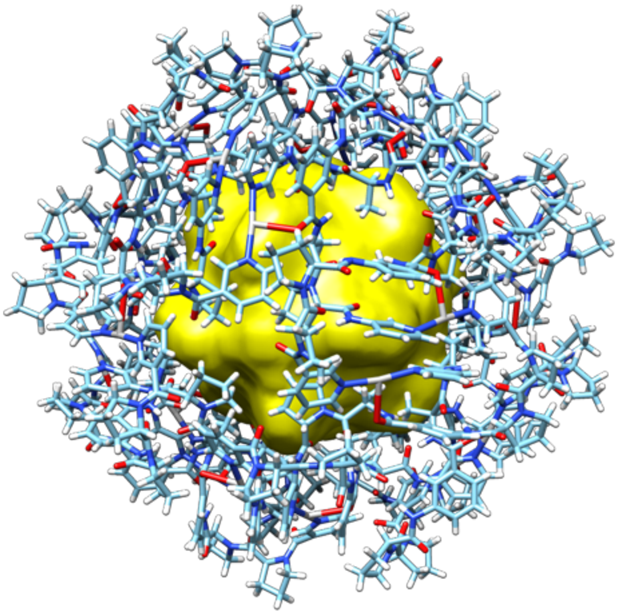

Supplementary Figure 58 |

Cavity volume of [6]catenane **6** calculated by using 3V webserver.[5] Coordinates of crystal structure A (CCDC 1881307), the outer probe radius of 8.0 Å, the inner probe radius of 1.25 Å, and the grid size of 0.5 Å were used. The framework of **6** are represented as stick and the cavity is shown as yellow surface. The cavity volume was estimated as 3155 Å^3. Graphics were generated with UCSF Chimera.[4]

Supplementary Notes

Supplementary note 1 |

Response to the alerts in the crystallographic data.

The final cif files were checked using IUCr's checkcif algorithm. Due to the characteristics of the crystals with large unit cells, large intermolecular spaces filled with disordered counter anions and solvent molecules, and poor diffraction, a number of A-level and B-level alerts remain in the checkcif files. These are inevitable in this class of the large supramolecular compound. The response to individual alerts were shown below.

Response to the alerts involved in the crystallographic data of **6** (crystal structure A, CCDC 1881307):

THETM01_ALERT_3_A The value of sine(theta_max)/wavelength is less than 0.550
PLAT084_ALERT_3_B High wR2 Value (i.e. > 0.25)
PLAT201_ALERT_2_A Isotropic non-H Atoms in Main Residue(s)

All these alerts are because of the poor quality of the crystal. Multiple attempts of crystal preparations and data collection did not succeed. This poor data quality was common in this class of very large molecules with large intermolecular spaces filled with disordered anions and solvent molecules. In order to maximize the data/parameter ratio, only Ag atoms were anisotropically refined. However, the structure was determined to a level satisfactory for the interpretation in this paper.

PLAT342_ALERT_3_B Low Bond Precision on C-C Bonds
PLAT414_ALERT_2_B Short Intra D-H..H-X H7AC..H30A
PLAT430_ALERT_2_B Short Inter D...A Contact O10Q..O11Q
PLAT602_ALERT_2_A VERY LARGE Solvent Accessible VOID(S) in Structure

All these alerts are related to geometrical and atomic displacement parameters problems, that result from disordered moieties of the structure and severely disordered solvents and anions. Due to poor data quality, the discussion of accurate bond lengths contains imperfectness. However, the molecular topology is clearly apparent and the data quality doesn't affect the discussion of supramolecular structure.

PLAT306_ALERT_2_B Isolated Oxygen Atom (H-atoms Missing ?)

These oxygen atoms were the solvent water and the structure model was refined without hydrogen atoms on the water.

PLAT987_ALERT_1_B The Flack x is >> 0 - Do a BASF/TWIN Refinement

This alert is due to the poor data quality. The starting material of peptide ligand **5** was chiral amino acids and synthesized without any epimerization, so the absolute stereochemistry is unambiguously determined. Although we checked the twin law by PLATON software, no twin law was detected.

Response to the alerts involved in the crystallographic data of **6** (crystal structure B, CCDC 1881308):

THETM01_ALERT_3_A The value of sine(theta_max)/wavelength is less than 0.550
PLAT082_ALERT_2_B High R1 Value
PLAT084_ALERT_3_A High wR2 Value (i.e. > 0.25)
PLAT201_ALERT_2_A Isotropic non-H Atoms in Main Residue(s)
PLAT934_ALERT_3_B Number of (Iobs-Icalc)/SigmaW > 10 Outliers

All these alerts are because of the poor quality of the crystal. Multiple attempts of crystal preparations and data collection did not succeed, despite using the synchrotron radiation. This poor data quality was common in very large molecules with large intermolecular spaces filled with disordered anions and solvent molecules. In order to maximize the data/parameter ratio, only Ag atoms were anisotropically refined. However, the structure was determined to a level satisfactory for the interpretation in this paper.

PLAT026_ALERT_3_B   Ratio Observed / Unique Reflections (too) Low
PLAT029_ALERT_3_A   _diffrn_measured_fraction_theta_full value Low
PLAT911_ALERT_3_B   Missing FCF Refl Between Thmin & STh/L=   0.502

All these alerts are due to the limited data collection method at the synchrotron beamline used. Only phi scans were conducted, and the fragility of the crystal prevented additional data set collection.

PLAT049_ALERT_1_B   Calculated Density Less Than 1.0 gcm-3
PLAT241/242_ALERT_2_B   High or Low 'MainMol' Ueq as Compared to Neighbors
PLAT342_ALERT_3_B   Low Bond Precision on C-C Bonds
PLAT360_ALERT_2_B   Short C(sp3)-C(sp3) Bond
PLAT362/363_ALERT_2_B   Short or Long C(sp3)-C(sp2) Bond
PLAT369_ALERT_2_B   Long C(sp2)-C(sp2) Bond
PLAT414_ALERT_2_B   Short Intra D-H..H-X
PLAT430_ALERT_2_B   Short Inter D...A Contact
PLAT602_ALERT_2_A   VERY LARGE Solvent Accessible VOID(S) in Structure

All these alerts are related to geometrical and atomic displacement parameters problems, that result from disordered moieties of the structure and severely disordered solvents and anions. Due to poor data quality, the discussion of accurate bond lengths contains imperfectness. However, the molecular topology is clearly apparent and the data quality doesn't affect the discussion of supramolecular structure.

PLAT306_ALERT_2_B   Isolated Oxygen Atom (H-atoms Missing ?)

These oxygen atoms were the solvent water and the structure model was refined without hydrogen atoms on the water.

Supplementary Tables

Supplementary Table 1 |

Crystal data and structure refinement for **6** (crystal structure A). CCDC 1881307.

Identification code	inomata36	
Empirical formula	$C_{888}H_{1022}Ag_{24}N_{216}O_{288}$	
Formula weight	21918.04	
Temperature	93(2) K	
Wavelength	0.71073 Å	
Crystal system	Cubic	
Space group	*I*432	
Unit cell dimensions	a = 40.552(3) Å	α = 90º
	b = 40.552(3) Å	β = 90º
	c = 40.552(3) Å	γ = 90º
Volume	66684(12) $Å^3$	
Z	2	
Density (calculated)	1.092 Mg•m^{-3}	
Absorption coefficient	0.416 mm^{-1}	
F_{000}	22588	
Crystal size	0.12 × 0.10 × 0.07 mm^3	
θ range for data collection	1.42 to 20.91º	
Index ranges	$-40 \leq h \leq 40, -40 \leq k \leq 40, -40 \leq l \leq 40$	
Reflections collected	223544	
Independent reflections	5944 [R_{int} = 0.1642]	
Completeness to θ = 20.91º	99.9%	
Absorption correction	SADABS	
Refinement method	Full-matrix least-squares on F^2	
Data / restraints / parameters	5944 / 79 / 322	
Goodness-of-fit on F^2	1.483	
Final R indices [$I > 2\sigma(I)$]	R_1 = 0.1511, wR_2 = 0.4036	
R indices (all data)	R_1 = 0.1844, wR_2 = 0.4500	
Absolute structure parameter	0.057(13)	
Largest diff. peak and hole	0.892 and –0.958 e.$Å^{-3}$	

Supplementary Table 2 |

Crystal data and structure refinement for **6** (crystal structure B). CCDC 1881308.

Identification code	YI478-4-6	
Empirical formula	$C_{866}H_{992}Ag_{24}N_{194}O_{170}$	
Formula weight	19427.36	
Temperature	293(2) K	
Wavelength	1.00000 Å	
Crystal system	monoclinic	
Space group	*I*2	
Unit cell dimensions	a = 38.0379(4) Å	α = 90º
	b = 41.2103(5) Å	β = 92.230(1)º
	c = 47.8492(7) Å	γ = 90º
Volume	74949.4(16) Å^3	
Z	2	
Density (calculated)	0.861 Mg•m^{-3}	
Absorption coefficient	0.879 mm^{-1}	
F_{000}	20068	
Crystal size	0.15 × 0.14 × 0.05 mm^3	
θ range for data collection	1.55 to 30.16º	
Index ranges	$-37 \le h \le 36$, $-39 \le k \le 39$, $-40 \le l \le 47$	
Reflections collected	108488	
Independent reflections	62687 [R_{int} = 0.1547]	
Completeness to θ = 30.16º	81.1%	
Refinement method	Full-matrix least-squares on F^2	
Data / restraints / parameters	62687 / 529 / 2629	
Goodness-of-fit on F^2	1.132	
Final R indices [$I > 2\sigma(I)$]	R_1 = 0.1702, wR_2 = 0.4039	
R indices (all data)	R_1 = 0.2930, wR_2 = 0.4759	
Largest diff. peak and hole	1.213 and –0.779 e.Å^{-3}	

Supplementary Table 3 |

A list of *m*/*z* peaks of the same charge state (M: Ag, L: $C_{39}H_{46}N_8O_6$, and X: PF_6).

Observed species	Observed *m*/*z*	Intensity	*S*/*N*	Calcd. *m*/*z*	Error (ppm)
Selected peaks in the 1– charge state from the overall spectrum					
[$M_2L_2X_3$]	2095.3892	$1.07×10^8$	1847251.9	2095.4122	10.98
[$M_3L_3X_4$]	3071.6043	$2.50×10^8$	4306891.0	3071.6380	10.97
[$M_4L_4X_5$]	4047.8194	$4.09×10^7$	705341.8	4047.8627	10.70
[$M_5L_5X_6$]	5023.0358	$2.70×10^7$	466008.3	5023.0870	10.19
[$M_6L_6X_7$]	5999.2510	$2.65×10^7$	457586.8	5999.3120	10.17
[$M_7L_7X_8$]	6974.4687	$4.35×10^6$	74999.2	6974.5362	9.68
[$M_8L_8X_9$]	7949.6967	$1.23×10^6$	21239.2	7949.7604	8.01
Selected peaks in the 2– charge state					
[$M_5L_5X_7$][a]	2583.9984	$4.68×10^6$	80745.9	2584.0259	10.64
[$M_6L_6X_8$]	3072.1061	$7.92×10^5$	6427.9	3072.1384	10.51
[$M_7L_7X_9$]	3559.7128	$3.29×10^5$	1401.3	3559.7505	10.57
[$M_8L_8X_{10}$]	4047.8199	$1.38×10^5$	621.6	4047.8630	10.63
[$M_9L_9X_{11}$]	4535.4273	$2.51×10^5$	538.1	4535.4751	10.52
[$M_{10}L_{10}X_{12}$]	5023.0371	$7.84×10^4$	192.7	5023.0872	9.98
[$M_{11}L_{11}X_{13}$][a]	5511.1445	$3.87×10^6$	66807.2	5511.1996	10.00
[$M_{12}L_{12}X_{14}$][a]	5998.7506	$5.63×10^6$	97032.8	5998.8117	10.19
[$M_{13}L_{13}X_{15}$][a]	6486.8596	$2.73×10^6$	47051.0	6486.9242	9.96
[$M_{14}L_{14}X_{16}$][a]	6974.9656	$1.96×10^6$	33774.3	6975.0357	10.05
[$M_{15}L_{15}X_{17}$][a]	7462.0766	$2.08×10^6$	35787.0	7462.1484	9.62
[$M_{16}L_{16}X_{18}$][a]	7950.1868	$9.62×10^5$	16578.2	7949.2598	9.18
Selected peaks in the 3– charge state					
[$M_{12}L_{12}X_{15}$]	4047.8202	6751	448.3	4047.8619	10.29
[$M_{13}L_{13}X_{16}$]	4372.8948	9832	182.8	4372.9366	9.58
[$M_{14}L_{14}X_{17}$]	4697.9652	8470	68.9	4698.0114	9.83
[$M_{15}L_{15}X_{18}$]	5023.3713	24586	81.6	5023.4208	9.86
[$M_{16}L_{16}X_{19}$]	5348.7766	15429	37.2	5348.8280	9.60
[$M_{17}L_{17}X_{20}$]	5673.5163	8997	14.7	5673.5692	9.33
[$M_{18}L_{18}X_{21}$]	5999.2544	12326	31.0	5999.3111	9.46
[$M_{19}L_{19}X_{22}$]	6323.9900	5063	18.0	6324.0522	9.84
[$M_{20}L_{20}X_{23}$]	6649.7298	1999	10.4	6649.7942	9.68
[$M_{21}L_{21}X_{24}$]	6974.4688	2169	12.5	6974.5353	9.53
[$M_{22}L_{22}X_{25}$]	7299.8701	709	9.7	7299.9436	10.08
[$M_{23}L_{23}X_{26}$]	7625.2757	394	9.2	7625.3520	10.00
[$M_{24}L_{24}X_{27}$]	7949.6818	403	13.7	7949.7606	9.91

a) picked from the overall spectrum

Supplementary Table 4 |

Selected mass information characterized as the $[M_{24}L_{24}X_{27}]^{3-}$ ion peak.
(M: Ag, L: $C_{39}H_{46}N_8O_6$, and X: PF_6).

Selected mass information in the $[M_{24}L_{24}X_{27}]^{3-}$ peak						
Observed ion (*m*/*z*)	Intensity	*S*/*N*	FWHM	Resolution	Calcd. *m*/*z*	Error (ppm)
7945.3883	168	5.72	0.19863	40000	7945.4241	4.50
7945.6122	207	7.06	0.19864	40000	7945.7577	18.31
7945.9820	188	6.39	0.19865	40000	7946.0912	13.74
7946.3411	117	4	0.19866	40000	7946.4248	10.53
7946.6848	160	5.44	0.19867	40000	7946.7584	9.26
7947.0305	181	6.17	0.19868	40000	7947.0919	7.74
7947.3699	303	10.33	0.19868	40000	7947.4255	7.00
7947.7042	281	9.56	0.19869	40000	7947.7591	6.91
7948.0255	189	6.44	0.1987	40000	7948.0926	8.45
7948.3240	254	8.67	0.19871	40000	7948.4262	12.86
7948.7117	298	10.17	0.19872	40000	7948.7598	6.05
7949.0397	311	10.61	0.19873	40000	7949.0934	6.75
7949.3367	342	11.67	0.19873	40000	7949.4270	11.36
7949.6818	403	13.72	0.19874	40000	7949.7606	9.91
7950.0335	388	13.22	0.19875	40000	7950.0942	7.63
7950.3404	351	11.94	0.19876	40000	7950.4278	10.99
7950.6840	404	13.78	0.19877	40000	7950.7614	9.74
7951.0207	307	10.44	0.19878	40000	7951.0950	9.35
7951.3535	294	10	0.19878	40000	7951.4287	9.45
7951.6751	380	12.94	0.19879	40000	7951.7623	10.96
7952.0611	271	9.22	0.1988	40000	7952.0960	4.38
7952.3344	290	9.89	0.19881	40000	7952.4296	11.97
7952.6773	302	10.28	0.19882	40000	7952.7633	10.81
7953.0552	220	7.5	0.19883	40000	7953.0969	5.25
7953.3628	199	6.78	0.19883	40000	7953.4306	8.53
7953.7170	225	7.67	0.19884	40000	7953.7643	5.94
7954.0438	207	7.06	0.19885	40000	7954.0980	6.81
7954.3730	166	5.67	0.19886	40000	7954.4317	7.37
7954.6871	189	6.44	0.19887	40000	7954.7654	9.85
7955.0190	233	7.94	0.19888	40000	7955.0991	10.07
7955.2627	266	9.06	0.19888	40000	7955.4329	21.39

Supplementary Table 5 |

Schematic representation of C_3-symmetric [6]catenane species suggested in the case of **5** + **5L** + Ag^+ complexation. Topological diagrams are shown here for clarity. Red: **5** and cyan: **5L**. Note that candidates III and VI have a pair of enantiomers. Structures I, II, III, and VI show good agreement with the ^{1}H NMR signal pattern (see Fig. 7e). In the solution state, these species would exist as a mixture because of almost no energetic difference.

Candidates	I	II	III	IV	V	VI
overview	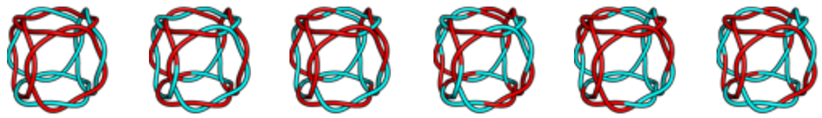					
C_3-axis view	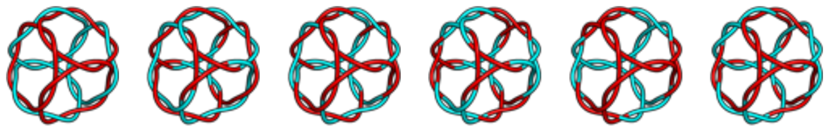					
Ring components	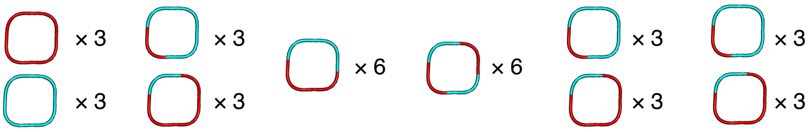 × 3<br>× 3	× 3<br>× 3	× 6	× 6	× 3<br>× 3	× 3<br>× 3
C_3 subunit components	Integral ratio of amide signals					
$(\mathbf{5})_3(\mathbf{5L})_0$	3 0	3 0	3 0	12 0	12 0	3 0
$(\mathbf{5})_2(\mathbf{5L})_1$	6 3	6 3	6 3	0 0	0 0	6 3
$(\mathbf{5})_1(\mathbf{5L})_2$	3 6	3 6	3 6	0 0	0 0	3 6
$(\mathbf{5})_0(\mathbf{5L})_3$	0 3	0 3	0 3	0 12	0 12	0 3

Supplementary Table 6 |

Cavity volume list of proteins with 100–200 chain lengths searched by PDB.

Entry	PDB ID	Volume (Å^3)	Chain Length	Entry	PDB ID	Volume (Å^3)	Chain Length
1	1fys	231	104	17	2g3h	1142	153
2	1rbc	567	104	18	3u3e	756	154
3	1kr7	833	110	19	1dxc	933	154
4	1d5d	606	116	20	1hlw	572	155
5	1z3l	346	119	21	4ryv	1340	155
6	5kpe	1343	120	22	2b6w	762	162
7	1izp	172	124	23	1xki	1107	162
8	2rln	176	125	24	1ov5	1006	164
9	5u35	629	125	25	3dke	1687	164
10	1c10	500	129	26	5i8f	1159	165
11	1ioq	725	129	27	4w51	930	172
12	1lic	909	131	28	5j8s	173	176
13	4wbk	84	133	29	3zmh	384	180
14	2wut	909	133	30	2zpc	835	190
15	1cbi	538	136	31	4orr	1422	190
16	2bk9	1041	153	32	2ahy	1607	220

Supplementary Table 7 |

Cavity volume list of proteins with 300–600 chain lengths searched by PDB.

Entry	PDB ID	Volume (Å^3)	Chain Length	Entry	PDB ID	Volume (Å^3)	Chain Length
1	1cij	348	310	9	1gd7	873	436
2	1g85	993	318	10	1t0a	1357	477
3	1brh	2471	330	11	1yvr	3845	538
4	1xe4	1810	335	12	2ogx	3067	546
5	1bvw	2401	360	13	3ffd	2352	546
6	1lls	1433	370	14	2w6v	7065	574
7	4e97	3231	374	15	3drh	7373	590
8	1ury	919	380	16	5zgs	3730	595

Supplementary Discussion

The link invariant of the [6]catenane framework topology shown in Fig. 4e was calculated by using KNOT program.[6]

Link invariant of compound **6** (L):

crossing number

$$c(L) = 24 \quad (1)$$

(total) linking number

$$lk(L) = -12 \quad (2)$$

Conway polynomial

$$\nabla_L = -384z^5 \quad (3)$$

Jones polynomial

$$\begin{aligned} V_L = {} & -t^{\frac{5}{2}} + 5t^{\frac{7}{2}} - 15t^{\frac{9}{2}} + 35t^{\frac{11}{2}} - 70t^{\frac{13}{2}} + 126t^{\frac{15}{2}} - 210t^{\frac{17}{2}} + 324t^{\frac{19}{2}} \\ & - 471t^{\frac{21}{2}} + 649t^{\frac{23}{2}} - 851t^{\frac{25}{2}} + 1049t^{\frac{27}{2}} - 1222t^{\frac{29}{2}} + 1328t^{\frac{31}{2}} - 1355t^{\frac{33}{2}} \\ & + 1265t^{\frac{35}{2}} - 1101t^{\frac{37}{2}} + 861t^{\frac{39}{2}} - 619t^{\frac{41}{2}} + 386t^{\frac{43}{2}} - 212t^{\frac{45}{2}} + 93t^{\frac{47}{2}} \\ & - 32t^{\frac{49}{2}} + 7t^{\frac{51}{2}} - t^{\frac{53}{2}} \quad (4) \end{aligned}$$

HOMFLY polynomial

$$\begin{aligned} P_L(v, z) = {} & z^{-5}(-v^{-19} + 5v^{-21} - 10v^{-23} + 10v^{-25} - 5v^{-27} + v^{-29}) \\ & + z^{-3}(-12v^{-19} + 48v^{-21} - 72v^{-23} + 48v^{-25} - 12v^{-27}) \\ & + z^{-1}(-8v^{-17} - 34v^{-19} + 150v^{-21} - 166v^{-23} + 58v^{-25}) \\ & + z(-12v^{-15} - 39v^{-17} - 23v^{-19} + 211v^{-21} - 137v^{-23}) \\ & + z^{3}(-6v^{-11} - 18v^{-13} - 52v^{-15} - 72v^{-17} - 6v^{-19} + 154v^{-21}) \\ & + z^{5}(-v^{-5} - 5v^{-7} - 15v^{-9} - 35v^{-11} - 64v^{-13} - 96v^{-15} - 104v^{-17} - 64v^{-19}) \quad (5) \end{aligned}$$

Q-polynomial

$$\begin{aligned} Q(L) = {} & 32x^{-5} - 80x^{-4} - 304x^{-3} + 728x^{-2} + 1802x^{-1} - 3489 - 8136x + 10728x^{2} + 28904x^{3} \\ & - 21380x^{4} - 78192x^{5} + 21430x^{6} + 153926x^{7} + 15936x^{8} - 208464x^{9} - 90804x^{10} \\ & + 177596x^{11} + 140172x^{12} - 77136x^{13} - 111362x^{14} - 410x^{15} + 45956x^{16} \\ & + 15558x^{17} - 7576x^{18} - 5798x^{19} - 470x^{20} + 602x^{21} + 210x^{22} + 22x^{23} \quad (6) \end{aligned}$$

Supplementary References

1. Sawada, T., Inomata, Y., Yamagami, M., and Fujita, M. *Chem. Lett.* **46**, 1119–1121 (2017).
2. Banerjee, M., Das, S., Yoon, M., Choi, H. J., Hyun, M. H., Park, S. M., Seo, G., and Kim, K. *J. Am. Chem. Soc.* **131**, 7524–7525 (2009).
3. Sawada, T., Yamagami, M., Ohara, K., Yamaguchi, K., and Fujita, M. *Angew. Chem. Int. Ed.* **55**, 4519–4522 (2016).
4. Pettersen, E. F., Goddard, T. D., Huang, C. C., Couch, G. S., Greenblatt, D. M., Meng, E. C., and Ferrin, T. E., *J. Comput. Chem.* **13**, 1605–1612 (2004).
5. Voss, N. R. and Gerstain, M, *Nucleic acid Res.* **38**, W555–W562 (2010).
6. Kodama, K. KNOT program, http://www.math.kobe-u.ac.jp/HOME/kodama/knot.html
