# Supplementary material for: A metal–peptide capsule by multiple ring threading
Source: Nat Commun. 2019 Dec 12;10:5687. doi: 10.1038/s41467-019-13594-4 (PMC6908709; doi:10.1038/s41467-019-13594-4)
Supplement: Supplementary file 2 — Peer Review File [file 41467_2019_13594_MOESM2_ESM.pdf]

Editorial Note: This manuscript has been previously reviewed at another journal that is not operating a transparent peer review scheme. This document only contains reviewer comments and rebuttal letters for versions considered at Nature Communications .

Reviewers' Comments:

Reviewer #1:
Remarks to the Author:
The authors presented a systematic way to isolate complicated virus-like structures with big cavities. This was achieved based on previous findings (same group) and they also present the possibility of extending this domain to dodecahedron which highlights the importance of this work.

The obtained structured was extensively characterized and verified using all needed techniques (NMR, DOSY, SC)

They showed its tunability and the possibility to extend the functionality of the obtained cavity. They managed to isolate the reaction conditions which will consistently allow for the isolation of the same structure with different amino acids
while maintaining the same topology (hence the formation of isostructures).

They also showed the flexibility of the formed bonds which allows for the incorporation of guest molecules and interaction with the embedded functional groups.

The authors answered all previous concerns, therefore I recommend this paper for publication as it is.
